# Metabolic alterations in the absence of a neuromuscular phenotype in humanized *SOD1^A5V^* mice

**DOI:** 10.1093/braincomms/fcag302

**Published:** 2026-09-17

**Authors:** David Thompson, Chloe Williams, Georgia Price, Andrew P Tosolini, Clemens Falker-Gieske, Jonathan D Gilthorpe, Giampietro Schiavo, Elizabeth M C Fisher, Thomas J Cunningham

**Affiliations:** Mary Lyon Centre at MRC Harwell, Harwell OX11 0RD, UK; Nuffield Department of Clinical Neurosciences, University of Oxford, Oxford OX3 9DU, UK; Department of Medical and Translational Biology, Faculty of Medicine, Umeå University, Umeå 901 87, Sweden; Mary Lyon Centre at MRC Harwell, Harwell OX11 0RD, UK; Nuffield Department of Clinical Neurosciences, University of Oxford, Oxford OX3 9DU, UK; UCL Queen Square Motor Neuron Disease Centre and Department of Neuromuscular Diseases, UCL Queen Square Institute of Neurology, UCL, London WC1N 3BG, UK; UK Dementia Research Institute, University College London, London WC1N 3BG, UK; Department of Animal Sciences, Georg-August-University, Göttingen 37077, Germany; Department of Medical and Translational Biology, Faculty of Medicine, Umeå University, Umeå 901 87, Sweden; UCL Queen Square Motor Neuron Disease Centre and Department of Neuromuscular Diseases, UCL Queen Square Institute of Neurology, UCL, London WC1N 3BG, UK; UK Dementia Research Institute, University College London, London WC1N 3BG, UK; Mary Lyon Centre at MRC Harwell, Harwell OX11 0RD, UK; UCL Queen Square Motor Neuron Disease Centre and Department of Neuromuscular Diseases, UCL Queen Square Institute of Neurology, UCL, London WC1N 3BG, UK; Mary Lyon Centre at MRC Harwell, Harwell OX11 0RD, UK; Institute of Prion Diseases, MRC Prion Unit, UCL, London W1W 7FF, UK

**Keywords:** ALS, mouse models, gene targeting, SOD1

## Abstract

Amyotrophic lateral sclerosis caused by mutations in *superoxide dismutase 1* (*SOD1*) accounts for 15–30% of familial cases and is typically autosomal dominant. How single amino acid changes in this small protein cause neurodegeneration is unknown. In North America, *SOD1^A5V^* is the most common familial *SOD1* mutation and results in an aggressive form of amyotrophic lateral sclerosis. Here, we present a novel genomically humanized mouse model of *SOD1^A5V^*, in which the mouse *Sod1* locus has been replaced by the human *SOD1* gene, with intact genomic architecture of exons and introns, but bearing an A5 V mutation. In agreement with previously reported human genomic knock-in mice, the phenotype is mild; however, transcriptomic and metabolomic profiling reveal significant dysregulation of glycolysis, the tricarboxylic acid cycle, and lipid metabolism. These changes suggest an early bioenergetic imbalance that precedes neuromuscular impairment. Our findings support metabolic dysfunction as an early event in amyotrophic lateral sclerosis pathogenesis. This freely available *SOD1^A5V^* model provides a valuable tool for studying amyotrophic lateral sclerosis progression and identifying therapeutic targets for pre-symptomatic treatment.

## Teaser

Thompson *et al*. report the generation and characterization of novel gene-edited mice expressing the human superoxide dismutase 1 (*SOD1*) gene and carrying the pathogenic *SOD1^A5V^* mutation associated with amyotrophic lateral sclerosis. Integrated multi-omic analyses reveal metabolic dysfunction and establish a freely available model for investigating early pathogenic events and evaluating preclinical therapeutic strategies.

## Graphical Abstract

**Graphical Abstract:**
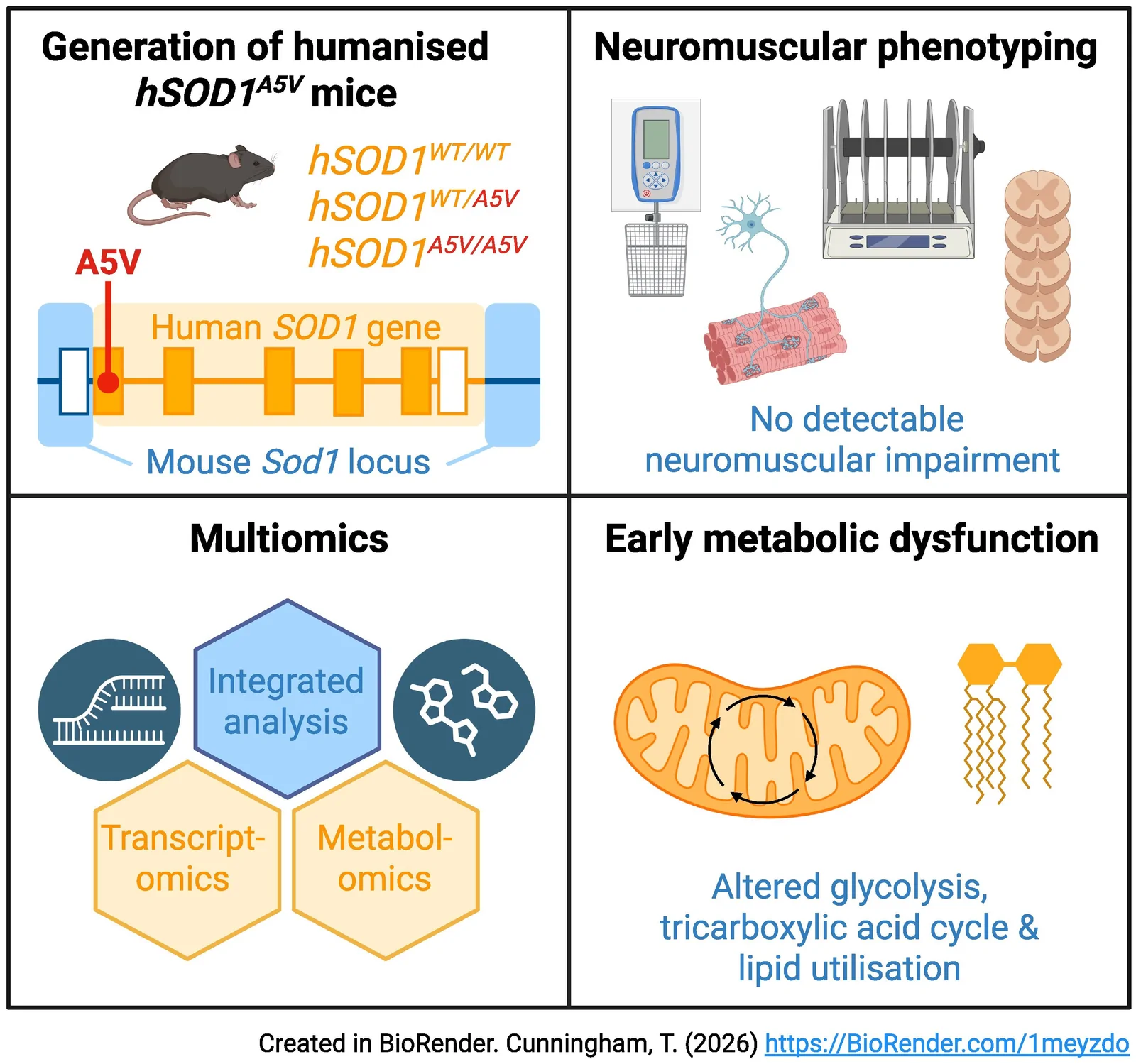

## Introduction

Amyotrophic lateral sclerosis (ALS) is a fatal neurodegenerative disease characterized by the progressive loss of both upper and lower motor neurons, leading to muscle weakness, wasting and progressive paralysis. It is the most common cause of motor neuron disease globally, with an incidence of ∼2/100 000 population and up to a 1/300 lifetime risk of disease.^1,2^ The aetiology of ALS is not understood. The age of onset is around 60 years old for sporadic ALS (sALS), which arises without a known family history, and 40–60 years old for hereditary cases of familial ALS (fALS), with a median survival of 3–5 years from onset.^3^ There are currently no treatments that halt the progression of the disease and a paucity of biomarkers for disease predisposition and pre-symptomatic onset.

ALS likely develops through an interplay of genetic susceptibility and largely unknown environmental factors. Approximately 90% of cases are sALS; the remaining ∼10% are fALS, of which 70% have a mutation identified in an ALS-associated gene, most of which exhibit autosomal dominant inheritance.^4^ Over 50 different ALS-associated genes have been identified, with mutations in *SOD1, FUS, TARDBP* and *C9orf72* causal for 55% of European fALS cases.^5-7^ Thus, ALS is a clinically defined set of diseases with different origins that result in the death of upper and lower motor neurons.^8^

Mutations in *superoxide dismutase 1* (*SOD1*) were the first genetic cause of ALS to be discovered and account for 15–30% of fALS and 2% of sALS cases.^6,9-11^ Disease–causing mutations have been identified in each of the 5 exons of this gene and over 180 different potentially pathogenic variants have been recognized.^12^ Genotype-phenotype associations show that patients carrying A5 V, G85R and G93A mutations typically have rapid disease progression. Other variants, such as P66S and G16S, are associated with an earlier age of onset.^5^ In part due to a founder effect, the A5 V variant located in exon 1 makes up ∼50% of SOD1 pathogenic variants in North America and has an average survival time of <2 years.^4^ Note that A5 V is named using contemporary nomenclature with the mutation lying in the fifth codon position, whereas previously the methionine start codon was not counted and the mutation was referred to as A4 V.

The relative frequency of the A5 V variant accounts for its representation within pre-symptomatic familial ALS research programs especially in North America. Some programs are undertaking a detailed review of genetically predisposed individuals to assess features that may mark the shifts from pre-symptomatic to disease onset, i.e. the transition from a clinically silent period where biomarkers of disease are present to a prodromal phase of non-impactful clinical features (phenotransition).^13^ The conversion of prodromal clinical features into the symptomatic expression of classifiable ALS and the onset of the clinically manifest stages may also be tracked (phenoconversion).^14,15^ However, robust markers of phenotransition and phenoconversion remain to be found and *SOD1*-ALS disease aetiology is unclear.

Genetically modified mouse models have been of great utility for ALS research since the generation of transgenic *SOD1^G93A^* (*tg.SOD1^G93A^*) mice that overexpress mutant human *SOD1* in a background of endogenous mouse *Sod1* expression.^16^  *tg.SOD1^G93A^* mice carry ∼twenty-five transgene copies, and exhibit ∼4-fold higher total SOD1 protein levels in the brain versus non-transgenic mice, and display early onset (∼90 days of age) and fast progression of disease (∼60 days). However, pre-symptomatic or early-stages of pathogenic processes are difficult to assess in such rapidly progressing models, and spurious phenotypes can arise from overexpression *per se*, rather than the effect of the introduced mutation.^9,17^ Transgenic *SOD1^A5V^* (*tg.SOD1^A5V^*) mice, generated in the same study but not widely used, have substantially lower total SOD1 protein levels (only 50% higher than non-transgenic mice) and show no overt phenotype suggesting a threshold level of SOD1 may be required for motor phenotypes to develop in mouse.^16,18^ Without overexpression, typically the onset and progression of disease in mouse models has been less aggressive.^17,19^  *Sod1^D83G^* and *Sod1^G85R^* homozygous mice that express pathogenic mutations in endogenous mouse *Sod1* at physiological levels show mild neurodegenerative phenotypes relative to *tg.SOD1^G93A^* mice. An important distinction with *Sod1^D83G^* and *Sod1^G85R^* mice is that these mutations impart total loss of enzymatic activity, whereas *hSOD1^A5V^* maintains some activity (albeit greatly reduced due to protein instability), suggesting that neurodegenerative phenotypes in physiological SOD1 mouse models may depend on an interaction with loss of function.^9,20,21^

Recently, we developed a genomically humanized *hSOD1^WT/WT^* gene-targeted mouse strain in which the mouse *Sod1* gene was replaced with the wildtype human *SOD1* gene, including all exons and introns, at the endogenous *Sod1* locus.^22^ The human *SOD1* genomic region extends from the ATG start codon to the end of the 3′-UTR; this freely available *hSOD1^WT/WT^* strain retains upstream regulatory sequences including the promoter of mouse *Sod1*, allowing the study of wildtype human SOD1 at physiological expression levels. The *hSOD1^WT/WT^* mouse strain shows no adverse phenotypes over a normal lifespan compared with non-transgenic littermates (Supplementary Figs 1 and 2). Here, we introduced the pathogenic A5 V variant into *hSOD1* mice via CRISPR/Cas9 (clustered regularly interspaced short palindromic repeats/CRISPR-associated protein 9) genome editing. This new strain affords the opportunity to model heterozygous and homozygous effects of a clinically aggressive mutation.

Heterozygous *hSOD1^WT/A5V^* and homozygous *hSOD1^A5V/A5V^* mice have reduced SOD1 protein levels and SOD1 enzyme activity. Over the lifespan of these mice, ∼750 days for males and 850 days for females, *hSOD1^A5V^* animals are indistinguishable from wildtype mice in neuromuscular assessments. However, longitudinal transcriptomic and metabolic profiling identified dysregulation of key pathways in energy metabolism, with glycolysis, the tricarboxylic acid (TCA) cycle, and lipid utilization perturbed in *hSOD1^WT/A5V^* and *hSOD1^A5V/A5V^* animals. This novel humanized model will be useful for examining the factors that promote the transition from an asymptomatic, genetically predisposed state to clinical disease, allowing insight into the clinically silent pre-manifest stage of asymptomatic ALS.^14,15^ This mouse line is freely available from the European Mutant Mouse Archive (EMMA; EM:14880).

## Materials and methods

### Animals

The generation of humanized *hSOD1^WT^* mice has been previously described.^22^ Chimaeric *hSOD1^WT^* founders (derived from R1 129/SvJ embryonic stem cells) were bred for four generations to C57BL/6J (generation N4) before intercrossing heterozygous *hSOD1^WT^/mSod1* (mouse Sod1) animals to produce progeny for phenotyping. To generate humanized *hSOD1^A5V^* mice, the A5 V point mutation (C->T at g.130) was introduced into the humanized *SOD1* allele in *hSOD1^WT^* zygotes via CRISPR/Cas9 editing (conducted following three generations of breeding chimaeric *hSOD1^WT^* founders to C57BL/6J mice). A single-stranded oligodeoxynucleotide (ssODN) donor was used as a repair template. CRISPR/Cas9 system components were delivered as ribonucleoprotein complexes into 1-cell stage *hSOD1^WT^* embryos by electroporation. The sequence of the sgRNA used in the generation of the *hSOD1^A5V^* line was: 5′-GTCGCCCTTCAGCACGCACACGG-3′. The sequence of the ssODN donor was 5′- TTCCTGCGGCGCCTTCCGTCCGTCGGCTTCTCGTCTTGCTCTCTCTGGTCCCTCCGGAGGAGGCCGCCGCGCGTCTCCCGGGGAAGCATGGCGACGAAGG**T**CGTGTGCGTGCTGAAGGGCGACGGCCCAGTGCAGGGCATCATCAATTTCGAGCAGAAGGCAAGGGCTGGGACGGAGGCTTGTTTGCGAGGCCGCTCCCA −3′ (mismatch to introduce C->T point mutation underlined). Electroporated embryos were re-implanted into CD1 pseudo-pregnant females. F_0_ progeny were backcrossed for one further generation into C57BL6/J to obtain germline transmission, and the resulting F_1_ offspring were screened for successful homology-directed repair (HDR) and integration of the A5 V mutation by PCR followed by Sanger sequencing (Table 1). To exclude off-target integration of the donor ssODN, copy counting of the donor sequence was carried out using ddPCR (Table 1). Following confirmation of successful targeting, founder *hSOD1^A5V^* mice (generation N4) were crossed to *hSOD1^WT^* mice to obtain compound heterozygotes (*hSOD1^WT^*/*hSOD1^A5V^*), which were then intercrossed to produce experimental animals. A total of 370 animals were included in this study (215 male animals and 155 female animals).

**Table 1:** PCR primers, sequencing primers, and probes for screening of *hSOD1^A5V^* mice

Assay	Primer/probe	Sequence (5′→ 3′)
PCR across A5 V mutation	Forward primer	GCCGACGTCACAGTTAGAAGAC
	Reverse primer	ACCCTCAGGAACGAGAACAC
Sequencing across A5 V mutation	Sequencing primer	CGGGAACTTTCTCAGTCCGC
	Sequencing primer	CTCAGCACTTGGGCACCG
ddPCR (mutation non-specific)	Forward primer	CGTGTGCGTGCTGAAGG
	Reverse primer	AACAAGCCTCCGTCCCA
	5′ FAM labelled probe	CAATTTCGAGCAGAAGGCAAGGGC
ddPCR (specific for *hSOD1^A5V^* allele)	Forward primer	TTCCGTCCGTCGGCTTC
	Reverse primer	CTTCAGCACGCACACGA
	5′ FAM labelled probe	TCGTCTTGCTCTCTCTGGTCCCTC

Mice were housed in standard cages of up to 5 animals, with *ad libitum* access to food and water, a 12h:12 h light cycle with lights-on at 7 a.m., and environmental temperature and humidity at 21 ± 2°C and 55 ± 10%, respectively. Cage allocation was randomly assigned with respect to genotype and litter. All animal husbandry and experiments were performed at MRC Harwell Institute and were in accordance with the Animals (Scientific Procedures) Act 1986, Home Office guidelines and the local ethics guidelines issued by the Medical Research Council. All reasonable measures were taken to minimize animal distress and suffering.

#### Genotyping

Animal genotyping was performed on genomic DNA extracted from ear clip biopsies using loss-of-allele copy number quantitative PCR (qPCR) with primers and probes listed in Table 2.

**Table 2:** Genotyping primers and probes

Allele	Forward primer 5′ -> 3′	Reverse primer 5′ -> 3′	5′-FAM labelled probe 5′ -> 3′
*hSOD1^WT^*	GTGCAGGTCCTCACTTTAATCC	CCAGAAAGCTATCGCCATTATTACAAG	CCAAAGGATGAAGAGAGGTAACAAGATGC
*hSOD1^A5V^*	TTCTCGTCTTGCTCTCTCTG	CCTTCTGCTCGAAATTGATGATG	ACGCACACGACCTTCG

#### Experimental groups

Longitudinal behavioural, motor, and metabolic phenotyping were performed at regular intervals on both male and female animals from two independent cohorts. Data from the two cohorts were pooled. Tissues were harvested from these two cohorts for histology and molecular phenotyping at 24 months old. Tissues were harvested from additional cohorts for histology and molecular phenotyping at earlier timepoints. In all experiments, *hSOD1^A5V^* mice were compared with *hSOD1^WT/WT^* littermate controls, which have a genomically humanized *SOD1* locus but lack the A5 V mutation. Group size was determined based on prior experience and practical considerations.

#### Harvesting of tissues

Animals were terminally anesthetized with 1000 mg/kg of pentobarbitone administered via intraperitoneal injection. Once sufficiently anesthetized all, or a subset, of the following tissues were harvested from each animal: brain, spinal cord (SC), kidney, liver and quadriceps, tibialis anterior (TA), extensor digitorum longus (EDL), soleus, lumbrical muscles, and tail. Further processing of fresh tissue differed depending on the experiment.

### Motor phenotyping

All phenotyping was performed in the light phase (7 a.m.–7 p.m.). For all tests, mice were acclimatized to the testing room for at least 30 min prior to starting the procedure.

At each time point (6-, 12-, 18- and 24-months-old) motor and behavioural phenotyping tests were performed in the following order: open field (data not shown), combined SHIRPA^23^ and dysmorphology (data not shown), gait analysis (Supplementary Methods), Locotronic (data not shown), Rotarod (Supplementary Methods), grip strength (Supplementary Methods), automated wheel running (data not shown). Within each experiment, the order of animals was not explicitly randomized, but genotypes were interspersed via housing in randomly assigned mixed-genotype cages. The experimenter was blinded to genotype during experiments.

### Metabolic phenotyping

EchoMRIs, Intra-peritoneal glucose tolerance testing (IPGTT), monthly weight and survival analyses were performed to metabolically phenotype *hSOD1^WT/WT^* and *hSOD1^A5V^* animals (Supplementary Methods). Metabolomic profiles of *hSOD1^WT/WT^* and *hSOD1^A5V^* animals were generated via analysis of blood glutathione measurements and liquid and gas chromatography mass spectrometry (LC-MS and GC-MS) experiments on plasma and SC, TA and liver tissue of *hSOD1^A5V^* animals (Supplementary Methods).

### Histology, immunohistochemistry and immunofluorescence

Motor neuron (MN) counts and neuromuscular junction (NMJ) analysis in the lumbrical, TA and soleus muscles were performed in *hSOD1^WT/WT^* and *hSOD1^A5V^* animals (Supplementary Methods). Muscle fibre typing, sizing and central nucleation analysis was performed in the TA and the axonal transport of signalling endosomes was studied in TA-innervating motor axons (Supplementary Methods).

### Biochemical and molecular analyses

#### RNA analysis

For RNA analysis, tissues were immediately immersed in ice-cold RNAlater solution (Sigma Aldrich) and stored at −20°C in RNAlater until use. RNA was extracted from CNS tissue using RNeasy Lipid Tissue Mini Kit (Qiagen), from muscle using an RNeasy Fibrous Tissue Mini Kit (Qiagen), and from kidney and liver using an RNeasy Mini Kit (Qiagen) (Supplementary Methods). Complementary DNA (cDNA) was generated using a High-Capacity cDNA Reverse Transcription Kit (Applied Biosystems) (Supplementary Methods). The sequences of primers used in this study are listed in Table 3. Relative quantification of gene expression was performed using the 2^−ΔΔ*CT*^ method. *Gapdh* was used as a housekeeping gene for normalization of *SOD1* gene expression.

**Table 3:** List of RT-qPCR primers

Primer Name	Primer sequence (5′->3′)
*SOD1* forward primer	TGAAGAGAGGCATGTTGGAGA
*SOD1* reverse primer	CCACCTTTGCCCAAGTCATC
*Gapdh* forward primer	CGGCCGCATCTTCTTGTG
*Gapdh* reverse primer	CCGACCTTCACCATTTTGTCTAC

#### Protein analysis

For protein analysis, tissues were flash-frozen in liquid nitrogen and stored at −80°C until use. Western blotting was performed using a pan-SOD1 antibody raised against a peptide corresponding to amino acids 131–153 of the SOD1 protein, which are identical in human and mouse SOD1 (Supplementary Methods). In-gel dismutase activity assays were performed according to Weydert & Cullen^24^ (Supplementary Methods). The protocol for a high throughput spectrophotometric SOD dismutase activity assay was adapted from Peskin and Winterbourn^25^ (Supplementary Methods). The enzyme-linked immunosorbent assay (ELISA) protocol for soluble disordered SOD1 (misELISA) was adapted from Zetterström *et al*.^26^ using an anti-SOD1 human monoclonal antibody raised against amino acids 24–39 (exposed only in disordered SOD1) (Supplementary Methods).

### Transcriptomics

#### Quantseq 3′ mRNA sequencing

We performed QuantSeq 3′ mRNA sequencing (Lexogen) in the TA muscle and lumbar SC of 6-, 12- and 20-month-old male animals (a single sex, male, was used for transcriptomics to minimize cost and data variability) comparing *hSOD1^WT/A5V^* and *hSOD1^A5V/A5V^* animals versus *hSOD1^WT/WT^* littermate controls (*n* = 4 animals per genotype and time point). Library preparation and Illumina sequencing were performed by Lexogen. cDNA libraries were prepared using QuantSeq 3′ mRNA-Seq Library Prep FWD kits (Lexogen) and sequenced on an Illumina platform to generate single-end reads. Raw reads are provided in the file Supplementary Data 6. Basic statistics are listed in Table 4.

**Table 4:** Read counts from quantseq 3′ mRNA sequencing runs

Sample group	Total read count (median)	Uniquely mapped reads (median)	Uniquely mapped reads % (median)	Average input read length (median)
6-month-old spinal cord	8200155	5369403	65.7	86
6-month-old TA muscle	7379234	4984674	68.0	90
12-month-old spinal cord	4860088	3198704	66.2	66
12-month-old TA muscle	4767525	3146542	65.7	67
20-month-old spinal cord	5150797	3571121	69.0	86
20-month-old TA muscle	4609703	3419747	74.4	88

### Data analysis

#### Transcriptomics data analysis

Transcriptomic data was analysed using packages including Trimmomatics, HISAT2, FeatureCounts and DESeq2 (Supplementary Methods). The Benjamini and Hochberg procedure was implemented for correction of *P-*values with false discovery rate (FDR) to identify significant differentially expressed genes (DEGs). To identify genes considered possibly biologically relevant, analysis was also conducted using a *P* < 0.01.

#### Metabolomics data analysis

Data pre-processing and annotation of metabolites was performed by the Swedish Metabolomics Centre and provided in the file Supplementary Data 7. The input for statistical analyses was a data matrix of annotated compound names with associated peak (mz/rt) intensities. Univariate analyses were performed to identify significant differences between the levels of metabolites in *hSOD1^WT/WT^*, *hSOD1^WT/A5V^*, and *hSOD1^A5V/A5V^* animals (*P* < 0.05).

#### Integrated transcriptomic and metabolomic analysis

Integrated network analysis and visualization of transcriptomic and metabolic data were performed using a combination of STRING (Search Tool for the Retrieval of Interacting Genes/Proteins), Reactome databases, and BioRender.

#### Statistical analyses

Statistical analyses were performed in GraphPad Prism (Dotmatics). All data used to produced main and supplemental figures are provided in the file Supplementary Data 9. Statistical significance for phenotyping and molecular experiments was determined using ANOVA (one-way, two-way, and two-way with repeated measures) or mixed effects analysis, using a significance threshold of *P* ≤ 0.05. Statistical analysis of RNA-seq and metabolomics experiments are described in detail in the relevant methods sections. The researcher was not blinded to genotype during data analysis. A retrospective power analysis is provided in the file Supplementary Data 8. The statistical results for each analysis are shown in the relevant figure legends.

## Results

### Generation and initial characterization of genomically humanized *SOD1^A5V^* mice

**Figure 1:**
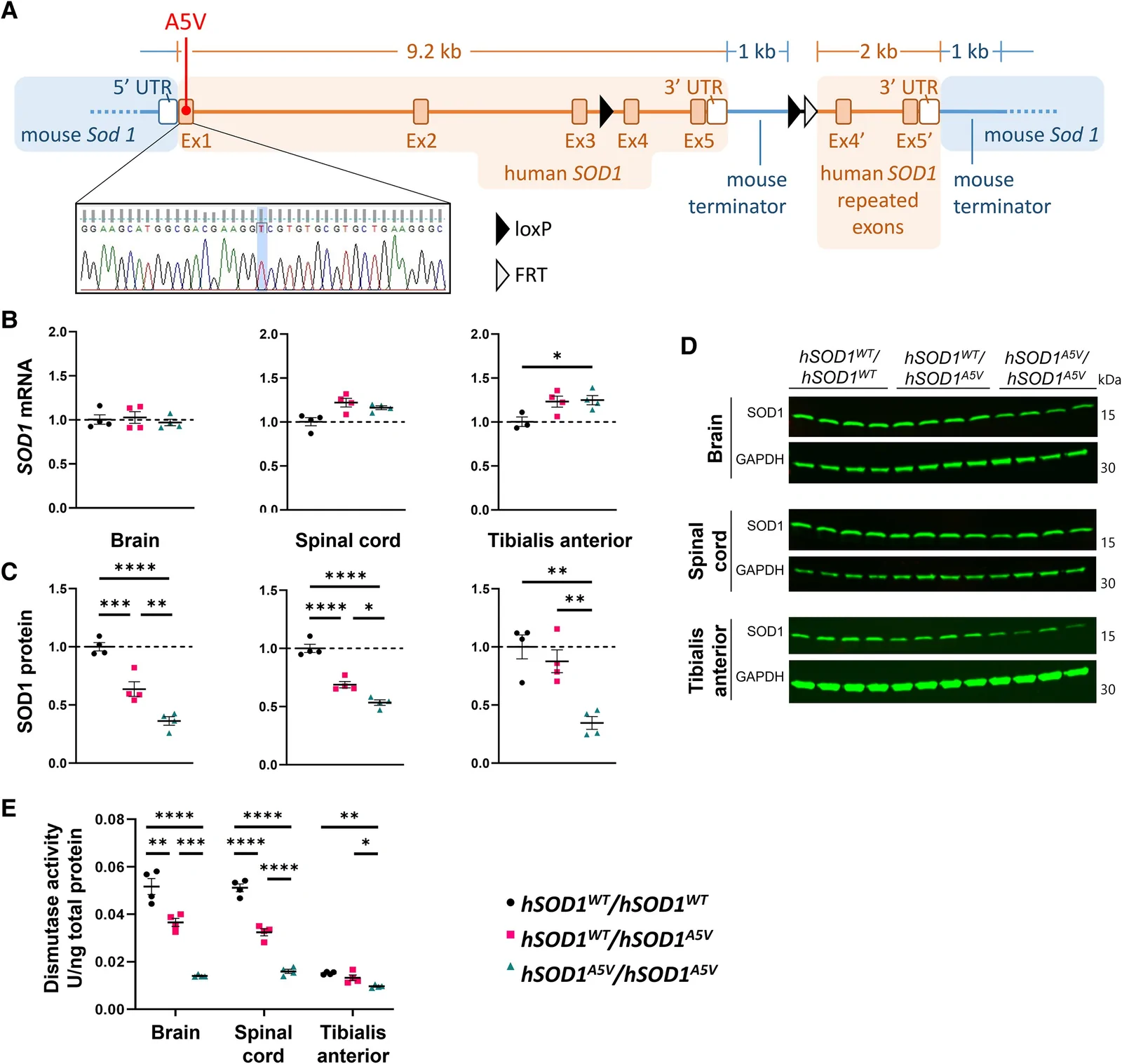
**SOD1 expression and activity in *hSOD1^A5V^* mice.** (**A**) Schematic of the *hSOD1^A5V^* allele at the endogenous mouse *Sod1* locus. Exons (Ex), intervening introns and the 3' untranslated region (UTR), including a duplication of exons 4-5, comprise human sequence inserted at the mouse *Sod1* locus, replacing the orthologous mouse sequence. The 5' UTR and the downstream mouse terminator comprise endogenous mouse regions. Inset: Sequencing trace from a homozygous *hSOD1^A5V^* animal showing the A5 V point mutation (C->T; highlighted). (**B**) Dot plot showing relative expression of total *SOD1* mRNA using conserved mouse-human *SOD1/Sod1* primers in tissues from 3-month-old male animals (*n* = 3–4 animals per genotype per tissue). All values are expressed as fold change relative to the average *SOD1* expression in *hSOD1^WT/WT^* control animals. Data normalized to *Gapdh.* (**C**) Dot plot showing expression of total SOD1 in tissues from 3-month-old male animals (*n* = 4 animals per genotype per tissue). All values are expressed as fold change relative to the average *SOD1* expression in *hSOD1^WT/WT^* control animals. Anti-SOD1 signal intensity was normalized to anti-GAPDH signal intensity in the same lane. (**D**) Western blots using an anti-SOD1 antibody (aa131–153, reacts with mouse and human) in tissue extracts from 3-month-old male animals (*n* = 4 animals per genotype per tissue). Human SOD1 migrates with a higher apparent molecular mass than mouse SOD1 in SDS-PAGE (sodium dodecyl sulfate-polyacrylamide gel electrophoresis). (**E**) Dot plot showing spectrophotometric dismutase activity assays in tissues from 3-month-old male *hSOD1^A5V^* mice (*n* = 4 animals per genotype per tissue). All panels: one-way ANOVA with Tukey’s *post hoc* test. Each datapoint represents an individual animal. Reported significance values in all panels are from Tukey’s test. Main effects: (**B**) brain *F*(2,9) = 0.285, *P* = 0.759; spinal cord *F*(2,9) = 7.60, *P* = 0.0117; tibialis anterior *F*(2,9) = 1.65, *P* = 0.245; (**C**) brain *F*(2,9) = 46.8, *P* = 1.76 × 10^−5^; spinal cord *F*(2,9) = 65.5, *P* = 4.34 × 10^−6^; tibialis anterior *F*(2,9) = 15.6, *P* = 0.00120; (**D**) brain *F*(2,9) = 74.4, *P* = 2.52 × 10^−6^; spinal cord *F*(2,9) = 162, *P* = 8.87 × 10^−8^; tibialis anterior *F*(2,9) = 14.3, *P* = 0.00159. Mean ± SEM displayed in all panels. *P < 0.05, **P < 0.01, ***P < 0.001, ****P < 0.0001. Relates to Supplementary Figs 3–6. Full uncropped blots are shown in Supplementary Fig. 28.

Using CRISPR/Cas9 mutagenesis, we edited our previously published *hSOD1^WT/WT^* mouse strain^22^ to generate SOD1-A5 V (*hSOD1^A5V^*) mice (Fig. 1A). Note that the A5 V mutation was placed into our existing *hSOD1^WT^* allele, in which we had created a duplication of the exon 4–5 region, where several important SOD1 variants occur; in the future, this will allow the effects of mutations in these two exons to be temporally and spatially analysed, using Cre recombinase. However, as the A5 V mutation is in exon 1, we did not use this feature here.

We analysed animals homozygous for the wildtype humanized locus (*hSOD1^WT/WT^*) as controls, compound heterozygotes carrying the wildtype humanized locus and the humanized A5 V allele (*hSOD1^WT/A5V^*), or homozygous for the humanized A5 V allele (*hSOD1^A5V/A5V)^* (herein, the use of *hSOD1^A5V^* denotes heterozygote and homozygote A5 V animals). To determine the effect of A5 V mutation on *SOD1* mRNA expression, we performed RT-qPCR in a range of tissues (brain, spinal cord (SC), quadriceps, tibialis anterior (TA), extensor digitorum longus (EDL), soleus, kidney, and liver) from 3-month-old male and female *hSOD1^A5V^* animals and *hSOD1^WT/WT^* littermate controls. *SOD1* mRNA expression did not differ significantly between *hSOD1^A5V^* animals and *hSOD1^WT/WT^* littermate controls in the majority of tissues examined in male (Fig. 1B and Supplementary Fig. 3A) and female animals (Supplementary Fig. 3B) although *SOD1* mRNA expression was increased in the TA muscle of male *hSOD1^A5V/A5V^* mice (Fig. 1B) and the liver of male *hSOD1^WT/A5V^* mice (Supplementary Fig. 3A), compared with *hSOD1^WT/WT^* (*n* = 4 animals per genotype per sex per tissue).

In contrast to mRNA levels, *hSOD1^A5V^* animals had significantly reduced SOD1 protein levels in almost all tissues examined in both sexes (Fig. 1C and D; Supplementary Figs 4 and 5). SOD1 protein levels correlated with the zygosity of the A5 V mutation, with reduced SOD1 detected in *hSOD1^A5V/A5V^* versus *hSOD1^WT/A5V^* animals. Protein expression in *hSOD1^A5V/A5V^* animals ranged from half to approximately one tenth of the levels found in tissues from *hSOD1^WT/WT^* littermate controls with *hSOD1^WT/A5V^* showing intermediate levels.

SOD1 activity in homozygous *hSOD1^A5V/A5V^* tissues was also significantly reduced in all tissues examined from 3-month-old male and female animals, exhibiting between 10% and 40% of the activity observed in the corresponding tissue from *hSOD1^WT/WT^* littermates (Fig. 1E and Supplementary Fig. 6).

**Figure 2:**
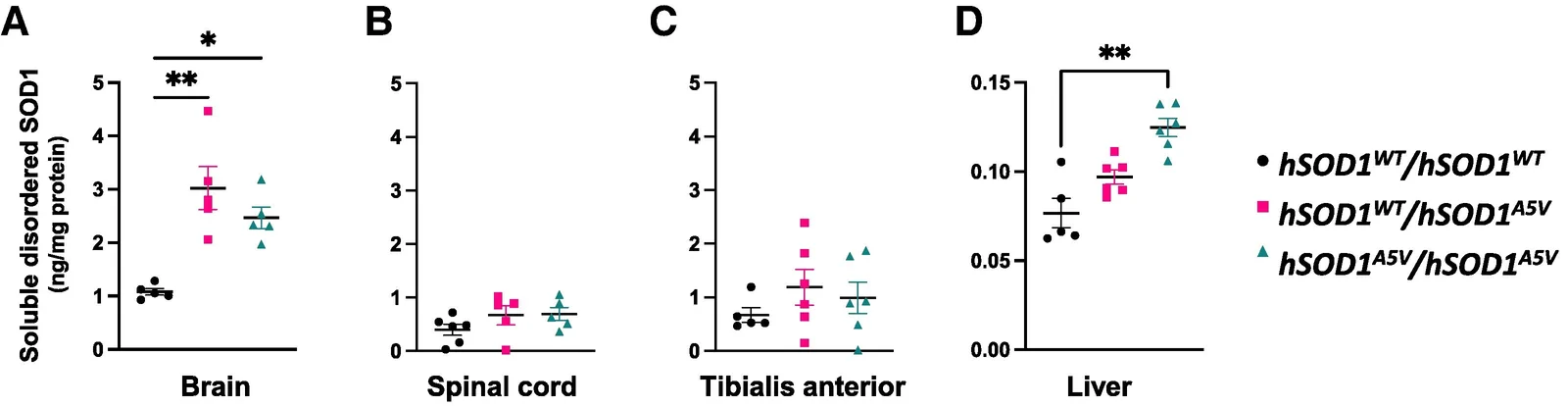
**Disordered SOD1 in *hSOD1^A5V^* mice.** Levels of soluble disordered SOD1 in supernatants of (**A**) brain, (**B**) spinal cord, (**C**) tibialis anterior and (**D**) liver of 12-month-old male animals (*n* = 5–6 animals per genotype per tissue), quantified by ELISA (enzyme-linked immunosorbent assay) performed with a peptide antibody against amino acids 24–39 recognizing an epitope exposed in disordered SOD1. All panels: one-way ANOVA with Tukey’s *post hoc* test. Each datapoint represents an individual animal. Reported significance values in all panels are from Tukey’s test. Main effects: (**A**) brain *F*(2,12) = 14.67, *P* = 0.0006; (**B**) spinal cord *F*(2,14) = 0.8229, *P* = 0.4593; (**C**) tibialis anterior *F*(2,13) = 1.515, *P* = 0.2562; (**D**) liver *F*(2,14) = 17.02, *P* = 0.0002. Mean ± SEM displayed in all panels. *P < 0.05, **P < 0.01.

To determine the effect of the A5 V mutation on SOD1 stability, we assessed levels of soluble disordered SOD1 via ELISA in tissues from male 12-month-old *hSOD1^A5V^* and *hSOD1^WT/WT^* littermates (Fig. 2A–D). The highest levels were detected in brain and were significantly increased in brain from *hSOD1^WT/A5V^* and in brain and liver from *hSOD1^A5V/A5V^* animals (Fig. 2A and D).

### *hSOD1^A5V^* mice lack an overt neurodegeneration phenotype

We performed extensive motor and behavioural phenotyping of *hSOD1^A5V^* animals. Onset of progressive motor dysfunction is the hallmark of human ALS and is shown in overexpression transgenic ALS mouse models by deficits in grip strength, Rotarod and gait.^27-32^ However, we did not observe deficits in these tests in our knock-in *hSOD1^A5V^* mice (Supplementary Fig. 7), nor did we detect atrophy of the TA muscle (Supplementary Fig. 8). We also did not detect NMJ denervation in the TA, soleus or lumbrical muscles of *hSOD1^A5V^* animals (Supplementary Fig. 9A–D), nor loss of motor neurons in the lumbar L3-L5 region of the SC, suggesting that the *SOD1-A5 V* mutation does not impact motor neuron survival in *hSOD1^A5V^* mice at the ages studied (Supplementary Fig. 9E). Furthermore, in contrast to *tg.SOD1^G93A^* mice,^33^ we did not observe *in vivo* axonal transport deficits in TA-innervating motor axons (Supplementary Fig. 10).

### *hSOD1^A5V^* mice have mild metabolic phenotypes

**Figure 3:**
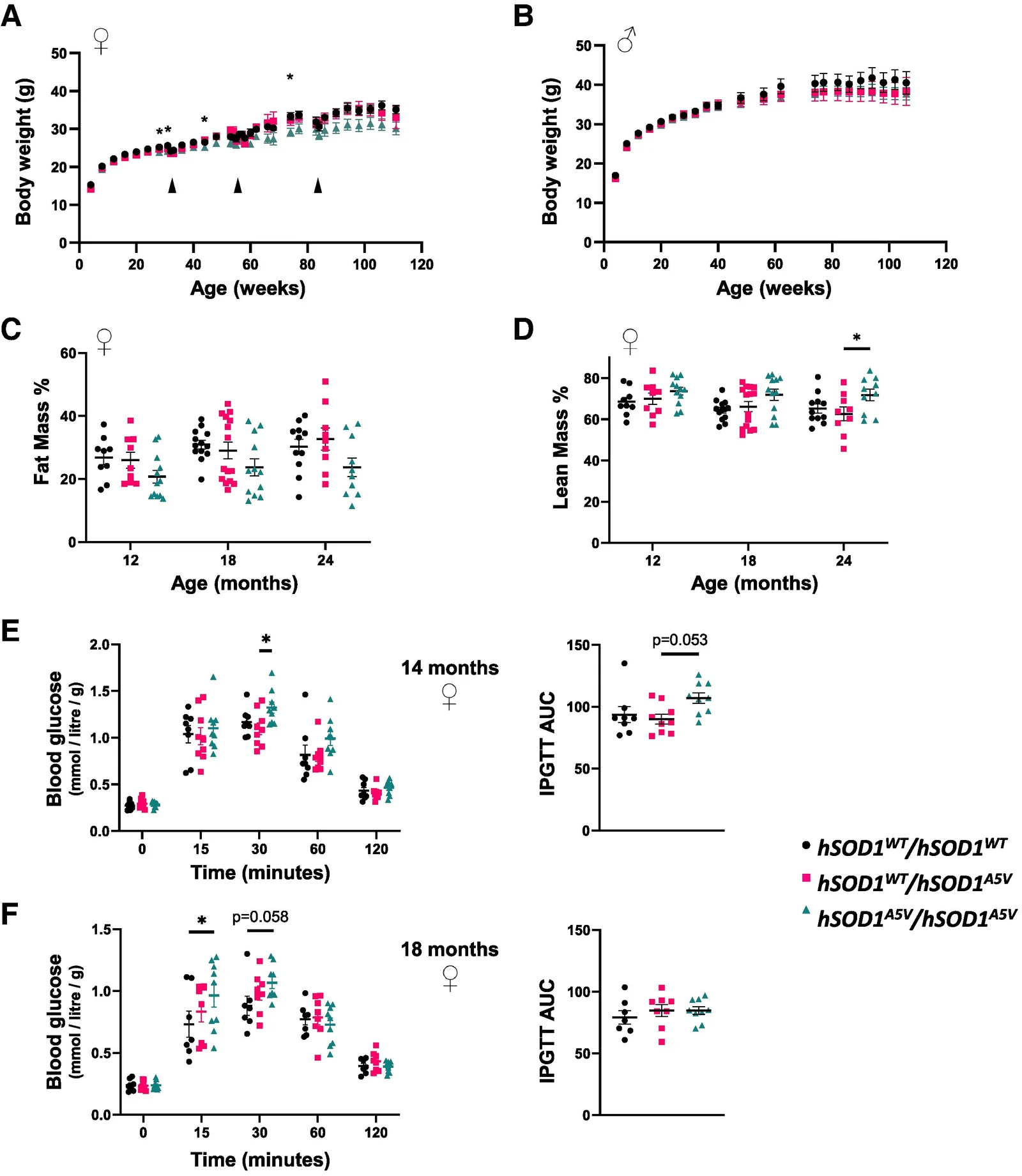
***hSOD1^A5V^* mice have a mild metabolic phenotype.** (**A, B**) Monthly bodyweight measurement data from female (*n* = 14–16 per genotype at 4 weeks old, *n* = 6–9 per genotype at 111 weeks old) and male (*n* = 12–16 per genotype at 4 weeks old, *n* = 8–12 per genotype at 106 weeks old) *hSOD1^A5V^* mice. Arrowheads indicate ages where automated wheel running experiments were performed, showing expected dips in bodyweight. Each datapoint represents the average bodyweight among all animals of the same genotype. Mixed effects analysis followed by the Tukey multiple comparisons test. Genotype effect: (**A**) *F*(2,43) = 4.19, *P* = 0.0217; (**B**) *F*(2,39) = 0.291, *P* = 0.749 (**C**, **D**) EchoMRI data showing fat mass and lean mass as a percentage of total bodyweight in female (*n* = 9–15 per genotype) *hSOD1^A5V^* animals. Two-way ANOVA with Tukey’s *post hoc* test. Genotype effect: (**C**) *F*(2,91) = 6.80, *P* = 0.00176; (**D**) *F*(2,91) = 6.73, *P* = 0.00188 (**E**, **F**) Intraperitoneal glucose tolerance testing (IPGTT) data from (**E**) 14-month-old (*n* = 8–9 per genotype) and (**F**) 18-month-old (*n* = 7–9 per genotype) female *hSOD1^A5V^* mice. Changes in blood glucose over time were analysed using two-way repeated measures ANOVA followed by Tukey’s test. Genotype effect: (**E**) *F*(2,23) = 2.89, *P* = 0.0761; (**F**) *F*(2,21) = 1.02, *P* = 0.379. Area under the curve (AUC) for each graph was analysed using one-way ANOVA followed by Tukey’s test. Genotype effect: (**E**) *F*(2,23) = 3.40, *P* = 0.0510; (**F**) *F*(2,21) = 0.488, *P* = 0.621. (C–F) Each datapoint represents a single animal. Reported significance values in all panels are from Tukey’s test. Mean ± SEM displayed in all panels. *P < 0.05.

Metabolic changes have been widely observed in ALS mouse models, including at pre-symptomatic stage.^34-38^; hence, we sought to establish if metabolism is perturbed in *hSOD1^A5V^* mice. To assess the impact of the A5 V mutation on bodyweight, *hSOD1^A5V^* animals were weighed monthly from 1 to 26 months of age (Fig. 3A and B). There was a significant effect of age (*P* < 0.001) and genotype (*P* = 0.022), and a significant interaction between age and genotype (*P* = 0.003) on bodyweight in female *hSOD1^A5V^* animals (Fig. 3A). Female *hSOD1^A5V/A5V^* mice gained weight more slowly than controls after 6 months of age, suggesting this difference was not due to impaired development. No difference in bodyweight was observed in male animals (Fig. 3B).

To examine whether reduced bodyweight in female *hSOD1^A5V^* animals could be attributed to changes in fat or lean mass we performed EchoMRI on 12- 18-, and 24-month-old animals (Fig. 3C and D). 2-way ANOVA revealed a significant effect of genotype, but not age, on percentage fat mass (*P* = 0.002) and percentage lean mass (*P* = 0.002) in female *hSOD1^A5V^* animals. Pairwise comparisons conducted at each age found a significant increase in percentage lean mass in 24-month-old *hSOD1^A5V/A5V^* compared with *hSOD1^WT/A5V^* females. However, across all ages there was only a general trend towards decreased percentage fat mass and increased percentage lean mass in female homozygous *hSOD1^A5V/A5V^* mice compared with the other groups (age and sex matched).

Glucose metabolism was investigated in 14- and 18-month-old female *hSOD1^A5V/A5V^* mice using the intraperitoneal glucose tolerance test (IPGTT; Fig. 3E and F). Multiple pairwise comparisons were conducted at each sampling time. A significant increase in blood glucose was detected 15 and 30 min after glucose infusion in 14- and 18-month-old female *hSOD1^A5V/A5V^* animals, respectively, compared with *hSOD1^WT/WT^* littermate controls, suggesting an impaired response to glucose.

### Transcriptomic changes at different timepoints in *hSOD1^A5V^* mice

We performed QuantSeq 3′ mRNA sequencing (Lexogen) in the TA muscle and lumbar SC of 6-, 12- and 20-month-old male animals and assessed gene expression differences in *hSOD1^WT/A5V^* and *hSOD1^A5V/A5V^* animals versus *hSOD1^WT/WT^* littermate controls. Average gene expression was obtained (*n* = 4 animals per genotype and time point) and the proportion of differentially expressed genes (DEGs) between groups was initially determined using a false discovery rate adjusted *P*-value of <0.1 as the cut-off. A small number of significant DEGs were identified (Supplementary Data 1) in the TA muscle of *hSOD1^WT/A5V^* and *hSOD1^A5V/A5V^* animals at 6 and 20 months of age and in the lumbar SC of *hSOD1^WT/A5V^* and *hSOD1^A5V/A5V^* at 20 months of age, compared with *hSOD1^WT/WT^* littermate controls (Supplementary Data 1, Supplementary Figs 11 and 12). To widen the search in order to identify biological pathways, analysis was then conducted using a *P-*value < 0.01 (Supplementary Figs 11 and 12). There was minimal overlap of DEGs between genotypes and time points in within-tissue comparisons in the TA and SC (Supplementary Fig. 13, Supplementary Data 2).

DEGs with the largest fold change (10 most upregulated and 10 most downregulated genes) were identified in each data set (Supplementary Data 3, for a complete list of DEGs see Supplementary Data 4 and 5). A large proportion of these genes was predicted or unannotated (26% of genes for TA, 50% of genes for SC). Of the annotated genes, several have been previously linked to ALS (Supplementary Data 3).

To characterize the functional relevance of the observed transcriptional changes, the enrichment analysis tool Enrichr^39-41^ was used to identify gene ontology (GO) terms and infer canonical pathways regulated by DEGs.

GO enrichment analysis across DEGs at 6-, 12- and 20-months in the TA of male *hSOD1^WT/A5V^* and *hSOD1^A5V/A5V^* animals, compared with *hSOD1^WT/WT^* littermate controls, revealed altered expression of genes involved in: aerobic electron transport chain activity; mitochondrial function; malate and nitric oxide metabolism; ribosomal function; and stress granule assembly; among others (Supplementary Figs 14 and 15). GO enrichment analysis across DEGs at 6-, 12- and 20-months in the SC of male *hSOD1^WT/A5V^* and *hSOD1^A5V/A5V^* animals, compared with *hSOD1^WT/WT^* littermate controls, revealed GO terms related to: oxidative stress-induced cell death; ATPase complex activity; oxidoreductase activity; and malate and pyruvate metabolism; among others (Supplementary Figs 16 and 17). DEGs involved in aerobic respiration and translation were detected in the TA and SC of *hSOD1^WT/A5V^* and *hSOD1^A5V/A5V^* animals, suggesting that despite few overlaps between DEGs, the pathways that are affected are common upon *SOD1^A5V^* mutation.

**Figure 4:**
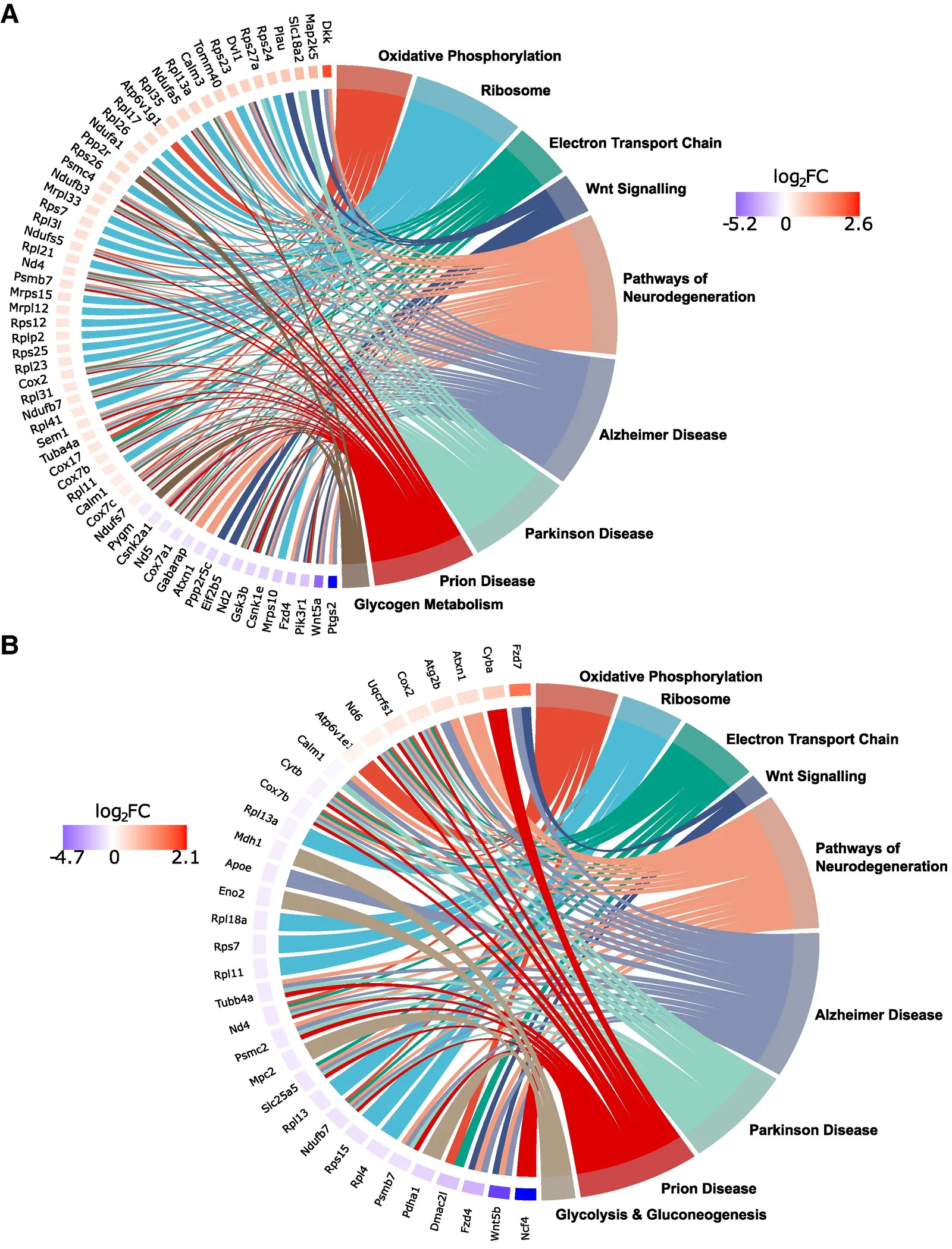
**Chord diagram showing the core genes of significantly affected pathways in *hSOD1^WT/A5V^* mice compared with *hSOD1^WT/WT^* littermate controls.** Significant pathways identified from DEGs (differentially expressed genes) in (**A**) TA (tibialis anterior muscle) and (**B**) SC (spinal cord) of *hSOD1^WT/A5V^* versus *hSOD1^WT/WT^* 6-, 12- and 20-month-old male mice (*n* = 4 per genotype per timepoint per tissue). Significant pathways are shown on the right and the fold change of core genes is shown on the left. Left-right connections indicate gene membership in a pathway’s leading-edge subset. Created in BioRender. Price, G. (2026) https://BioRender.com/okyhdza. Relates to Supplementary Figs 11–18.

The presence of commonly altered pathways is further shown in the enriched KEGG (Kyoto Encyclopedia of Genes and Genomes) pathways. Of the 10 most significant identified KEGG pathways, eight are represented in both the TA and SC of male *hSOD1^WT/A5V^* animals when compared with *hSOD1^WT/WT^* littermate controls (Fig. 4A and B). These pathways include oxidative phosphorylation, ribosome, electron transport chain, and *Wnt* signalling. Several KEGG pathways related to neurodegenerative disease were also identified in the *hSOD1^WT/A5V^* TA and SC, including the terms: pathways of neurodegeneration; Alzheimer’s disease; Parkinson’s disease; prion disease. Additional altered KEGG pathways included: energy metabolism including glycogen metabolism and glycolysis/gluconeogenesis, which were identified in the TA and SC, respectively.

Enrichment analysis identified few significantly altered KEGG pathways in *hSOD1^A5V/A5V^* tissues; however, pyruvate metabolism was enriched in both the TA and SC of *hSOD1^A5V/A5V^* mice (Supplementary Fig. 18).

Transcriptomic analysis of changes in the TA and SC of *hSOD1^WT/A5V^* and *hSOD1^A5V/A5V^* animals compared with *hSOD1^WT^*^/*WT*^ littermate controls highlighted several pathways related to neurodegenerative disease and significant changes in metabolism, encompassing genes related to glucose and pyruvate metabolism, the electron transport chain and ATPase complex activity.

### Metabolic changes over life in *hSOD1^A5V^* mice

To investigate potential metabolomic changes indicated from metabolic phenotyping and transcriptomic analyses, we performed liquid and gas mass spectroscopy on plasma from 4-, 6-, 9-, 13- and 20-month-old *hSOD1^A5V^* male and female mice (Supplementary Figs 19–21) and tissue samples (SC, TA, liver) from 20-month-old *hSOD1^A5V^* male mice (Supplementary Figs 22 and 23).

Univariate analysis identified a total of 3, 15, 1 and 8 significantly altered metabolites in plasma collected at 4-, 6-, 9- and 20-months of age, respectively, in *hSOD1^WT/A5V^* mice compared with *hSOD1^WT/WT^* littermate controls (Supplementary Figs 19–21). *hSOD1^WT/A5V^* animals had increased levels of hydroxy fatty acids, lysophospholipids, acylcarnitines, and amino- and keto-acids, and reduced levels of glucose and the NAD precursor niacinamide. Interestingly, threonic acid (also known as dehydroascorbic acid) was significantly decreased in the blood of 6-month-old *hSOD1^WT/A5V^* mice compared with *hSOD1^WT/WT^* littermate controls. Threonic acid is produced upon oxidation of ascorbic acid, a key antioxidant with an established protective role against oxidative stress in the CNS.^42^

Univariate analysis identified a total of 2, 6, 3 and 9 significantly altered metabolites in plasma collected at 6-, 9-, 13- and 20-months of age, respectively, in *hSOD1^A5V/A5V^* mice compared with *hSOD1^WT/WT^* littermate controls (Supplementary Figs 19–21). *hSOD1^A5V/A5V^* animals had increased levels of amino acids and their derivatives, increased lysophospholipids, and reduced levels of fatty acids and sterols. Pyruvic acid, a central metabolite in glycolysis, was increased. The beta-hydroxybutyrate ester (3-hydroxybutyric acid), currently under trial for treatment of ALS patients,^43^ was significantly decreased.

Univariate analysis identified a total of 0, 4 and 4 significantly altered metabolites in SC, TA and liver, respectively, in male 20-month-old *hSOD1^WT/A5V^* mice compared with *hSOD1^WT/WT^* littermate controls (Supplementary Figs 22 and 23). In TA, the nucleoside guanosine and in the dinucleotide NAD were decreased (Supplementary Fig. 22B). In liver, the TCA cycle intermediate succinic acid was increased, as was the pentose phosphate pathway intermediate ribose-5-phosphate (Supplementary Fig. 23).

Univariate analysis identified a total of 2, 2, and 9 significantly altered metabolites in SC, TA and liver, respectively, in male *hSOD1^A5V/A5V^* mice compared with *hSOD1^WT/WT^* littermate controls (Supplementary Figs 22 and 23). In SC, the lysophospholipid lysoPE (0:0/18:3(6Z, 9Z, 11Z)) was decreased whereas hippuric acid was increased (Supplementary Fig. 22A). Sorbose, a keto-hexose sugar necessary for vitamin C biosynthesis, was significantly increased (Supplementary Fig. 22B). In liver, several amino acid and glycine conjugates were significantly increased, and ribose-5-phosphate was significantly increased, in *hSOD1^A5V/A5V^* mice compared with *hSOD1^WT/WT^* littermate controls (Supplementary Fig. 23).

Reduced glutathione (GSH) reacts with reactive oxygen species and is converted to its oxidized form (GSSG) in the process—forming a key part of the antioxidant defence.^44^ A disruption in SOD1 function has been shown to perturb other antioxidant systems, hence we examined the levels of GSH and GSSG in blood from 4- and 20-month-old *hSOD1^A5V^* mice.^45,46^ Levels of GSH were unchanged at both timepoints (Supplementary Fig. 24). However, we found a significant decrease in GSSG in 13- and 20-month-old *hSOD1^A5V/A5V^* animals compared with controls. Accordingly, we observed an increase in the ratio of GSH-to-GSSG in 20-month-old *hSOD1^A5V/A5V^* animals.

### Integrated transcriptomic and metabolomic analysis identifies changes in energy metabolism

**Figure 5:**
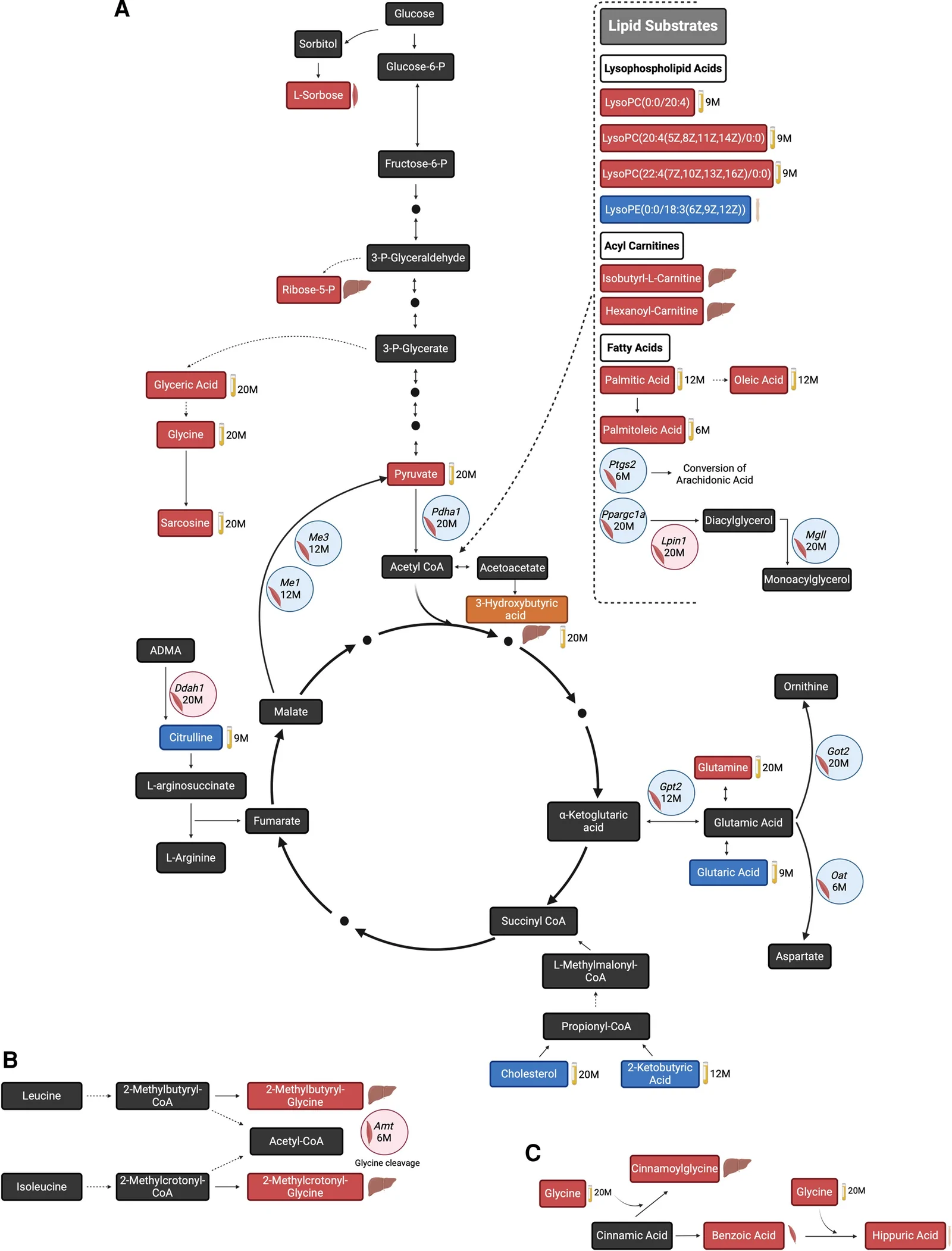
**Integrated transcriptomic and metabolomic analysis of *hSOD1^A5V/A5V^* mice.** Schematic representation of dysregulated pathways, (**A**) energy metabolism pathways, (**B**) leucine and isoleucine degradation, (**C**) aromatic acid detoxification in *hSOD1^A5V/A5V^* mice. Metabolites are depicted in rectangles, red: increased, blue: decreased and black: undetected. Genes are depicted in circles, red: increased, blue: decreased. Solid arrows indicate direct reactions, dotted arrows indicate the reaction is broken by 1 or more sub-reactions. The tissue that genes and metabolites have been detected to be significantly altered in are depicted by the presence of tibialis anterior, spinal cord or liver image. The timepoint at which the gene or metabolite was detected as significantly changed is indicated beside the rectangle/circle. For transcriptomic data *n* = 4 male animals per genotype, per timepoint, per tissue. For plasma metabolomics data *n* = 6–15 animals (*n* = 3–8 male animals and *n* = 3–7 female animals) per genotype, per timepoint. For tissue metabolomics data *n* = 4–6 male animals, per genotype, per timepoint, per tissue. Created in BioRender. Price, G. (2026) https://BioRender.com/9llufvl. Relates to Supplementary Figs 25 and 26.

Integrated analysis of significant DEGs (across 6-, 12- and 20-month timepoints) and significantly changed metabolites (in blood collected at 4-, 6-, 9-, 13- and 20-months and the TA and SC at 20-months) highlighted energy metabolism pathways encompassing glycolysis, lipid utilization and the TCA cycle in both *hSOD1^WT/A5V^* and *hSOD1^A5V/A5V^* mouse models (Fig. 5 and Supplementary Fig. 25).

Integrated analysis of significantly changed genes and metabolites in *hSOD1^WT/A5V^* animals produced a model in which the majority of identified genes encode key enzyme intermediates of glycolysis and the citric acid cycle. Genes encoding enzymes involved in pyruvate production and transport were downregulated as were genes encoding enzymes involved in the production of fructose-6-phosphate. Metabolites highlighted through integrated analysis included increased levels of glucose together with increased levels of lipid substrates (Supplementary Fig. 25). Despite downregulation of genes related to glycolysis, there is an enrichment of upregulated genes involved in the formation and activity of complexes I, IV, and V of the electron transport chain (Supplementary Fig. 26A). Integrated analysis of significantly changed genes and metabolites in *hSOD1^WT/A5V^* compared with *hSOD1^WT/WT^* littermate controls identified dysregulation in additional pathways including xanthine and NAD metabolism (Supplementary Fig. 26B and C).

Integrated analysis of significant DEGs and metabolites in *hSOD1^A5V/A5V^* animals produced a model highlighting several metabolic pathways including lipid utilization and auxiliary TCA metabolism pathways (Fig. 5). The model shows an increase in the central glycolysis metabolite pyruvate in *hSOD1^A5V/A5V^* animals compared with *hSOD1^WT/WT^* littermate controls, whilst genes encoding enzymes for the conversion of malate into pyruvate and pyruvate into acetyl-co-A are downregulated. Lipid substrates for metabolism, including acyl-carnitines and fatty acids, are increased. The asymmetric dimethylarginine (ADMA) degradation pathway, linked to the urea cycle and fumarate production, is implicated in the model. The gene encoding the enzyme responsible for converting ADMA to citrulline, *Ddah1*, is significantly upregulated whilst citrulline is significantly decreased. Sarcosine metabolism is also highlighted, with increased levels of glyceric acid, glycine and sarcosine detected in the blood of 20-month animals compared with *hSOD1^WT/WT^* littermate controls. The glutamate-glutamine cycle also shows up with significant DEGs and metabolites in *hSOD1^A5V/A5V^* animals. The gene, *Gpt2*, encoding an enzyme involved in the conversion of α-ketoglutaric acid to glutamic acid is downregulated as are genes encoding enzymes involved in the conversion of glutamic acid to ornithine and aspartate. Further, glutamine is significantly increased whilst glutaric acid is significantly decreased (Fig. 5A). Integrated analysis of significant DEGs and metabolites in *hSOD1^A5V/A5V^* compared with *hSOD1^WT/WT^* littermate controls highlighted dysregulation in additional pathways including the leucine and isoleucine degradation pathway and aromatic acid detoxification (Fig. 5B and C).

The integrated analyses of significant DEGs and metabolites in *hSOD1^A5V^* animals compared with *hSOD1^WT/WT^* littermate controls suggests *SOD1^A5V^* mutation results in dysregulation of glycolysis, the TCA cycle and lipid utilization in both the *hSOD1^A5V^* heterozygous and homozygous mouse models. Further, the electron transport chain is also highlighted by the presence of dysregulated gene expression in *hSOD1^WT/A5V^* animals whilst auxiliary TCA pathways are defined in the *hSOD1^A5V/A5V^* animals. Overall, the integrated analysis of our transcriptomic and metabolomic data suggests a metabolic signature of disease in the absence of overt neurodegeneration.

## Discussion

Here, we present the characterization of the first genomically humanized *SOD1* knock-in mouse model for investigating ALS, offering insights into potential metabolic perturbations associated with SOD1-ALS mutation in the absence of neurodegeneration. Using our knock in humanized model we were able to study heterozygous and homozygous mutation effects. This is important as in addition to SOD1 gain of function (GOF) phenotypes, SOD1-ALS patients may have some degree of SOD1 loss of function (LOF), because enzymatic activity is typically reduced in the majority of variants.^9^ Furthermore, SOD1 LOF has a more severe impact on neurological function in humans than in mice,^9,46-48^ potentially because *SOD1* is an essential gene in humans whereas this is not the case in mice.^48,49^ Therefore, both GOF and LOF phenotypes remain relevant for ALS disease modelling.

We observed reduced SOD1 levels and activity in *hSOD1^A5V^* mice, which is consistent with results from SOD1-A5 V patients.^9,49^ We found particularly low SOD1 activity in skeletal muscle of *hSOD1^A5V^* mice, especially when compared with wildtype *mSod1*/*mSod1* mice (i.e. both mouse *Sod1* genes intact) (Supplementary Fig. 27). This may be due to the compounded effects of lower SOD1 transcription in muscle in *hSOD1^WT/WT^* mice versus *mSod1*/*mSod1,*^22^ plus the decreased stability of SOD1-A5 V (versus SOD1-WT) in *hSOD1^A5V^* mice. The minimum level of SOD1 required to prevent LOF phenotypes in mice is unknown, and it is possible that LOF phenotypes may be observed in *hSOD1^A5V^* mice, and potentially SOD1-A5 V patients.

The presence of aggregated SOD1 protein species, which arise from the misfolding and accumulation of soluble disordered SOD1, is a hallmark of ALS observed in SOD1-ALS patients and mouse models.^50-53^ Increased levels of soluble disordered SOD1 were detected in the tissues of *hSOD1^A5V^* animals, in the absence of protein aggregates, potentially suggesting a pre-manifest prodromal state of disease.^13,15^ In mice, high levels of SOD1 protein are found in the brain and liver, providing an explanation for a significant increase in disordered SOD1 detected in these tissues. We did not observe development of motor symptoms in *hSOD1^A5V^* mice, suggesting that expression of the A5 V mutation at physiological levels may be insufficient to drive the classical ALS phenotype observed in transgenic overexpression models, within the lifespan of this strain of mouse. These findings are consistent with those of the low copy transgenic *tg.SOD1^A5V^* strain, in which a low level of mutant SOD1 protein and lack of protein aggregation were accompanied by an absence of a motor phenotype.^16^ However, a phenotype was observed when these *tg.SOD1^A5V^* mice were crossed with a line overexpressing wildtype hSOD1 at a high level, suggesting that wildtype SOD1 can act as a substrate for aggregation and that total hSOD1 levels are a limiting factor for neurodegeneration in models expressing mutant SOD1 transgenes.^18^

To our knowledge, there is only one other SOD1-A5 V mutant mouse, a *Sod1^A5V^* knock-in strain in which the A5 V mutation has been introduced into the mouse *Sod1* gene (JAX 031341). This strain also has no documented phenotype on the JAX strain description. A small number of different low-copy transgenic SOD1-ALS models exist that show motor phenotypes late in life,^16,18,54,55^ but there has been no direct comparison of protein levels between these different models, noting that transgenic copy number is not directly proportional to translational output.^56^ Thus, the simplest explanation for a lack of motor phenotypes in A5 V models is low SOD1 levels and therefore, insufficient disordered SOD1 to initiate fulminant aggregation and the likely related neurotoxic cascade.

Several observations support a mild metabolic phenotype in *hSOD1^A5V^* mice, which could be linked to gain or loss of SOD1 function, or both. There are similarities between the phenotypes observed in *hSOD1^A5V^* and published observations from *Sod1*^−/−^ mice, which may indicate a SOD1 LOF effect. Female *hSOD1^A5V/A5V^* animals had reduced bodyweight versus *hSOD1^WT/WT^* littermate controls and a trending decrease in fat mass; *Sod1*^−/−^ mice also display reduced bodyweight and decreased visceral fat.^45,57,58^  *hSOD1^A5V^* mice presented with an impaired early response to glucose; disturbed glucose metabolism has also been observed in *Sod1*^−/−^ mice.^45,59^ We note that other than this difference in bodyweight between female mutants and wildtype controls, we found no differences between male and female mice—in human SOD1-A5 V ALS cases, the prevalence of disease is similar in males and females and we can find no mention of male versus female disease manifestation for this specific mutation in the literature.^60^

Transcriptomic and metabolomic analysis of blood plasma, SC, and TA highlighted a significant dysregulation of genes and metabolites involved in glycolysis, the TCA cycle, electron transport chain, and lipid metabolism. *Sod1^−/−^* mice have altered glycolysis and gluconeogenesis and disrupted lipid metabolism, with altered hepatic and plasma lipid profiles and altered lipogenesis.^57,61,62^ However, several SOD1-ALS mouse models, without loss of SOD1 enzymatic activity, display metabolic phenotypes including reduced bodyweight, reduced fat mass, dyslipidaemia, and reduced glucose utilization,^38^ suggesting gain of function could also be an underlying cause.

The alterations identified in our *hSOD1^A5V^* mice align with growing evidence implicating metabolic dysfunction as a critical feature of ALS pathogenesis. Recently, neuronal polyunsaturated fatty acids have been shown to have a protective effect in *Drosophila* of ALS expressing the pathological *C9orf72* repeat expansion.^63^ The polyunsaturated fatty acids found to be downregulated in affected flies overlap (with the same direction of change) with those detected in our *hSOD1^A5V^* mice, including eicosapentaenoic acid, palmitoleic acid, palmitic acid and oleic acid. Giblin *et al*.^63^ also highlight reduced expression of the genes fatty acid synthase (*FASN*) and very long chain fatty acid elongase 6 (*ELOVL6*) in C9orf72-ALS *Drosophila* and post-mortem spinal cord from C9orf72-ALS patients.^63^ This is consistent with data from our *hSOD1^A5V^* mice in which *Fasn* is significantly downregulated in the SC of 20-month-old *hSOD1^WT/A5V^* (*P* = 0.044) and the TA of 12-month-old *hSOD1^WT/A5V^* (*P* = 0.047) animals and expression of *Elovl6* is significantly reduced in the SC of 12-month-old *hSOD1^WT/A5V^* mice (*P* = 0.045) (Supplementary Figs 11C and 12C, E). The prospect of a shared metabolic signature involving fatty acid changes across different ALS models with various mutations is a key area for future research with the potential to develop treatments applicable to all ALS patients, regardless of their specific mutation. Previous studies in ALS patients and transgenic mouse models have demonstrated perturbations in glucose metabolism, mitochondrial dysfunction, and energy deficits.^34,36,37^ The increased pyruvic acid and TCA intermediates and dysregulated lipid metabolism in our model suggest a shift towards alternative metabolic pathways, possibly as a compensatory response to impaired energy homeostasis. Moreover, the upregulation of genes involved in mitochondrial electron transport chain complexes in *hSOD1^WT/A5V^* animals suggests an attempt to counteract mitochondrial dysfunction, a well-established feature of ALS.^17,64^

Despite the lack of overt neurodegeneration, our findings suggest that metabolic dysregulation may precede the clinical manifestation of ALS. This aligns with the concept of a pre-symptomatic metabolic shift, where bioenergetic changes occur before significant neuromuscular impairment.^65,66^ The identification of altered pathways related to glycolysis, lipid metabolism, and oxidative phosphorylation raises the possibility that metabolic biomarkers could serve as early indicators of ALS risk or disease progression.

The absence of neurodegeneration in our model is a limitation but reinforces the need for additional triggers beyond SOD1 mutation alone for ALS symptoms to develop.^1^ Time is likely an important factor, whereby physiological levels of SOD1 may require longer than the lifespan of the mouse to reach a threshold of disordered SOD1 needed to manifest clinical phenotypes. We also note that the animals in this study had a single genetic background and thus provided only a snapshot of possible genetic interactions. Another possibility is that species intrinsic differences between mouse and human preclude the development of ALS in such models—although overexpression of various SOD1 pathogenic variants in mice lead to ALS phenotypes, suggesting there is no such intrinsic limitation.^17^ Future studies should explore whether aging, environmental factors, or seeding paradigms induce more advanced phenotypes in these mice. Exposure of our humanized *hSOD1^A5V^* mice to an oxidant diet may exacerbate or expose further ALS-like phenotypes due to the paucity of SOD1 enzymatic activity.^67^ Additionally, targeting the metabolic pathways identified in our study, such as glycolysis and lipid metabolism, may provide novel therapeutic strategies for delaying ALS onset in genetically predisposed individuals.^34,36^ Our findings highlight the importance of preclinical ALS models that mimic the clinically silent phase of disease, allowing for biomarker discovery and new mechanistic insights into ALS aetiology. The presence of the human *SOD1* gene at the endogenous locus also offers a platform to test therapeutics that target the human gene or gene products in a physiological context.

The observed metabolic and transcriptomic changes suggest that early dysfunctions in energy metabolism may precede motor impairment, aligning with clinical observations in pre-symptomatic ALS patients. While our model is focused on the early stages of ALS, transgenic overexpression mice remain valuable for studying later disease stages characterized by aggressive neurodegeneration.^19,68^ Integrating these different models will provide a more comprehensive understanding of ALS pathogenesis and facilitate the development of interventions tailored to specific disease phases. Our humanized *hSOD1^WT^* and *hSOD1^A5V^* mouse strains are freely available from EMMA with the IDs EM:13075 and EM:14880, respectively.

## Supplementary Material

fcag302_Supplementary_Data

## Data Availability

The official allele designation of the humanized *hSOD1^A5V^* allele is Sod1^em1(SOD1)Emcf^ (MGI:6856511). The official strain name of the humanized *hSOD1^A5V^* mice on a C57BL/6J background is B6.129-Sod1^em1(SOD1)Emcf^/H (MGI:6856512). This line is freely available from the European Mouse Mutant Archive (EM:14880). Codes used for data analysis are available at https://github.com/Chloe-J-W/RNA-seq.git. Data used for analysis are attached in the following supplementary Data files: Supplementary Data 1—significantly differently expressed genes in the tibialis anterior and spinal cord of *hSOD1^WT/A5V^* and *hSOD1^A5V/A5V^* animals compared with littermate controls (*Padj* < 0.1). Supplementary Data 2—overlap between datasets outputted from VennPlots. Supplementary Data 3—top 10 most upregulated and downregulated genes detected. Supplementary Data 4—all differentially expressed genes in the tibialis anterior of *hSOD1^WT/A5V^* and *hSOD1^A5V/A5V^* animals compared with littermate controls. Supplementary Data 5—all differentially expressed genes in the spinal cord of *hSOD1^WT/A5V^* and *hSOD1^A5V/A5V^* animals compared with littermate controls. Supplementary Data 6—raw RNA sequencing reads. Supplementary Data 7—raw metabolite measurements. Supplementary Data 8—retrospective power analysis. Supplementary Data 9—raw data relevant for main and supplementary figures. Supplementary Methods and Supplementary Figures are within Supplementary material.
