# Supplementary material for: Metabolic alterations in the absence of a neuromuscular phenotype in humanized SOD1A5V mice
Source: Brain Commun. 2026 Sep 17;8(5):fcag302. doi: 10.1093/braincomms/fcag302 (PMC13583902; doi:10.1093/braincomms/fcag302)
Supplement: Supplementary file 1 [file fcag302_supplementary_data.zip › SUPPLEMENTARY_MATERIAL.pdf]

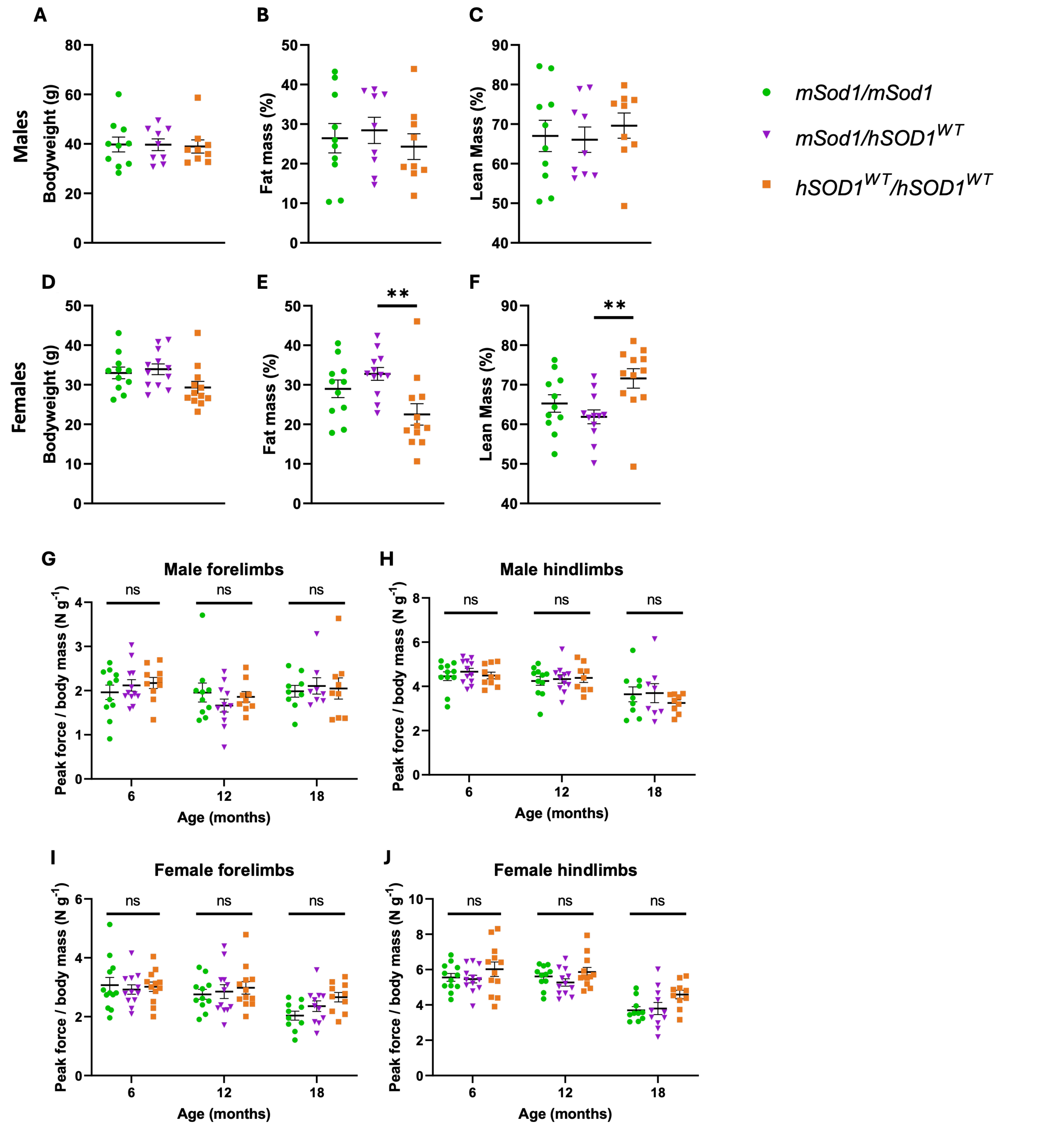

Supplementary Figure 1. Echo MRI data and longitudinal grip strength data from *hSOD1^WT* mice. Total bodyweight, fat mass and lean mass in 15-month-old (A-C) male (9-10 per genotype) and (D-F) female (11-12 per genotype) *hSOD1^WT* animals. Mean ± SEM. One-way ANOVA with Tukey's post hoc test. Main effects: A) $F(2,25) = 0.0252$, $P = 0.975$; B) $F(2,25) = 0.342$, $P = 0.714$; C) $F(2,25) = 0.265$, $P = 0.769$; D) $F(2,32) = 2.86$, $P = 0.0721$; E) $F(2,32) = 5.51$, $P = 0.00881$; F) $F(2,32) = 5.39$, $P = 0.00961$. $^{*}P < 0.05$, $^{**}P < 0.01$. Grip strength data from (G, H) male (8-12 per genotype) and (I, J) female (10-12 per genotype) *hSOD1^WT* mice. Mean ± SEM. Two-way ANOVA with Tukey's post hoc test. Genotype effect: G) $F(2,80) = 0.138$, $P = 0.871$; H) $F(2,81) = 0{,}534$, $P = 0.588$; I) $F(2,93) = 1.43$, $P = 0.245$; J) $F(2,93) = 5.01$, $P = 0.00856$. ns, not significant. Each datapoint represents an individual animal.

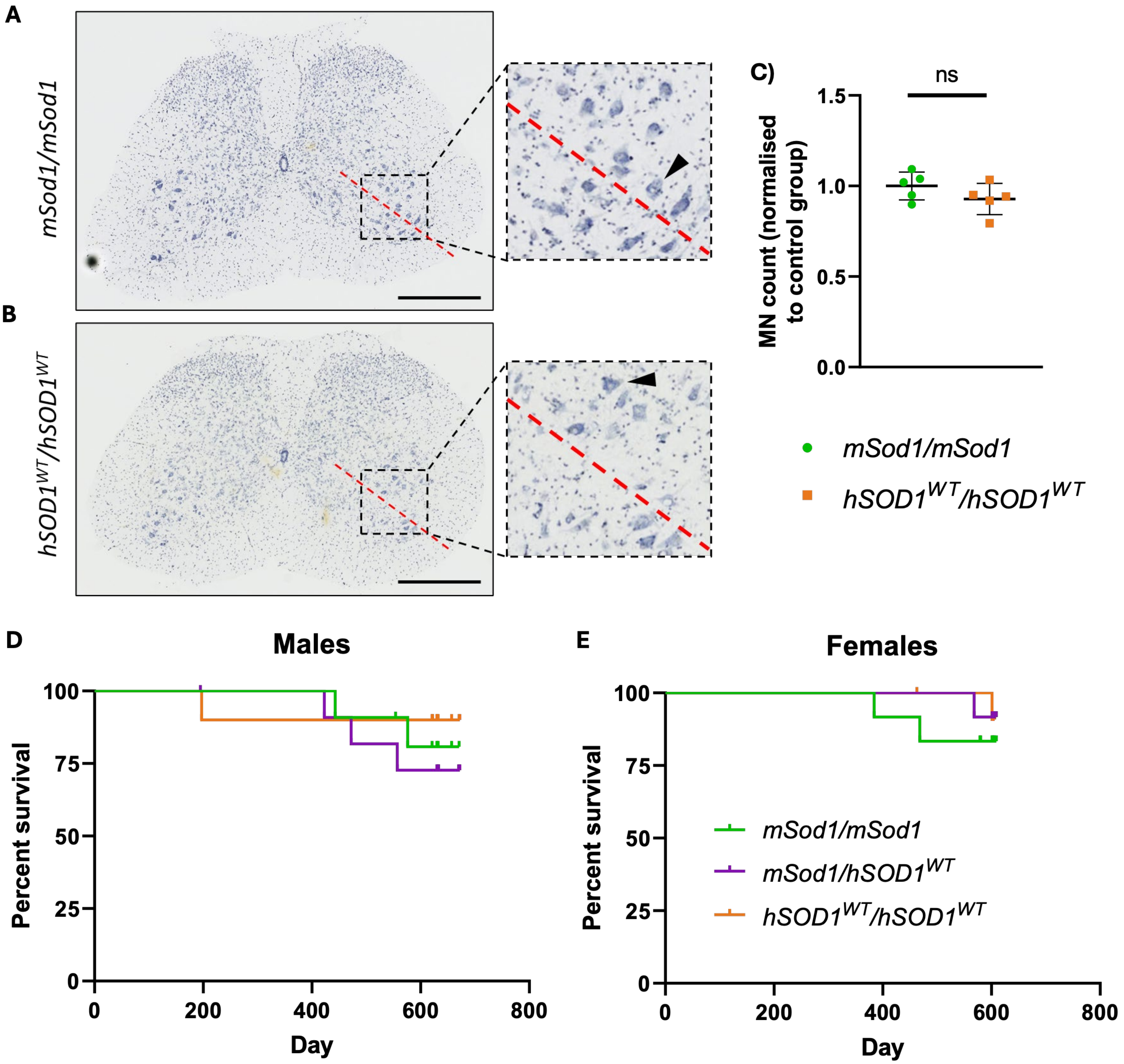

Supplementary Figure 2. Spinal motor neuron counts and Kaplan-Meier survival graphs for *hSOD1*^{WT} mice. Representative images of lumbar spinal cord sections from 18-month-old **(A)** mSod1/mSod1 and **(B)** *hSOD1*^{WT}/*hSOD1*^{WT} mice. Red dotted line indicates the lower boundary of the sciatic motor pool. Only motor neurons with cell bodies fully above this line were included in motor neuron counts. Arrowheads indicate cells displaying characteristic motor neuron features: polygonal shape, dense staining and a diameter greater than 20 µm. Scale bar = 500 µm. **(C)** Quantification of motor neurons within the sciatic pool of the lumbar spinal cord (L3-L5) of 18-month-old male *hSOD1*^{WT} animals. 5 animals per genotype. Mean ± SEM. Unpaired two-tailed t-test. $t(8) = 1.39$, $P = 0.203$. ns, not significant. Kaplan-Meier curves for **(D)** male (10-12 per genotype) and **(E)** female (12 per genotype) *hSOD1*^{WT} mice. Mice that died from common intercurrent illnes (e.g. tumours, dermititis) or from fighting, or culled for tissue collection were censored at the time of death. Censored animals are indicated by tick marks. The study end point was approximately 650 days old for male animals and 600 days old for female animals.

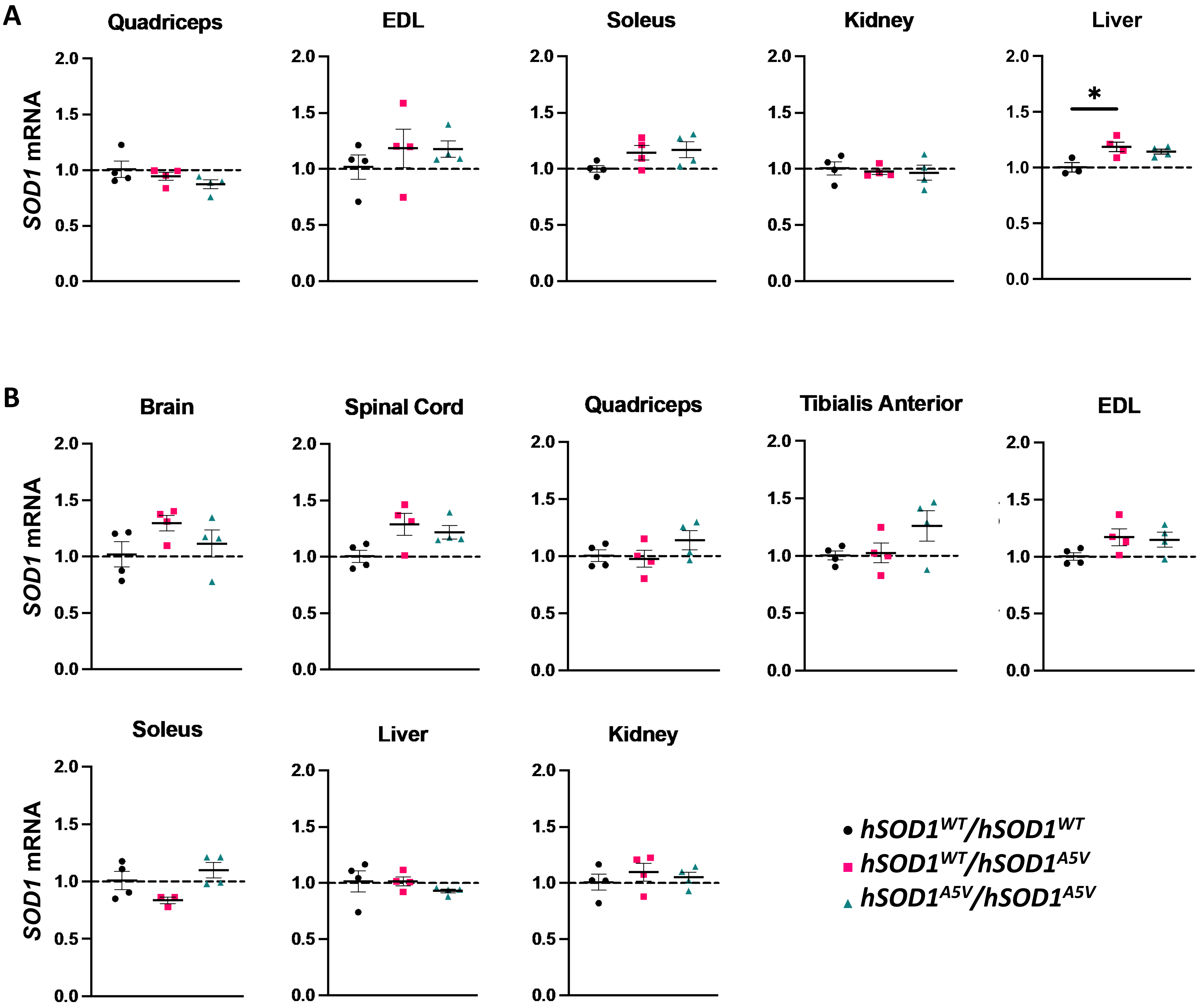

Supplementary Figure 3. *SOD1* mRNA expression in *hSOD1^{A5V}* mice. Expression of total *SOD1* mRNA using conserved mouse-human *SOD1* primers in tissues from 3-month-old A) male and B) female animals (3-4 animals per sex per genotype per tissue). All values are expressed as a fold change relative to the average *SOD1* expression in *hSOD1$^{WT/WT}$* control animals. Each datapoint represents an individual animal. Mean ± SEM. One-way ANOVA with Tukey's post hoc test. Main effects: A) quadriceps $F(2,9) = 1.650$, $P = 0.245$; EDL $F(2,9) = 0.554$, $P = 0.593$; soleus $F(2,9) = 2.49$, $P = 0.138$; kidney $F(2,9) = 0.144$, $P = 0.868$; liver $F(2,8) = 6.33$, $P = 0.0225$; B) brain $F(2,9) = 1.88$, $P = 0.208$; spinal cord $F(2,9) = 4.10$, $P = 0.0544$; quadriceps $F(2,9) = 1.55$, $P = 0.263$; tibialis anterior $F(2,9) = 2.31$, $P = 0.155$; EDL $F(2,9) = 2.34$, $P = 0.152$; soleus $F(2,8) = 3.72$, $P = 0.0721$; kidney $F(2,9) = 0.443$, $P = 0.655$; liver $F(2,9) = 0.662$, $P = 0.539$. $*P < 0.05$. EDL, extensor digitorum longus. Relates to Fig. 1.

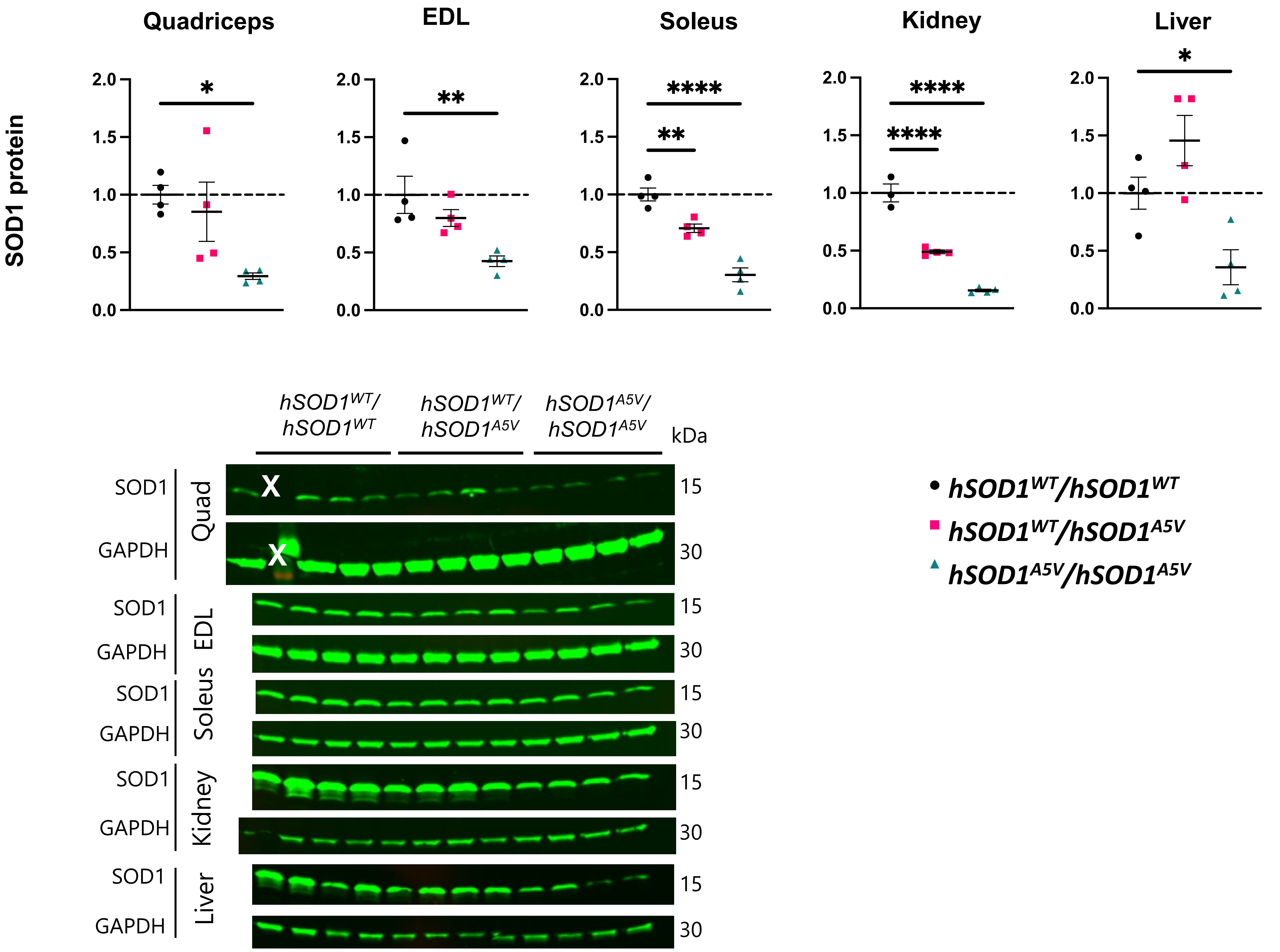

Supplementary Figure 4. SOD1 protein male *hSOD1^{A5V}* mice. Western blots using a pan-mouse-human SOD1 antibody (epitope: amino acids 131-153) in tissues from 3-month-old male animals. Human SOD1 has lower mobility than mouse SOD1 in SDS-PAGE (sodium dodecyl sulfate-PolyAcrylamide gel electrophoresis) and appears as a higher band. Blots normalized to GAPDH. All values are expressed as a fold change relative to the average SOD1 level in *hSOD1$^{WT/WT}$* control animals. 4 per genotype per tissue. White crosses indicate lanes that were excluded from the analysis due to gel loading issues. Each datapoint represents an individual animal. Mean ± SEM. One-way ANOVA with Tukey's post hoc test. Main effects: quadriceps $F(2,9) = 5.70$, $P = 0.0251$; EDL $F(2,9) = 7.72$, $P = 0.0112$; soleus $F(2,9) = 45.9$, $P = 1.91 \times 10^{-5}$; kidney $F(2,8) = 123$, $P = 9.79 \times 10^{-7}$; liver $F(2,9) = 10.1$, $P = 0.00500$. For clarity, only significant comparisons to *hSOD1$^{WT/WT}$* controls are displayed. $^{*}P < 0.05$, $^{**}P < 0.01$, $^{***}P < 0.001$, $^{****}P < 0.0001$. EDL, extensor digitorum longus. Relates to Fig. 1. Full uncropped blots are shown in Supplementary Figure 28.

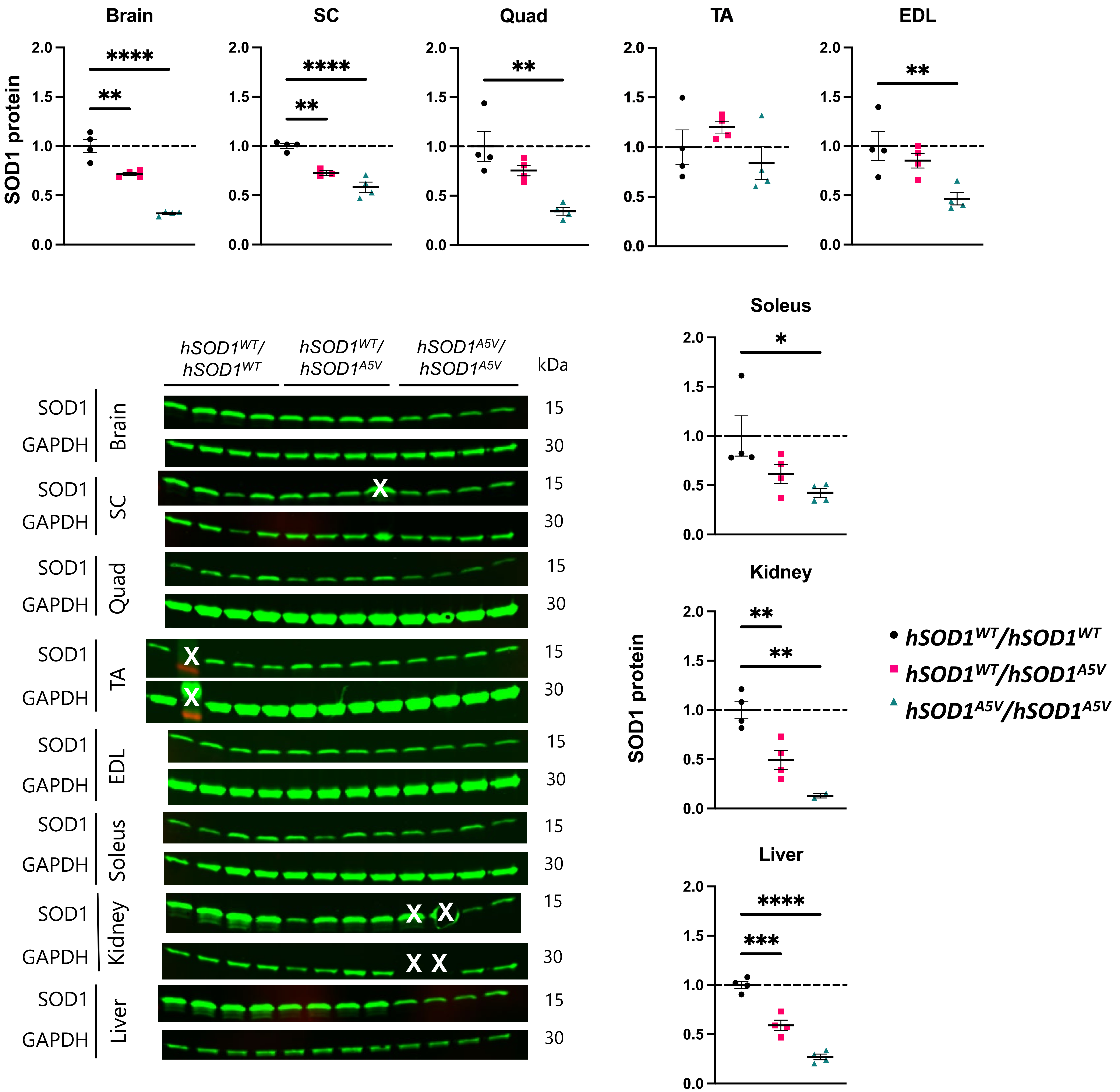

Supplementary Figure 5. SOD1 protein female *hSOD1^{A5V}* mice. Western blots using a pan-mouse-human SOD1 antibody (aa131-153) in tissues from 3-month-old female animals. Human SOD1 has lower mobility than mouse SOD1 in SDS-PAGE (sodium dodecyl sulfate-PolyAcrylamide gel electrophoresis) and appears as a higher band. Blots normalized to GAPDH. All values are expressed as a fold change relative to the average SOD1 level in *hSOD1$^{WT/WT}$* control animals. 4 animals per genotype per tissue. White crosses indicate lanes that were excluded from the analysis due to gel loading issues. Each datapoint represents an individual animal. Mean ± SEM. One-way ANOVA with Tukey's post hoc test. Main effects: brain $F(2,9) = 70.2$, $P = 3.24 \times 10^{-6}$; spinal cord $F(2,8) = 34.2$, $P = 1.21 \times 10^{-4}$; quadriceps $F(2,9) = 12.3$, $P = 0.00266$; tibialis anterior $F(2,9) = 1.60$, $P = 0.255$; EDL $F(2,9) = 7.30$, $P = 0.0131$; soleus $F(2,9) = 4.86$, $P = 0.0371$; kidney $F(2,7) = 18.9$, $P = 0.00151$; liver $F(2,9) = 78.4$, $P = 2.02 \times 10^{-6}$. For clarity, only significant comparisons to *hSOD1$^{WT/WT}$* controls are displayed. $^{*}P < 0.05$, $^{**}P < 0.01$, $^{***}P < 0.001$, $^{****}P < 0.0001$. SC, spinal cord; TA, tibialis anterior; EDL, extensor digitorum longus. Relates to Fig. 1. Full uncropped blots are shown in Supplementary Figure 28.

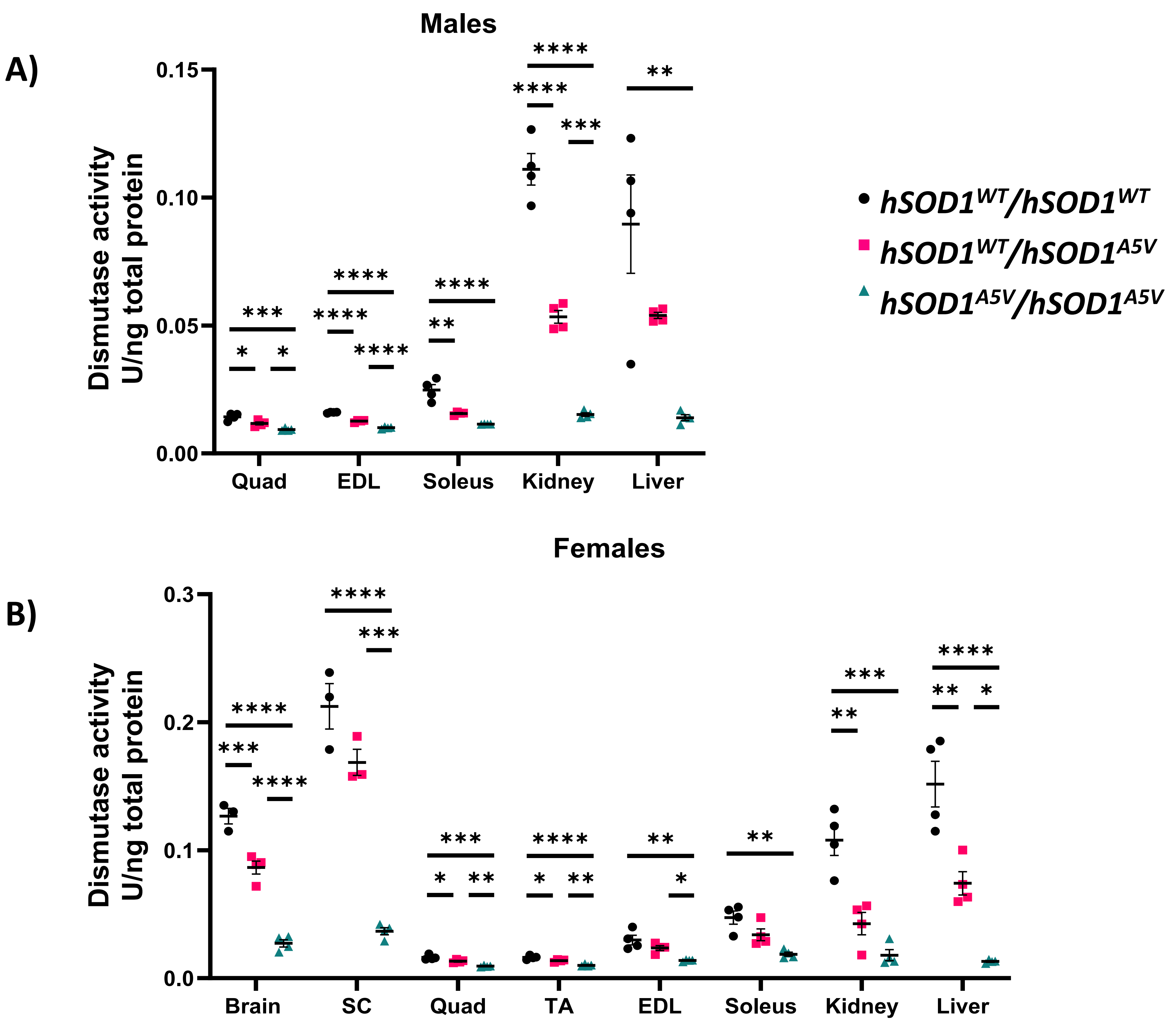

Supplementary Figure 6. SOD1 activity in *hSOD1*^{A5V} mice. A) Spectrophotometric dismutase activity assays in tissues from male 3-month-old *hSOD1*^{A5V} mice. 3-4 animals per genotype per tissue. B) Spectrophotometric dismutase activity assays in tissues from female 3-month-old *hSOD1*^{A5V} mice. 3-4 animals per genotype per tissue. Mean ± SEM. One-way ANOVA with Tukey's post hoc test. Each data point represents an individual animal. Main effects: A) quadriceps $F(2,9) = 20.6$, $P = 4.35 \times 10^{-4}$; EDL $F(2,9) = 260$, $P = 1.10 \times 10^{-8}$; soleus $F(2,9) = 31.35$, $P = 8.79 \times 10^{-5}$; kidney $F(2,9) = 157$, $P = 1.01 \times 10^{-7}$; liver $F(2,9) = 11.6$, $P = 0.00326$; B) brain $F(2,8) = 113$, $P = 1.38 \times 10^{-6}$; SC $F(2,7) = 80.2$, $P = 1.50 \times 10^{-5}$; quadriceps $F(2,9) = 25.6$, $P = 1.92 \times 10^{-4}$; TA $F(2,9) = 29.1$, $P = 1.17 \times 10^{-4}$; EDL $F(2,9) = 11.1$, $P = 0.00374$; soleus $F(2,9) = 12.3$, $P = 0.00267$; kidney $F(2,9) = 27.1$, $P = 1.55 \times 10^{-4}$; liver $F(2,9) = 36.2$, $P = 4.97 \times 10^{-5}$. $*P < 0.05$, $**P < 0.01$, $***P < 0.001$, $****P < 0.0001$. SC, spinal cord; TA, tibialis anterior; EDL, extensor digitorum longus. Relates to Fig. 1.

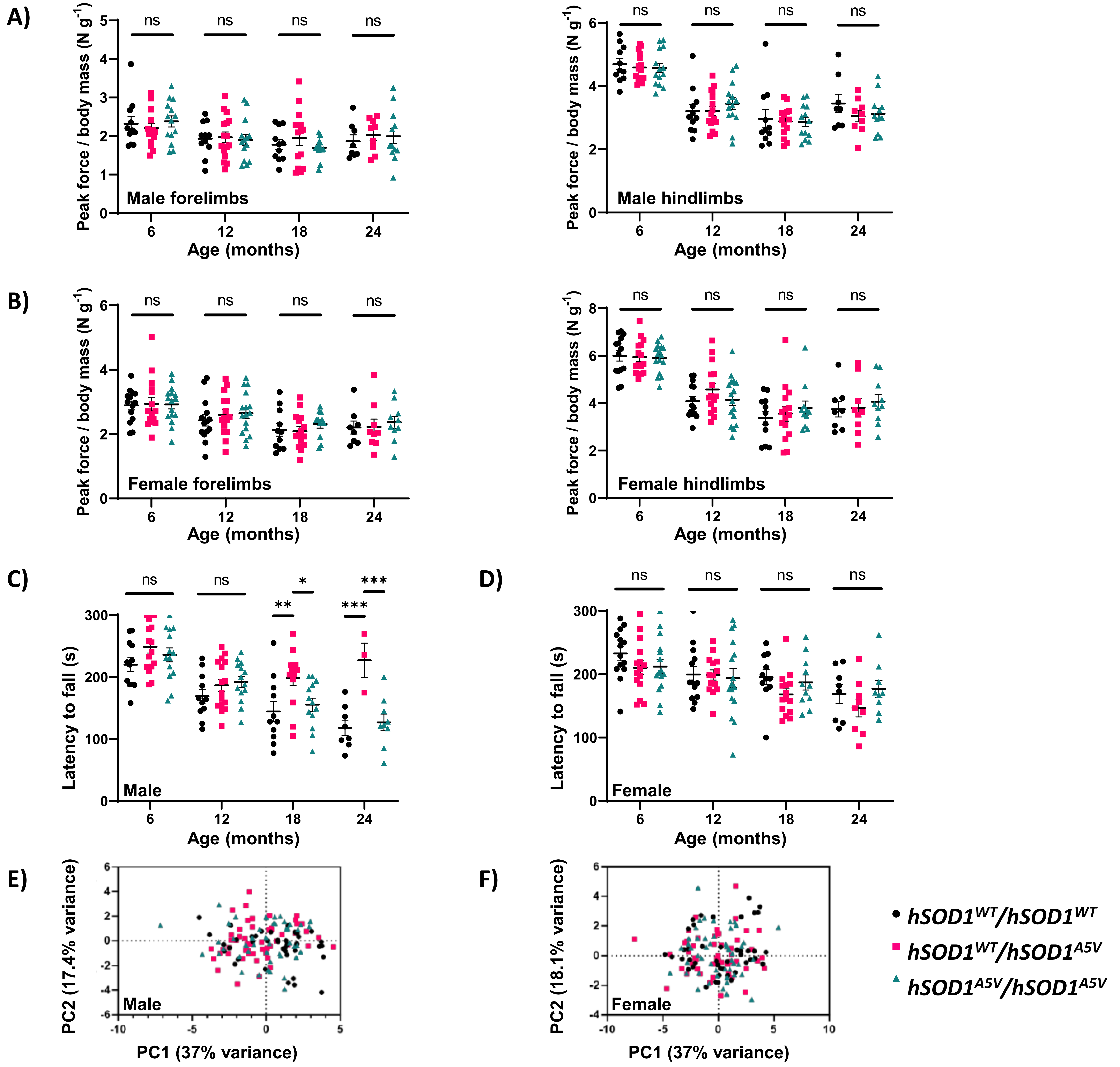

Supplementary Figure 7. Motor function phenotyping in *hSOD1*^{A5V} mice. A) Grip strength data from male forelimb and hindlimb (8-16 per genotype) *hSOD1*^{A5V} mice. B) Grip strength data from female forelimb and hindlimb (9-16 per genotype) *hSOD1*^{A5V} mice. C) Rotarod data from male (8-16 per genotype) *hSOD1*^{A5V} mice. D) Rotarod data from female (9-16 per genotype) *hSOD1*^{A5V} mice. Mean ± SEM. Two-way ANOVA with Tukey's post hoc test. Genotype effect: A) forelimbs $F(2,136) = 0.137$, $P = 0.872$; hindlimbs $F(2,136) = 0.559$, $P = 0.573$; B) forelimbs $F(2,142) = 0.754$, $P = 0.472$; hindlimbs $F(2,142) = 0.534$, $P = 0.588$; C) $F(2,128) = 14.9$, $P = 1.51 \times 10^{-6}$; D) $F(2,139) = 2.26$, $P = 0.108$. Reported significance values are from Tukey's multiple comparisons test. *$P < 0.05$. **$P < 0.01$, ***$P < 0.001$. ns, not significant. E) Principal component analysis (PCA) using all 13 gait analysis parameters (walking speed, forelimb and hindlimb stance duration, step length, stride length, stride width, swing duration and duty factor) as variables in male *hSOD1*^{A5V} mice. 41-60 experimental runs per genotype (8-17 animals per genotype per timepoint across 4 timepoints; 6, 12, 18 and 24 months) F) Principal component analysis (PCA) using all 13 gait analysis parameters (walking speed, forelimb and hindlimb stance duration, step length, stride length, stride width, swing duration and duty factor) as variables in female *hSOD1*^{A5V} mice. 51-57 experimental runs per genotype (10-16 animals per genotype per timepoint across 4 timepoints; 6, 12, 18 and 24 months). Each datapoint represents an individual animal.

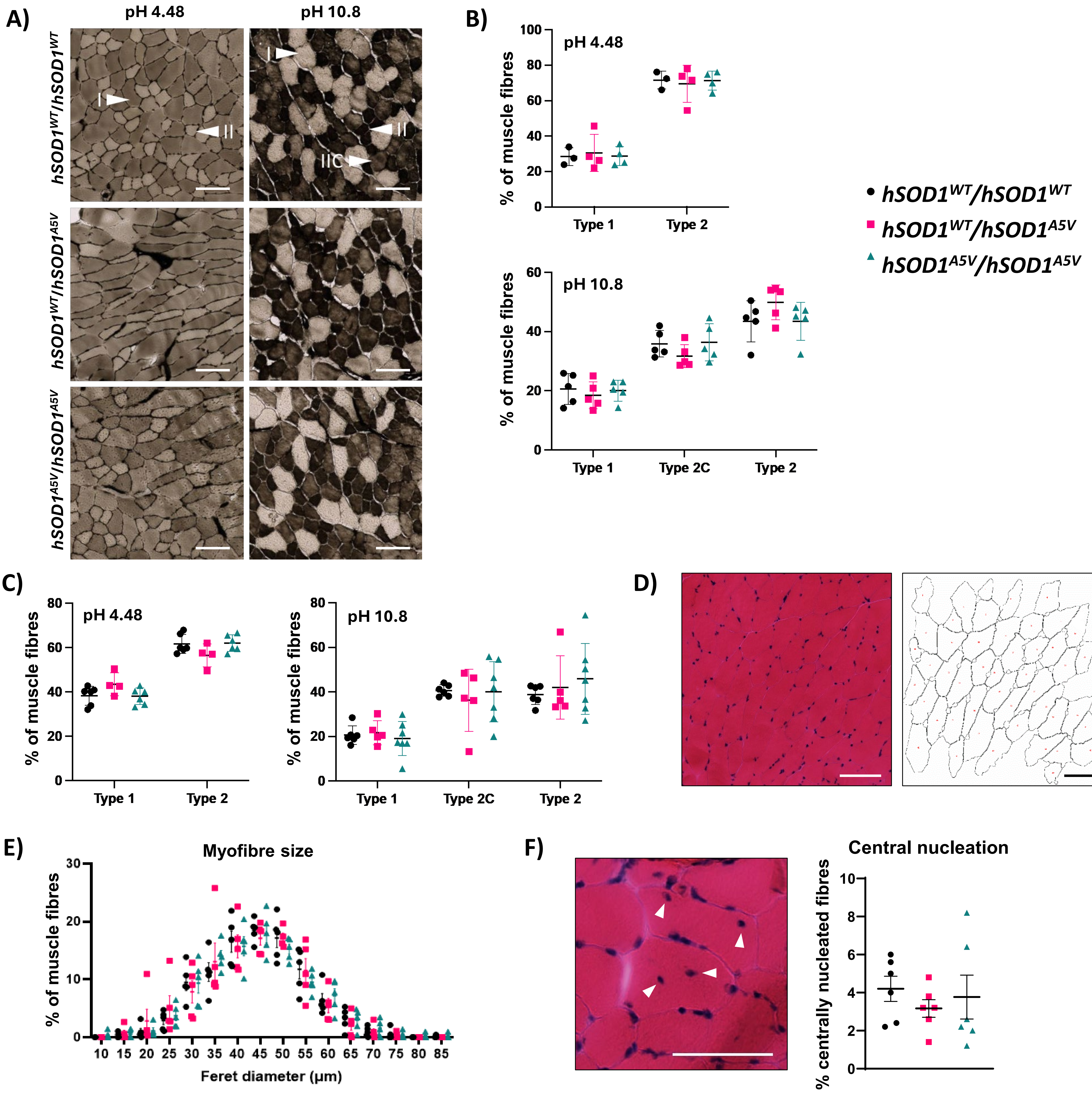

Supplementary Figure 8. Myofibre typing and analysis in *hSOD1*^{A5V} mice.
Legend on next page.

Supplementary Figure 8. Myofibre typing and analysis in *hSOD1^{A5V}* mice. A) Representative images of myosin-ATPase staining in TA (tibialis anterior) muscle from 6-month-old female *hSOD1^{A5V}* mice. Arrowheads indicate type I, type II and type IIC muscle fibres. Scale bar = 100µm. B) Quantification of myofiber type in TA muscle from 6-month-old female *hSOD1^{A5V}* mice (5 per genotype). Mean ± SEM. One-way ANOVA with Tukey's post hoc test. Main effects: pH4.48 $F(2,8) = 0.0858$, $P = 0.919$; pH10.8 Type 1 $F(2,12) = 0.315$, $P = 0.736$; pH10.8 Type 2C $F(2,12) = 1.34$, $P = 0.298$; pH10.8 Type 2 $F(2,12) = 1.64$, $P = 0.235$. C) Quantification of myofiber type in TA muscle from 27-month-old female *hSOD1^{A5V}* mice (5-7 per genotype). Mean ± SEM. One-way ANOVA with Tukey's post hoc test. Main effects: pH4.48 $F(2,13) = 2.31$, $P = 0.139$; pH10.8 Type 1 $F(2,15) = 0.292$, $P = 0.751$; pH10.8 Type 2C $F(2,15) = 0.241$, $P = 0.789$; pH10.8 Type 2 $F(2,15) = 0.514$, $P = 0.609$. D) Representative image of a H&E-stained TA muscle section and the corresponding segmented image used for Feret diameter determination. Scale bar = 100µm. E) Quantification of myofiber size in TA muscle from 27-month-old female *hSOD1^{A5V}* mice (5 per genotype), measured by the minimum Feret diameter. Mean ± SEM. Two-way repeated measures ANOVA with Tukey's post hoc test. Genotype effect: $F(2,12) = 0.764$, $P = 0.487$; F) Example of centrally nucleated myofibers. Arrowheads indicate centralised nuclei. Scale bar = 50µm. Percentage of centrally nucleated myofibers in TA muscle from 27-month-old female *hSOD1^{A5V}* mice (6 per genotype). Mean ± SEM. One-way ANOVA with Tukey's post hoc test. Main effect: $F(2,15) = 0.406$, $P = 0.674$. All panels: each datapoint represents an individual animal.

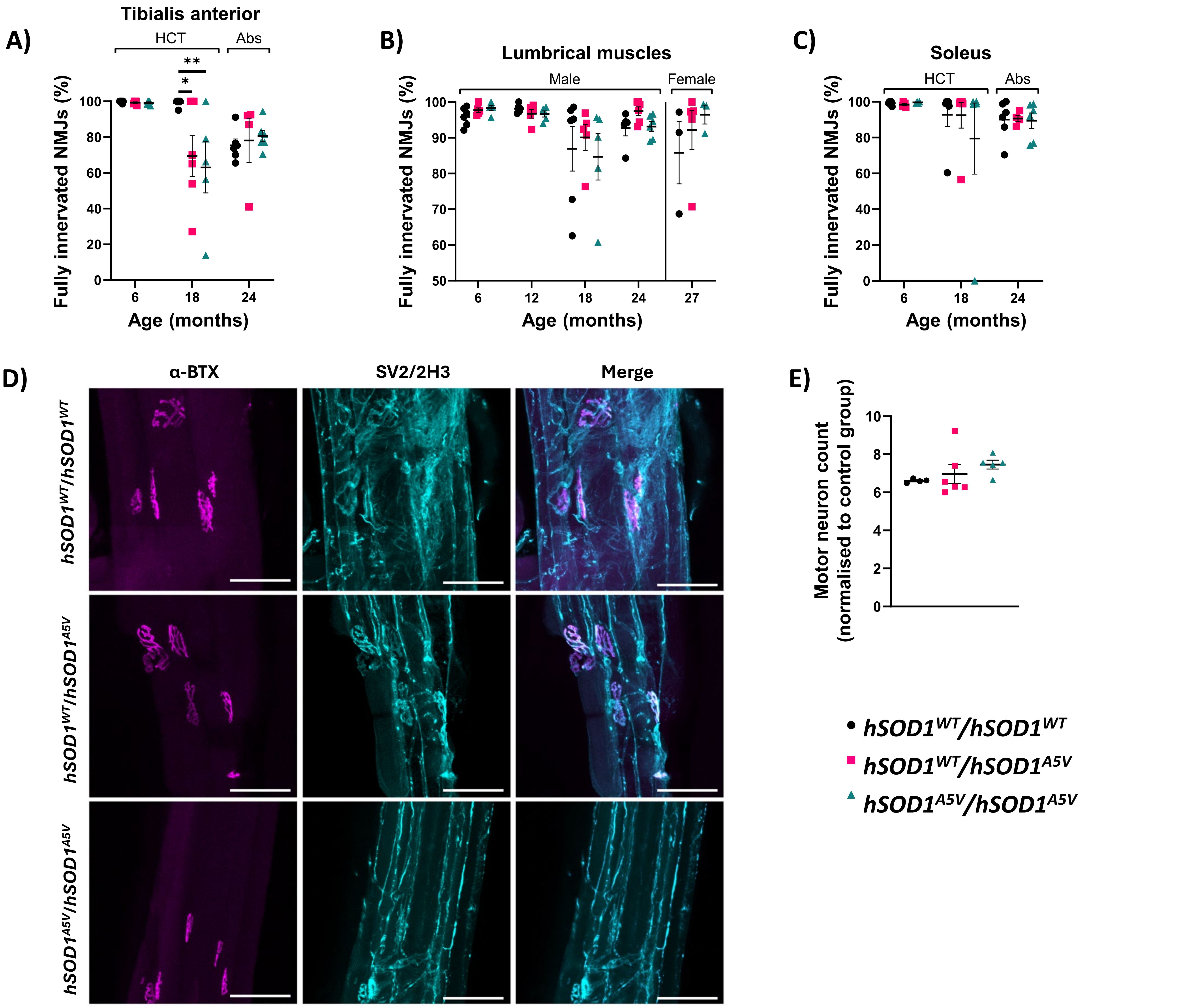

Supplementary Figure 9. Neuromuscular junction (NMJ) analysis and motor neuron counts in *hSOD1^{A5V}* mice.
A) Quantification of fully innervated NMJs in lumbrical muscles from 24-month-old male *hSOD1^{A5V}* mice (4-6 animals per genotype). B) Quantification of fully innervated NMJs in tibialis anterior muscles from 24-month-old male (5-6 animals per genotype) and female (3-5 animals per genotype) *hSOD1^{A5V}* mice. The axons and presynaptic termini were visualised using antibodies against synaptophysin/neurofilament. C) Quantification of fully innervated NMJs in soleus muscle from 24-month-old male *hSOD1^{A5V}* mice (4-6 animals per genotype). Two-way ANOVA with Tukey's post hoc test. Genotype effect: A) $F(2,39) = 1.95$, $P = 0.137$; B) $F(2,60) = 0.888$, $P = 0.417$; C) $F(2,38) = 0.311$, $P = 0.735$. Reported significance values are from Tukey's multiple comparisons test. Mean ± SEM. *$P < 0.05$. **$P < 0.01$. Abs, antibodies. D) Representative images of neuromuscular junction (NMJ) staining in lumbrical muscles from 24-month-old *hSOD1 ^{A5V}* mice. α-bungarotoxin (α-BTX), magenta, labels nicotinic acetylcholine receptors on the motor end plate; synaptophysin/neurofilament (SV2/2H3), cyan, label the axon and nerve terminal of motor neurons. Scale bar 100 µm. E) Quantification of motor neurons within the sciatic pool of the lumbar spinal cord (L3-L5) of 24-month-old male *hSOD1^{A5V}* animals; 4- 6 animals per genotype. One-way ANOVA with Tukey's post hoc test. Main effect: $F(2,12) = 1.19$, $P = 0.338$. Mean ± SEM. All panels: each datapoint represents an individual animal.

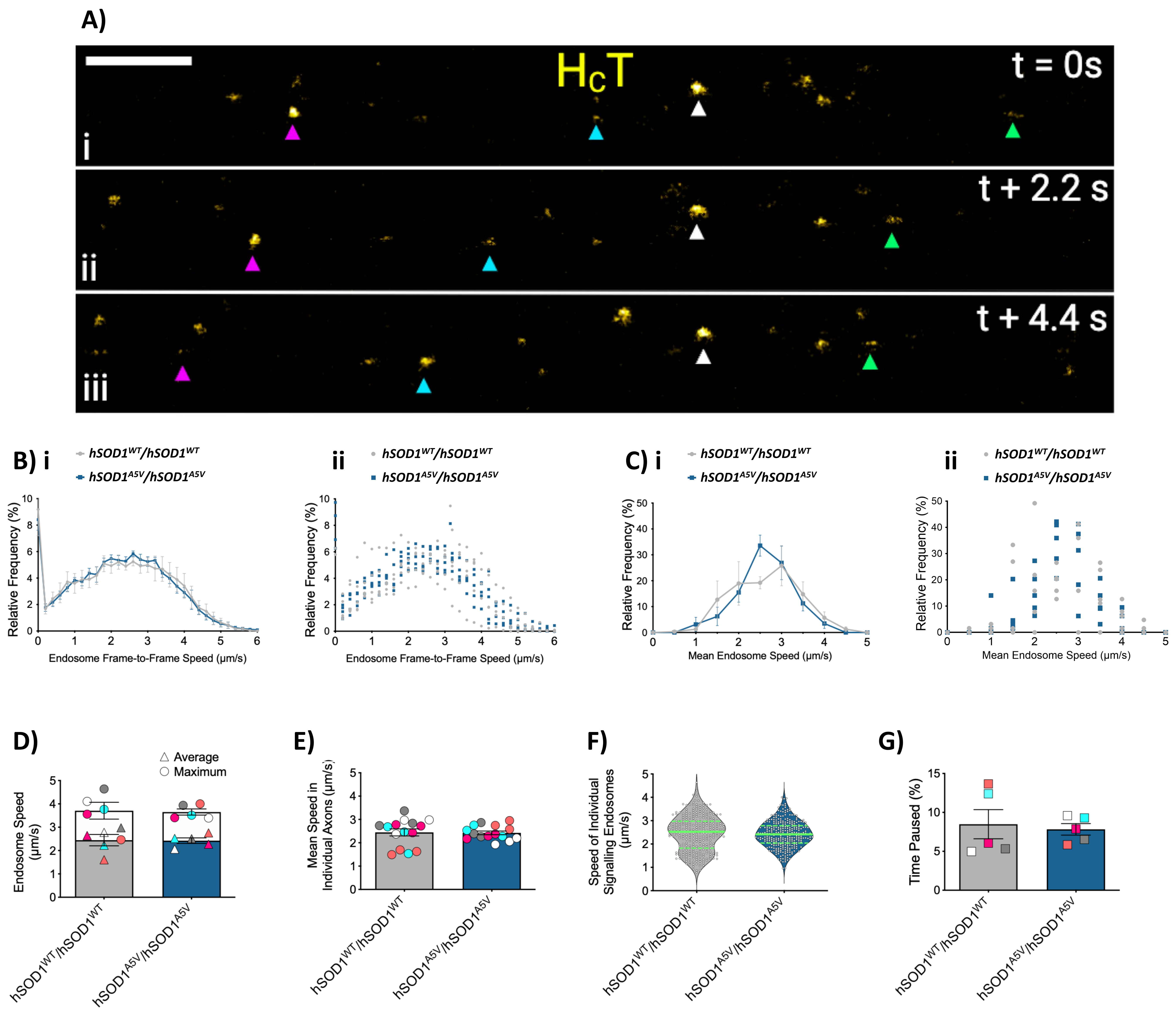

Supplementary Figure 10. Axonal trafficking analysis in male *$hSOD1^{A5V}$* mice. A) Retrogradely transported H_CT-positive endosomes (H_CT - atoxic fragment of tetanus neurotoxin) (pseudo-coloured yellow) from tibialis anterior innervating motor axons are individually tracked to assess their dynamics. Colour-coded arrowheads (cyan, green and magenta) identify three individual endosomes that are undergoing retrograde axonal transport, whereas the white arrowheads identify a stationary/paused endosome. Scale bar = 5µm; t = 2.2 seconds. Male *$hSOD1^{WT}$* and *$hSOD1^{A5V}$* mice displayed no differences in *in vivo* axonal transport dynamics as demonstrated by the B) endosomal frame-to-frame (316-320 endosomes per genotype, from 5 animals per genotype); and C) mean endosome distribution curves (316-320 endosomes per genotype, from 5 animals per genotype); D) average signalling endosome speeds per animal (maximum [circles] $P = 0.55$, mean [triangles] $P = 0.69$, Mann Whitney; 5 animals per genotype; mean ± SEM); E) mean speeds in individual axons ($P = 0.49$, Mann Whitney; 15 axons per genotype, from 5 animals per genotype; mean ± SEM), F) individual signalling endosome speeds (green lines indicate the median and the green dashed lines represent the upper and lower quartiles; 316-320 endosomes per genotype); and G) pausing ($P = 0.84$, Mann Whitney; 5 animals per genotype; mean ± SEM). B, C) inset panels display the same data as i) mean ± SEM and ii) individual datapoints. D, E-F) Individual animal datapoints are represented by single dots (each point = one animal). The colour coding of individual datapoints reflects the same animal in Fig S10D, E and G.

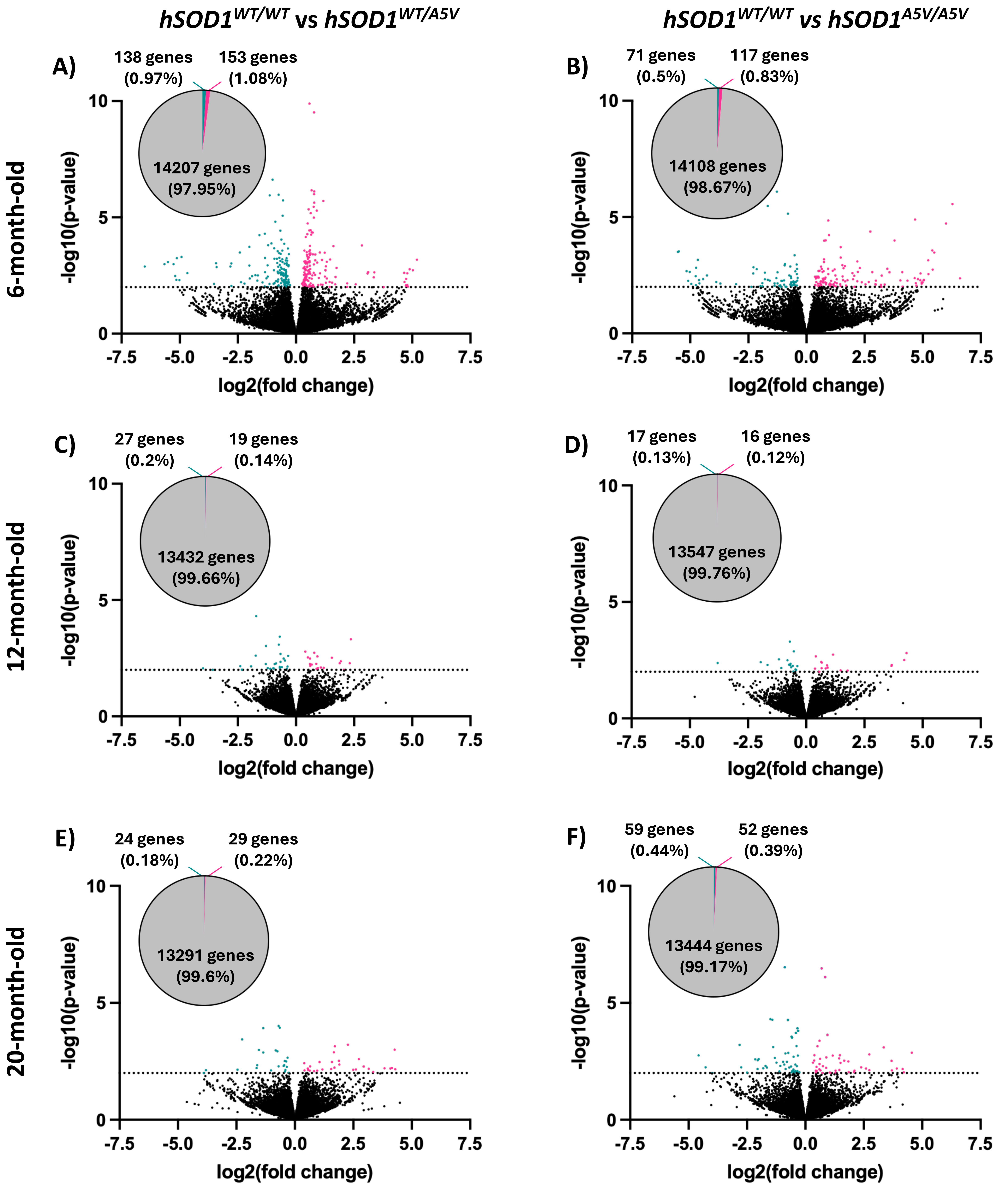

Supplementary Figure 11. Differential gene expression in the tibialis anterior of male *hSOD1^{A5V}* mice compared to *hSOD1^{WT}* controls. Volcano plots showing significantly upregulated genes (magenta) and downregulated genes (teal) (uncorrected *P*-value < 0.01) in the tibialis anterior of male 6-month-old A) *hSOD1$^{WT/A5V}$* and B) *hSOD1$^{A5V/A5V}$*, 12-month-old C) *hSOD1$^{WT/A5V}$* and D) *hSOD1$^{A5V/A5V}$* and 20-month-old E) *hSOD1$^{WT/A5V}$* and F) *hSOD1$^{A5V/A5V}$* animals (4 animals per genotype, per time-point). Non-significant genes with a *P*-value > 0.01 are shown in black. To identify genes considered possibly biologically relevant, analysis was conducted using an uncorrected *P*-value of <0.01. Each datapoint represents a single gene. Venn diagrams display the number of identified genes and the proportion that are significantly up and down regulated. Fig. 4 and Supplementary Fig. 11-18 all relate to transcriptomic data.

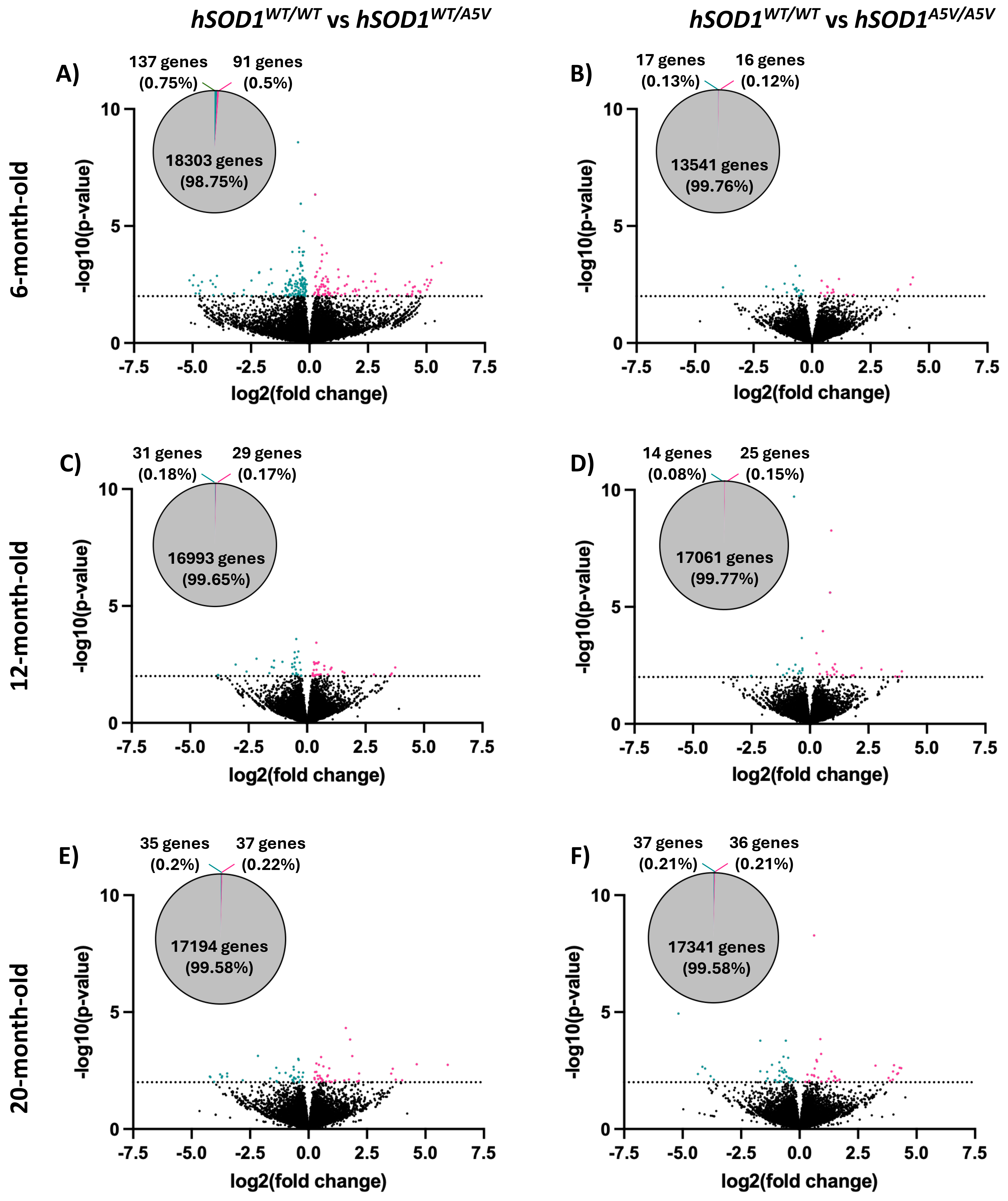

Supplementary Figure 12. Differential gene expression in the spinal cord of male *hSOD1*^{A5V} mice compared to *hSOD1*^{WT} controls. Volcano plots showing significantly upregulated genes (magenta) and downregulated genes (teal) (uncorrected *P*-value < 0.01) in the spinal cord of male 6-month-old A) *hSOD1*$^{WT/A5V}$ and B) *hSOD1*$^{A5V/A5V}$, 12-month-old C) *hSOD1*$^{WT/A5V}$ and D) *hSOD1*$^{A5V/A5V}$ and 20-month-old E) *hSOD1*$^{WT/A5V}$ and F) *hSOD1*$^{A5V/A5V}$ animals (4 animals per genotype, per time-point). Non-significant genes with a *P*-value > 0.01 are shown in black. To identify genes considered possibly biologically relevant, analysis was conducted using an uncorrected *P*-value of <0.01. Each datapoint represents a single gene. Venn diagrams display the number of identified genes and the proportion that are significantly up and down regulated. Fig. 4 and Supplementary Fig. 11-18 all relate to transcriptomic data.

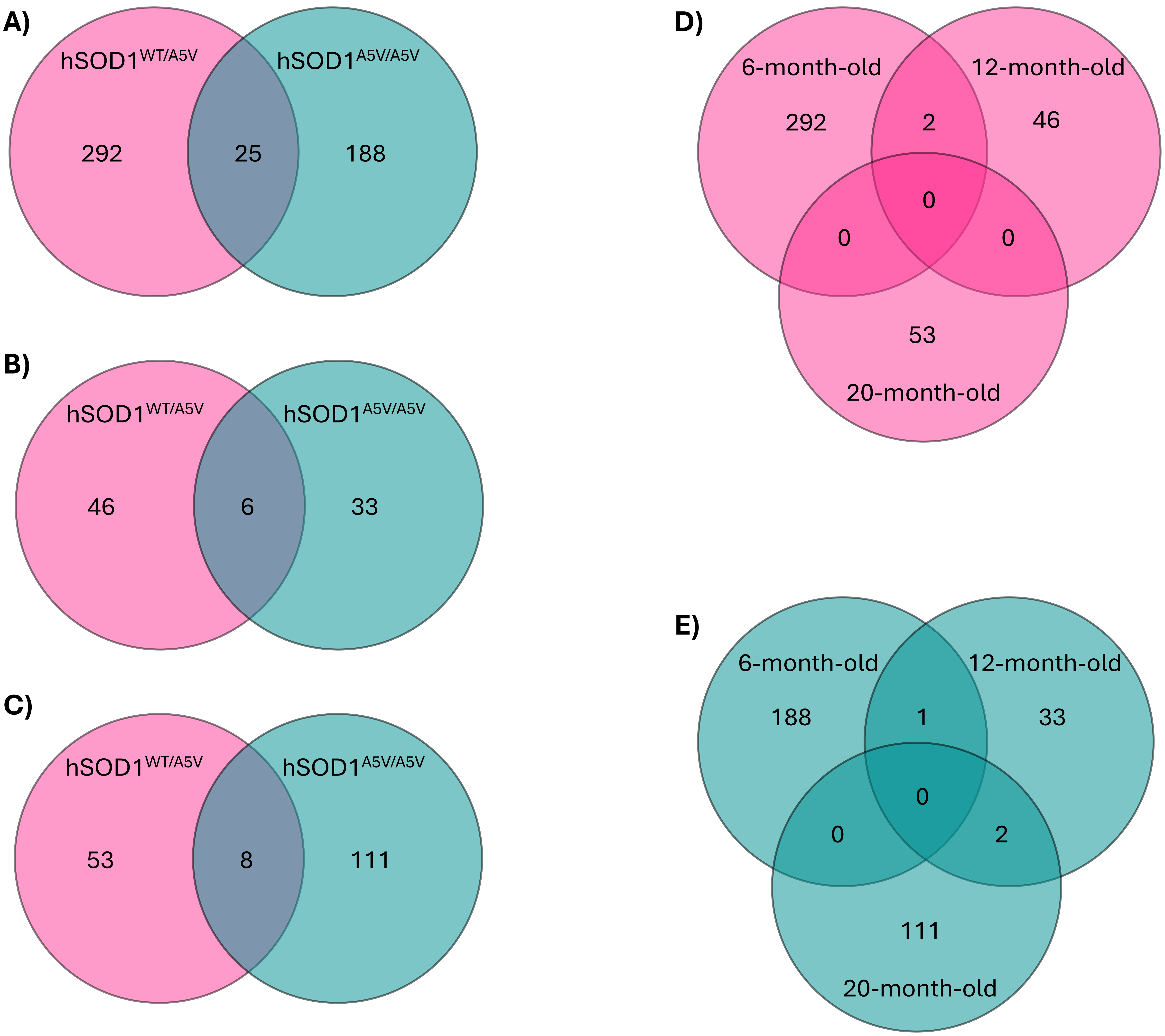

Supplementary Figure 13. Overlap of differentially expressed genes the tibialis anterior of male *hSOD1^{A5V}* mice compared to *hSOD1^{WT}* controls. Venn plots displaying the overlap of common significantly differentially expressed genes ($P < 0.01$) in male *hSOD1$^{WT/A5V}$* (pink) and *hSOD1$^{A5V/A5V}$* (blue) animals at A) 6-month-old B) 12-month-old and C) 20-month-old. D) Common significantly differentially expressed genes ($P < 0.01$) at 6-, 12- and 20- month-old in *hSOD1$^{WT/A5V}$* animals. E) Common significantly differentially expressed genes ($P < 0.01$) at 6-, 12- and 20-month-old in *hSOD1$^{A5V/A5V}$* animals (4 animals per genotype, per time-point). Fig. 4 and Supplementary Fig. 11-18 all relate to transcriptomic data.

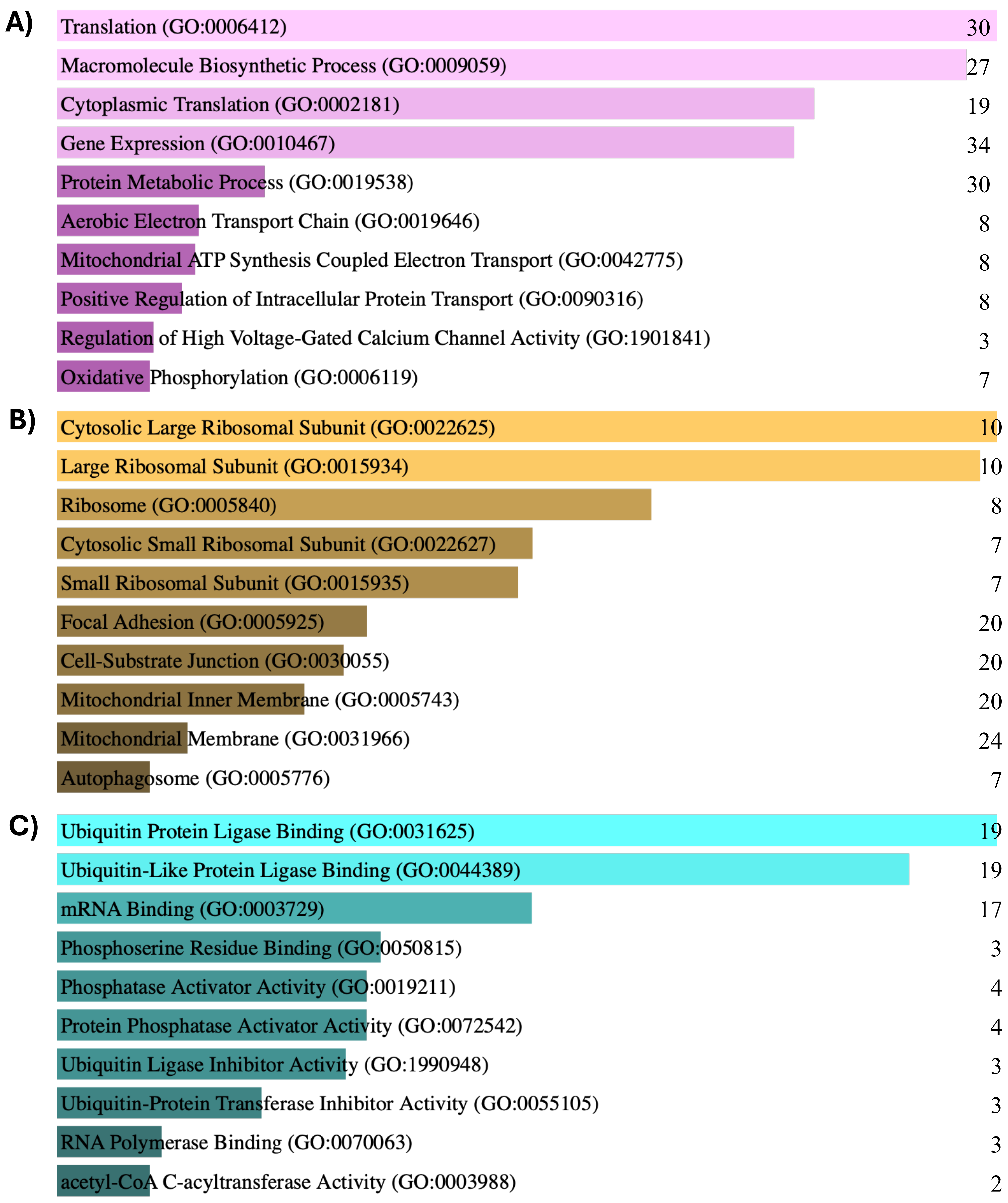

Supplementary Figure 14. Enriched GO terms associated with significantly differentially expressed genes in the tibialis anterior muscle of *hSOD1*$^{WT/A5V}$mice. Analysis using combined up- and downregulated datasets from differentially expression analysis showed biological process (A), cellular components (B) and molecular functions (C), ranked by *P*-value, most affected in male 6-, 12- and 20-month-old *hSOD1*$^{WT/A5V}$ mice (4 animals per genotype, per time-point). The number of differentially expressed genes associated with the respective GO terms is listed to the right of each term. Fig. 4 and Supplementary Fig. 11-18 all relate to transcriptomic data.

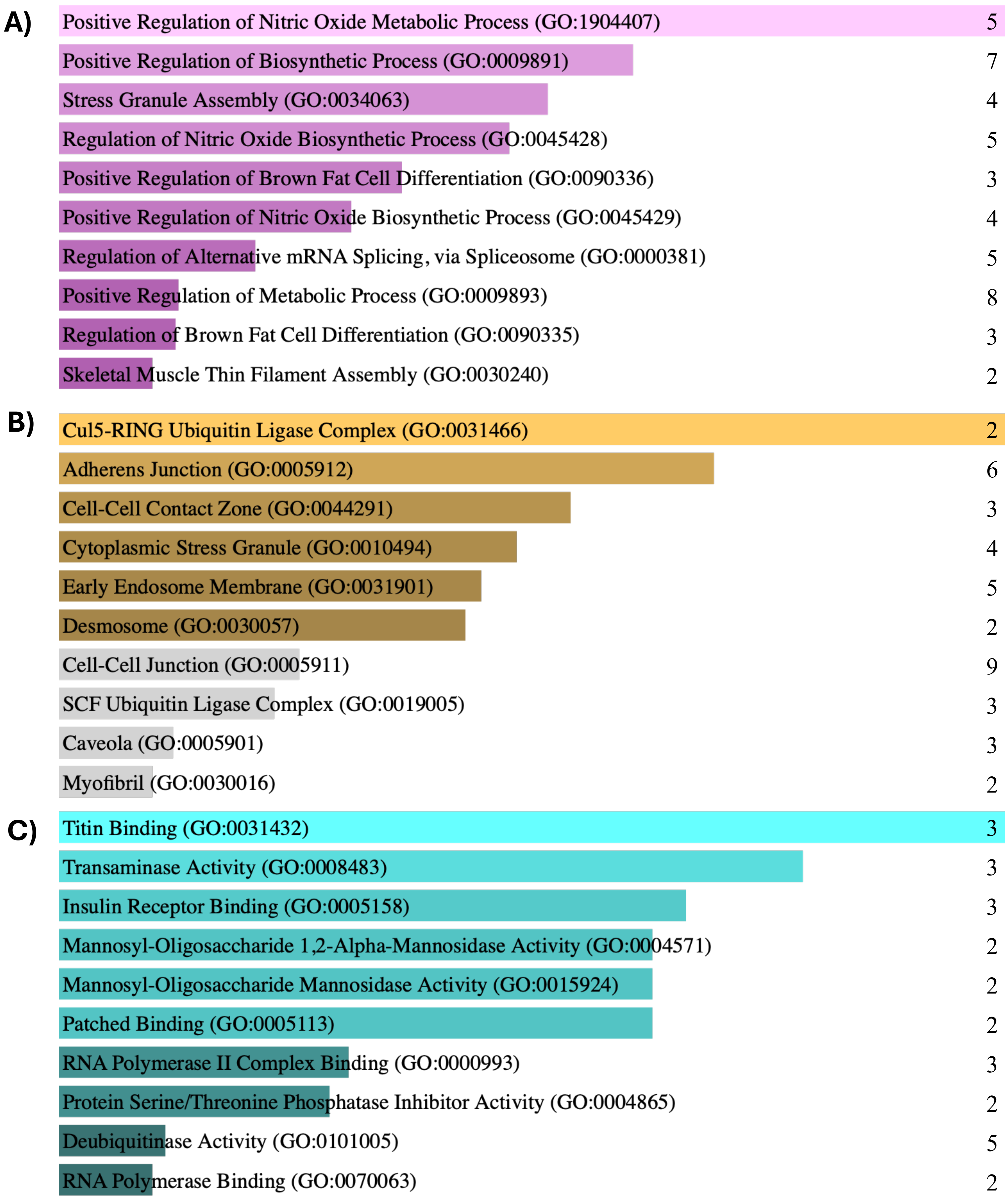

Supplementary Figure 15. Enriched GO terms associated with significantly differentially expressed genes in the tibialis anterior muscle of *hSOD1*$^{A5V/A5V}$ mice. Analysis using combined up- and downregulated datasets from differentially expression analysis showed biological process (A), cellular components (B) and molecular functions (C), ranked by *P*-value, most affected in 6-, 12- and 20-month-old male *hSOD1*$^{A5V/A5V}$ mice (4 animals per genotype, per time-point). The number of differentially expressed genes associated with the respective GO terms is listed to the right of each term. Fig. 4 and Supplementary Fig. 11-18 all relate to transcriptomic data.

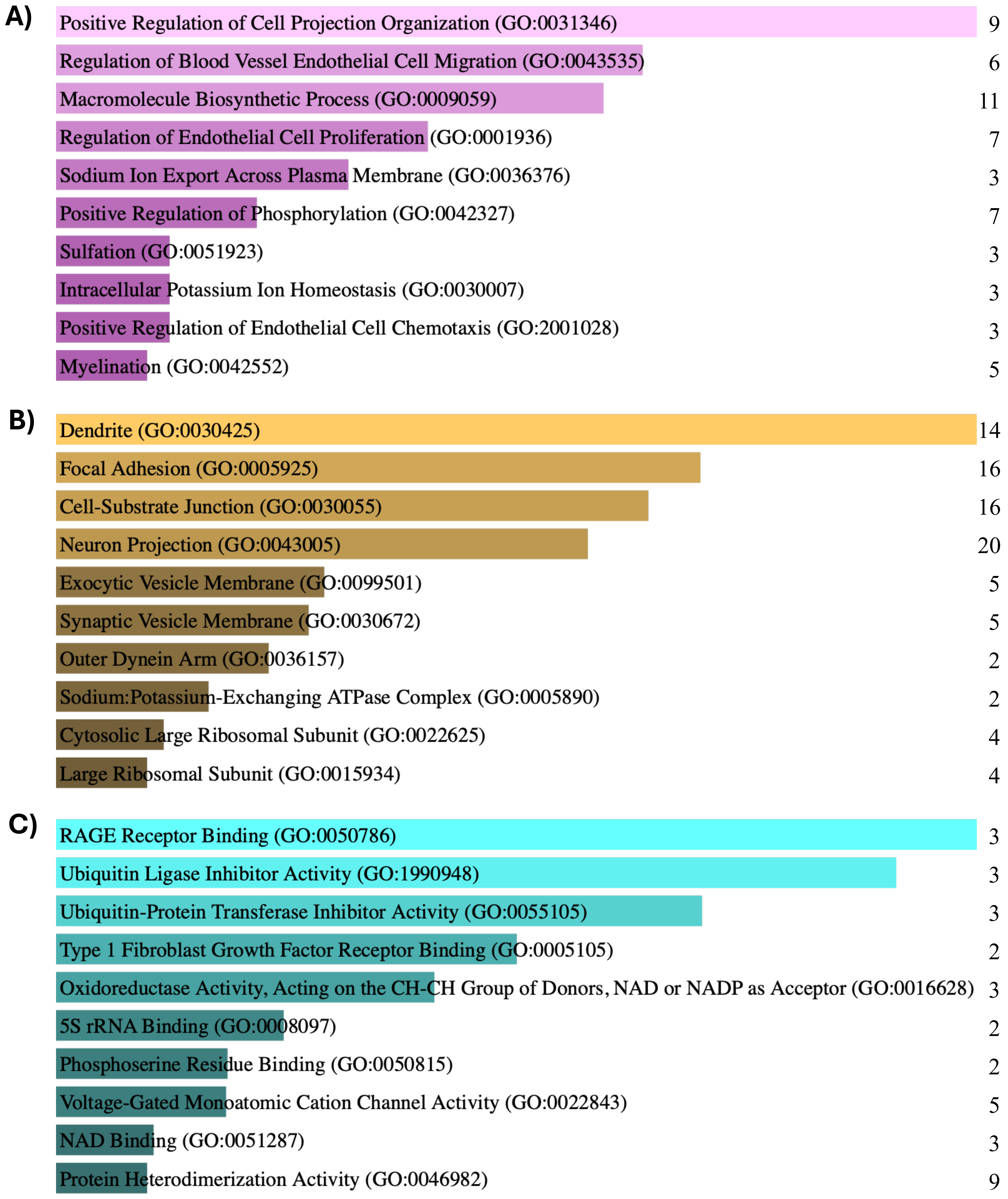

Supplementary Figure 16. Enriched GO terms associated with significantly differentially expressed genes in the spinal cord of *hSOD1*$^{WT/A5V}$mice. Analysis using combined up- and downregulated datasets from differentially expression analysis showed biological process (A), cellular components (B) and molecular functions (C), ranked by *P*-value, most affected in 6-, 12- and 20-month-old male *hSOD1*$^{WT/A5V}$ mice (4 animals per genotype, per time-point). The number of differentially expressed genes associated with the respective GO terms is listed to the right of each term. Fig. 4 and Supplementary Fig. 11-18 all relate to transcriptomic data.

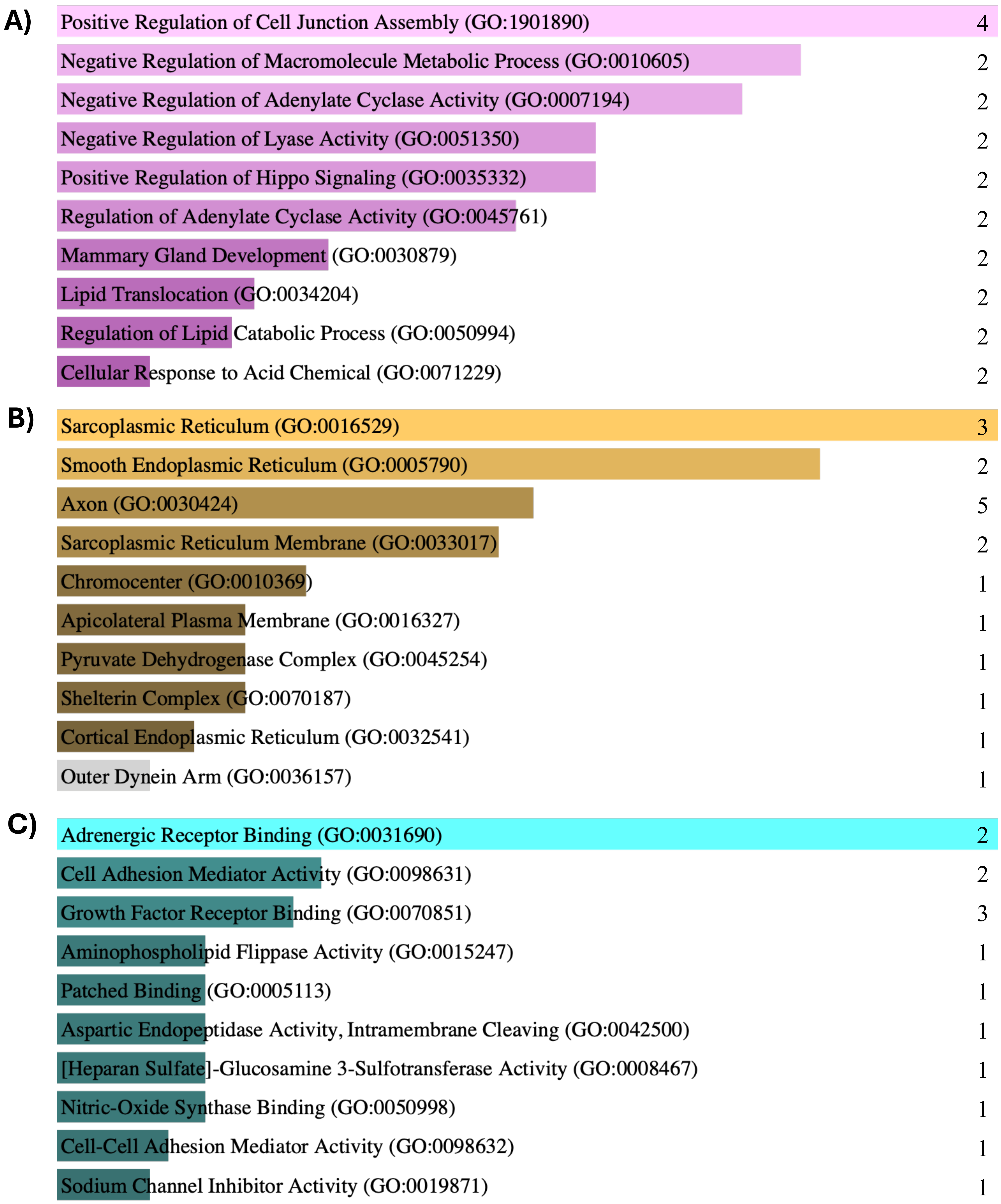

Supplementary Figure 17. Enriched GO terms associated with significantly differentially expressed genes in the spinal cord of *hSOD1*$^{A5V/A5V}$mice. Analysis using combined up- and downregulated datasets from differentially expression analysis showed biological process (A), cellular components (B) and molecular functions (C), ranked by *P*-value, most affected in 6-, 12- and 20-month-old male *hSOD1*$^{A5V/A5V}$ mice (4 animals per genotype, per time-point). The number of differentially expressed genes associated with the respective GO terms is listed to the right of each term. Fig. 4 and Supplementary Fig. 11-18 all relate to transcriptomic data.

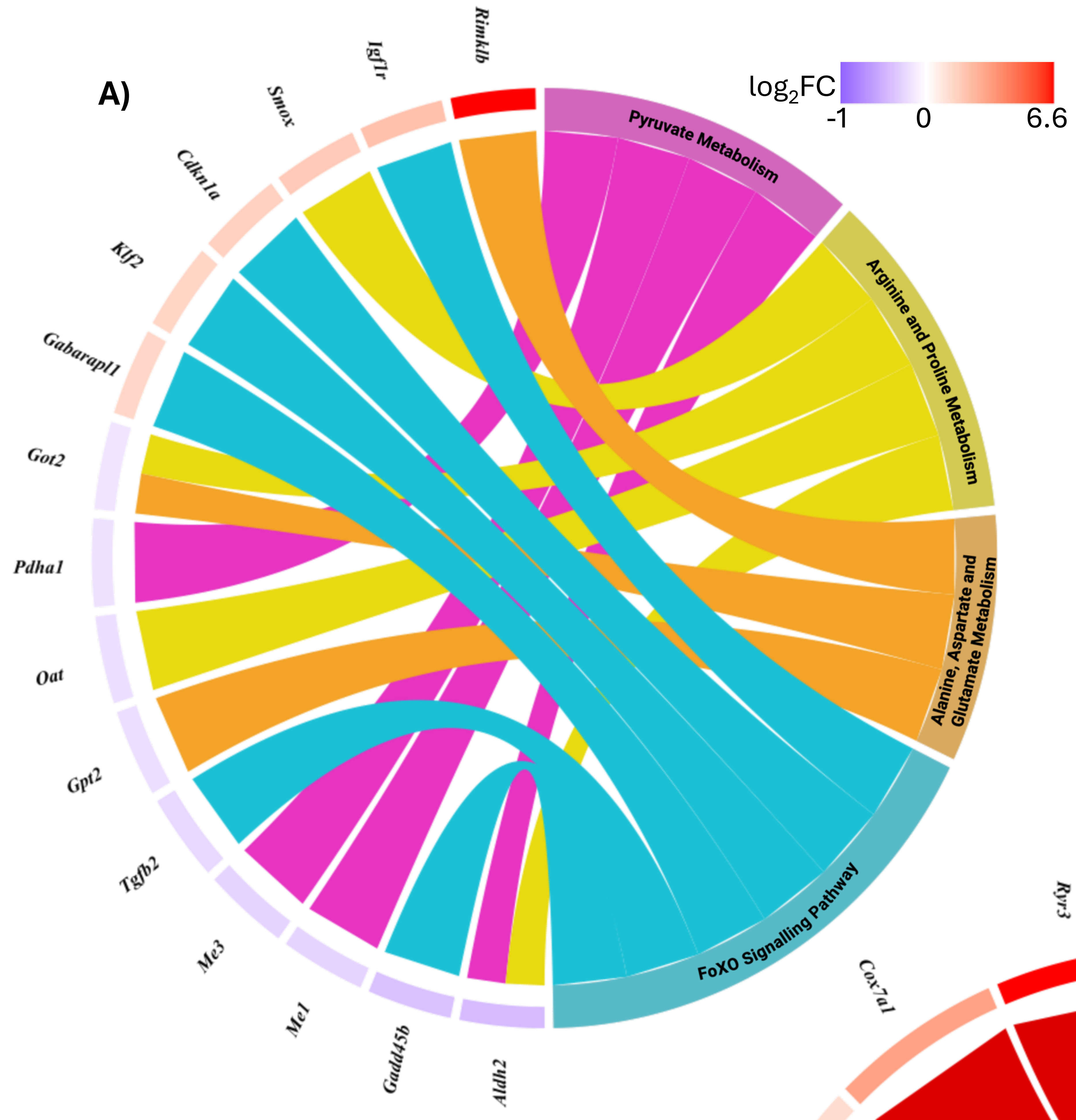

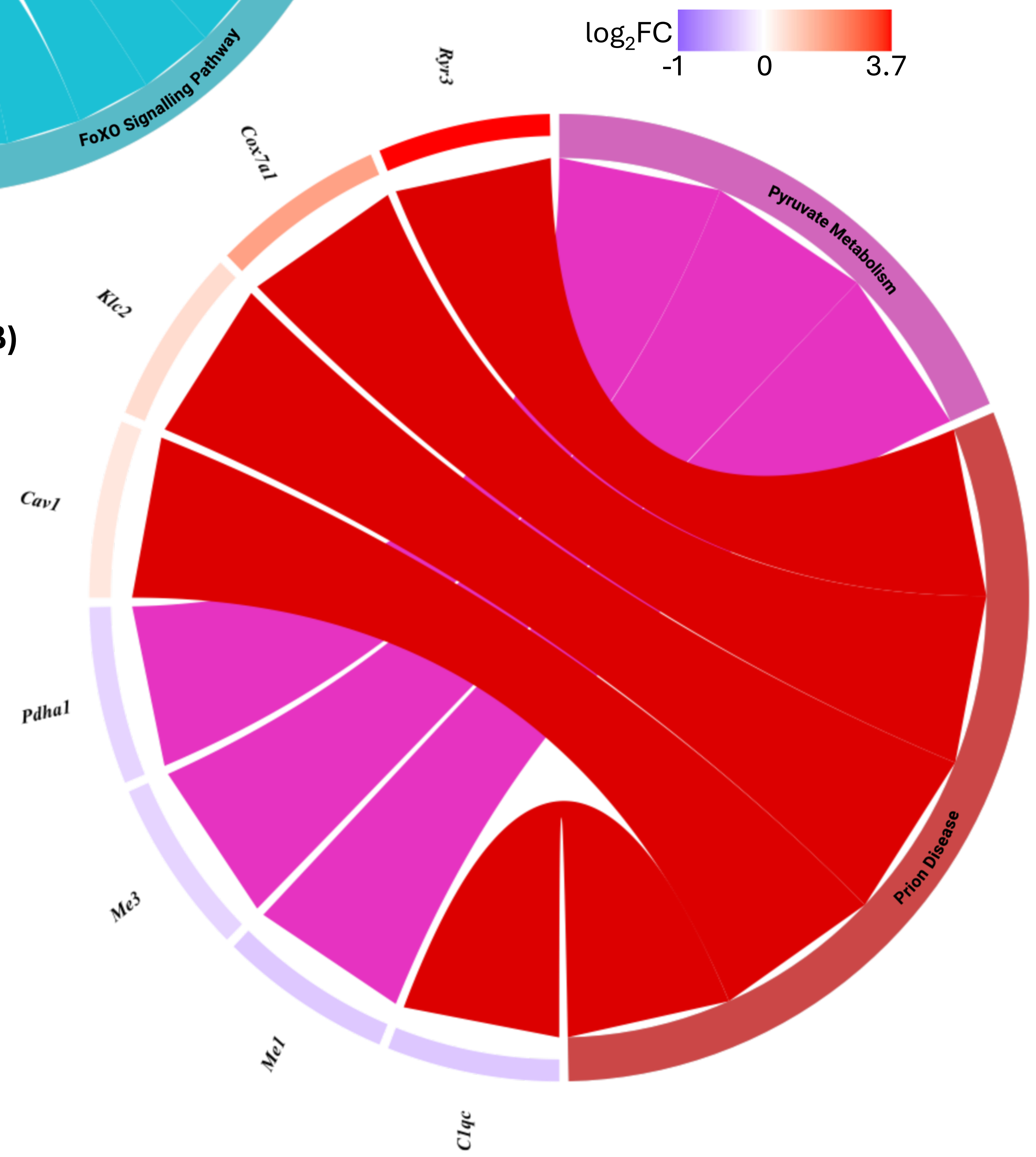

Supplementary Figure 18. Chord diagram showing the core genes of significant affected pathways in *hSOD1$^{WT/A5V}$* mice compared to littermate controls. Significant pathways identified from DEGs in the A) tibialis anterior and B) spinal cord of *hSOD1$^{WT/A5V}$* vs *hSOD1$^{WT/WT}$* of male 6-, 12- and 20-month-old mice. Significant pathways are shown on the right and the fold change of core genes is shown on the left. Left-right connections indicate gene membership in a pathways leading-edge subset. For transcriptomic data *n* =4 male animals per genotype, per timepoint, per tissue. Image constructed using SRPlot[83] and created in BioRender. Price, G. (2026) https://BioRender.com/evz5el1. Fig. 4 and Supplementary Fig. 11-18 all relate to transcriptomic data.

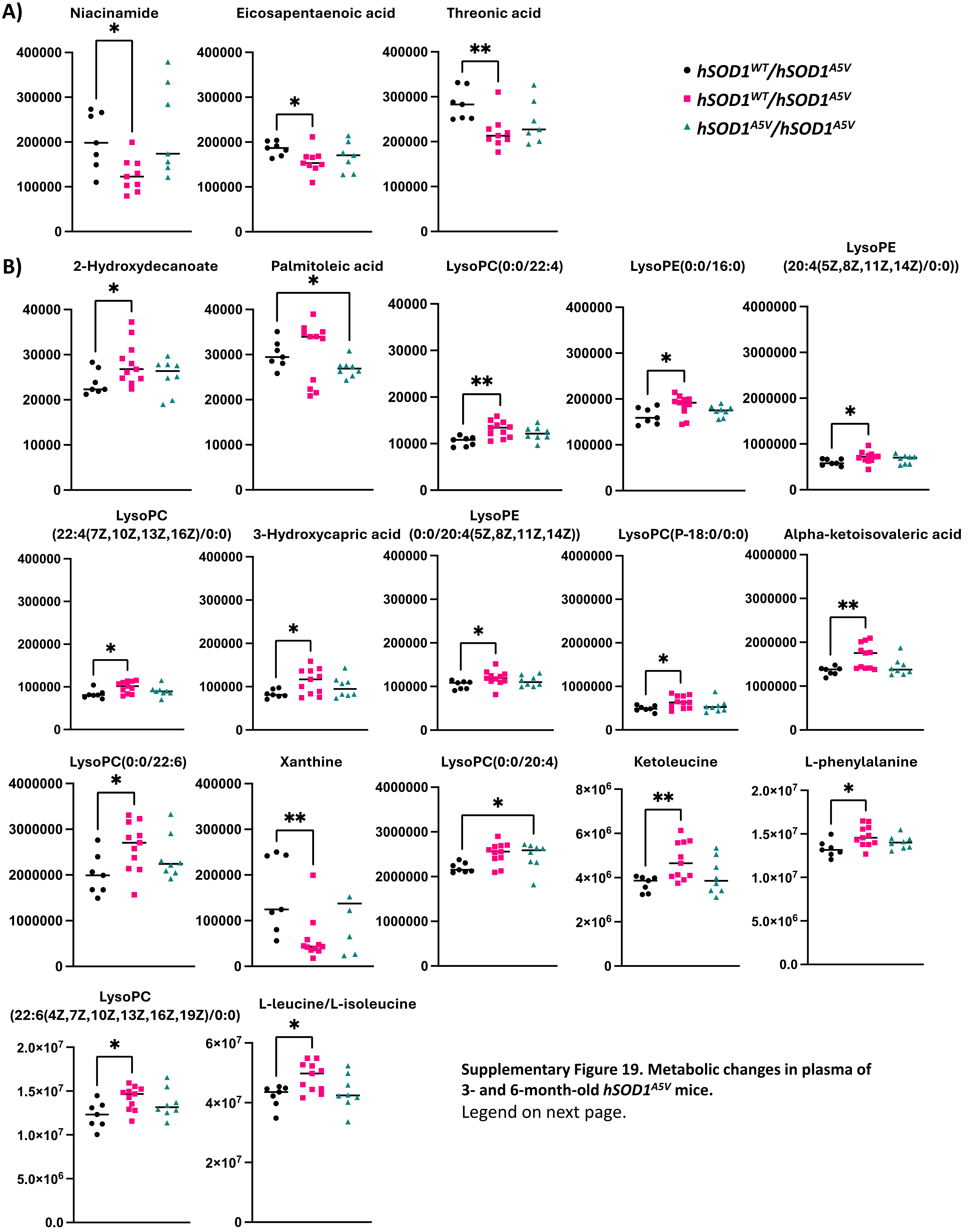

Supplementary Figure 19. Metabolic changes in plasma of 3- and 6-month-old *hSOD1*^{A5V} mice.
Legend on next page.

Supplementary Figure 19. Metabolic changes in plasma of 3- and 6-month-old *hSOD1*^{A5V} mice. Significantly altered metabolites in the blood of male and female A) 3-month-old (n = 3-6 male and n = 3-6 female animals per genotype) and B) 6-month-old (n = 3-6 male and n = 3-6 female animals per genotype) *hSOD1*$^{WT/A5V}$ and *hSOD1*$^{A5V/A5V}$ animals compared to littermate controls. Each data point represents an individual animal. One-way ANOVA with Tukey's post hoc test. Main effects: A) niacinamide $F(2, 20) = 5.423$, $P = 0.0131$; eicosapentaenoic acid $F(2, 20) = 2.322$, $P = 0.1240$; threonic acid $F(2, 20) = 5.029$, $P = 0.0170$; B) 2-hydroxydecanoate $F(2, 23) = 2.539$, $P = 0.1009$; palmitoleic acid $F(2, 23) = 1.039$, $P = 0.3699$; LysoPC(0:0/22:4) $F(2, 23) = 6.7$, $P = 0.0051$; LysoPE(0:0/16:0) $F(2, 23) = 3$, $P = 0.0677$; LysoPE(20:4(5Z,8Z,11Z,14Z)/0:0)) $F(2, 23) = 1.045$, $P = 0.0368$; LysoPC(22:4(7Z,10Z,13Z,16Z)/0:0)) $F(2, 23) = 3.881$, $P = 0.0353$; 3-hydroxycapric acid $F(2, 23) = 3.044$, $P = 0.0672$; LysoPE(0:0/20:4(5Z,8Z,11Z,14Z)) $F(2, 23) = 2.521$, $P = 0.1024$; LysoPC(P-18:0/0:0) $F(2, 23) = 3.349$, $P = 0.0529$; alpha-ketoisovaleric acid $F(2, 23) = 5.787$, $P = 0.0092$; LysoPC(0:0/22:6) $F(2, 23) = 3.394$, $P = 0.0511$; xanthine $F(2, 23) = 4.261$, $P = 0.0267$; LysoPC(0:0/20:4) $F(2, 23) = 3.737$, $P = 0.0393$; ketoleucine $F(2, 23) = 3.737$, $P = 0.0393$; L-phenylalanine $F(2, 23) = 4.498$, $P = 0.0225$; LysoPC(22:6(4Z,7Z,10Z,13Z,16Z,19Z)/0:0) $F(2, 23) = 3.822$, $P = 0.0369$; L-leucine/L-isoleucine $F(2, 23) = 4.215$, $P = 0.0276$. $^{*}P < 0.05$, $^{**}P < 0.01$.

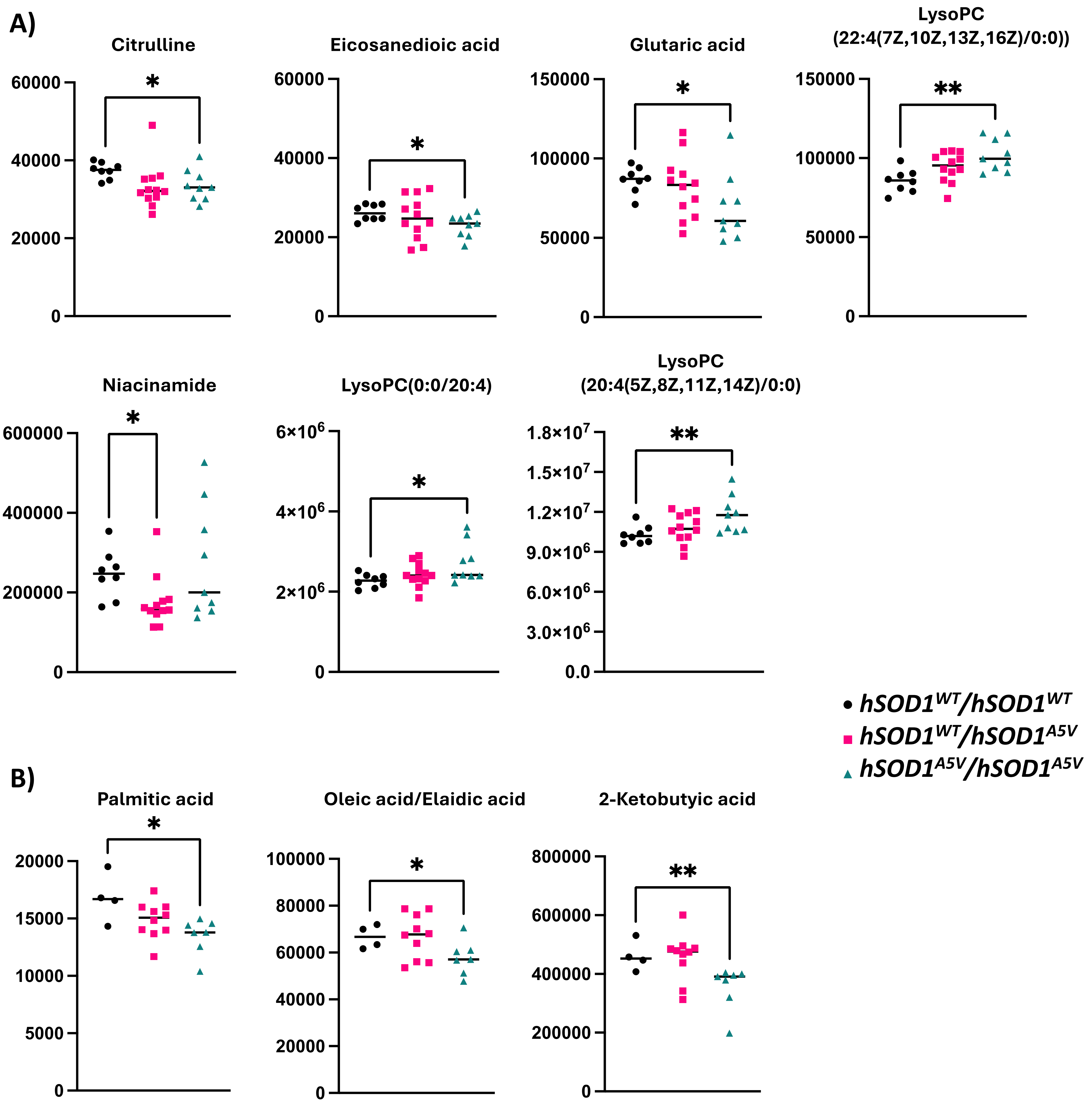

Supplementary Figure 20. Metabolic changes in plasma of 9 and 12-month-old *hSOD1*^{A5V} mice. Significantly altered metabolites in the blood of male and female A) 9-month-old (*n* = 3-6 male and *n* = 4-6 female animals per genotype) and B) 12-month-old (*n* = 3-6 male and *n* = 2-6 female animals per genotype) *hSOD1*$^{WT/A5V}$ and *hSOD1*$^{A5V/A5V}$ animals compared to littermate controls. Each data point represents an individual animal. One-way ANOVA with Tukey's post hoc test. Main effects: A) citrulline $F(2, 26) = 2.828$, $P = 0.0774$; eicosanedioic acid $F(2, 26) = 1.416$, $P = 0.2609$; glutaric acid $F(2, 26) = 3.033$, $P = 0.0654$; LysoPC(22:4(7Z,10Z,13Z,16Z(/0:0)) $F(2, 26) = 6.824$, $P = 0.0041$; niacinamide $F(2, 26) = 3.402$, $P = 0.0487$; LysoPC(0:0/20:4) $F(2, 26) = 3.737$, $P = 0.0377$; LysoPC(20:4(5Z,8Z,11Z,14Z)/0:0) $F(2, 26) = 4.132$, $P = 0.0277$; B) palmitic acid $F(2, 18) = 4.436$, $P = 0.0271$; oleic acid/elaidic acid $F(2, 18) = 2.924$, $P = 0.0795$; 2-ketobutyric acid $F(2, 18) = 3.985$, $P = 0.0369$. $^{*}P < 0.05$, $^{**}P < 0.01$.

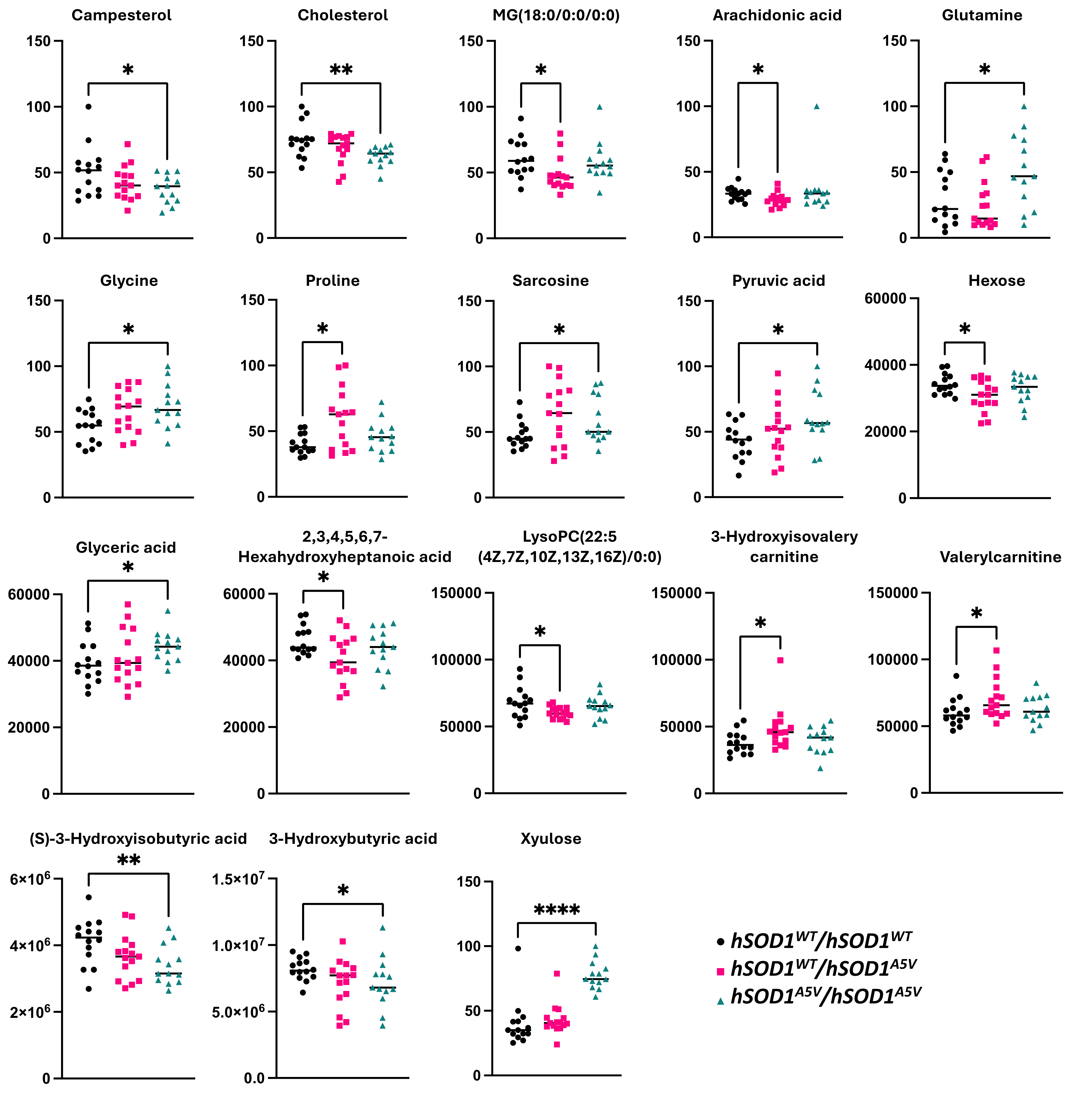

Supplementary Figure 21. Metabolic changes in plasma of 20-month-old *$hSOD1^{A5V}$* mice. Significantly altered metabolites in the blood of male and female 20-month-old *$hSOD1^{WT/A5V}$* and *$hSOD1^{A5V/A5V}$* animals compared to littermate controls. n = 7-8 male and n = 6-7 female animals per genotype. Each data point represents an individual animal. One-way ANOVA with Tukey's post hoc test. Main effects: campesterol $F(2, 39) = 2.758$, $P = 0.0758$; cholesterol $F(2, 39) = 3.670$, $P = 0.0347$; MG(18:0/0:0) $F(2, 39) = 3.815$, $P = 0.0307$; arachidonic acid $F(2, 39) = 2.220$, $P = 0.1221$; glutamine $F(2, 39) = 4.272$, $P = 0.0210$; glycine $F(2, 39) = 3.886$, $P = 0.0294$; proline $F(2, 39) = 4.922$, $P = 0.0124$; sarcosine $F(2, 39) = 2.361$, $P = 0.1077$; pyruvic acid $F(2, 39) = 2.519$, $P = 0.0936$; hexose $F(2, 39) = 3.380$, $P = 0.0443$; glyceric acid $F(2, 39) = 2.310$, $P = 0.1127$; 2,3,4,5,6,7-hexahydroxyheptanoic acid $F(2, 29) = 3.487$, $P = 0.0404$; lysoPC(22:5(4Z,7Z,10Z,13Z,16Z)/0:0) $F(2, 39) = 2.787$, $P = 0.0739$; 3-hydroxyisovalerycarnitine $F(2, 39) = 3.279$, $P = 0.0483$; valerylcarnitine $F(2, 39) = 2.624$, $P = 0.0853$; (S)-3-hydroxyisobutyric acid $F(2, 39) = 3.970$, $P = 0.0270$; 3-hydroxybutyric acid $F(2, 29) = 2.295$, $P = 0.1142$; xylose $F(2, 39) = 30.42$, $P < 0.0001$. $^{*}P < 0.05$, $^{**}P < 0.01$, $^{****}P < 0.0001$.

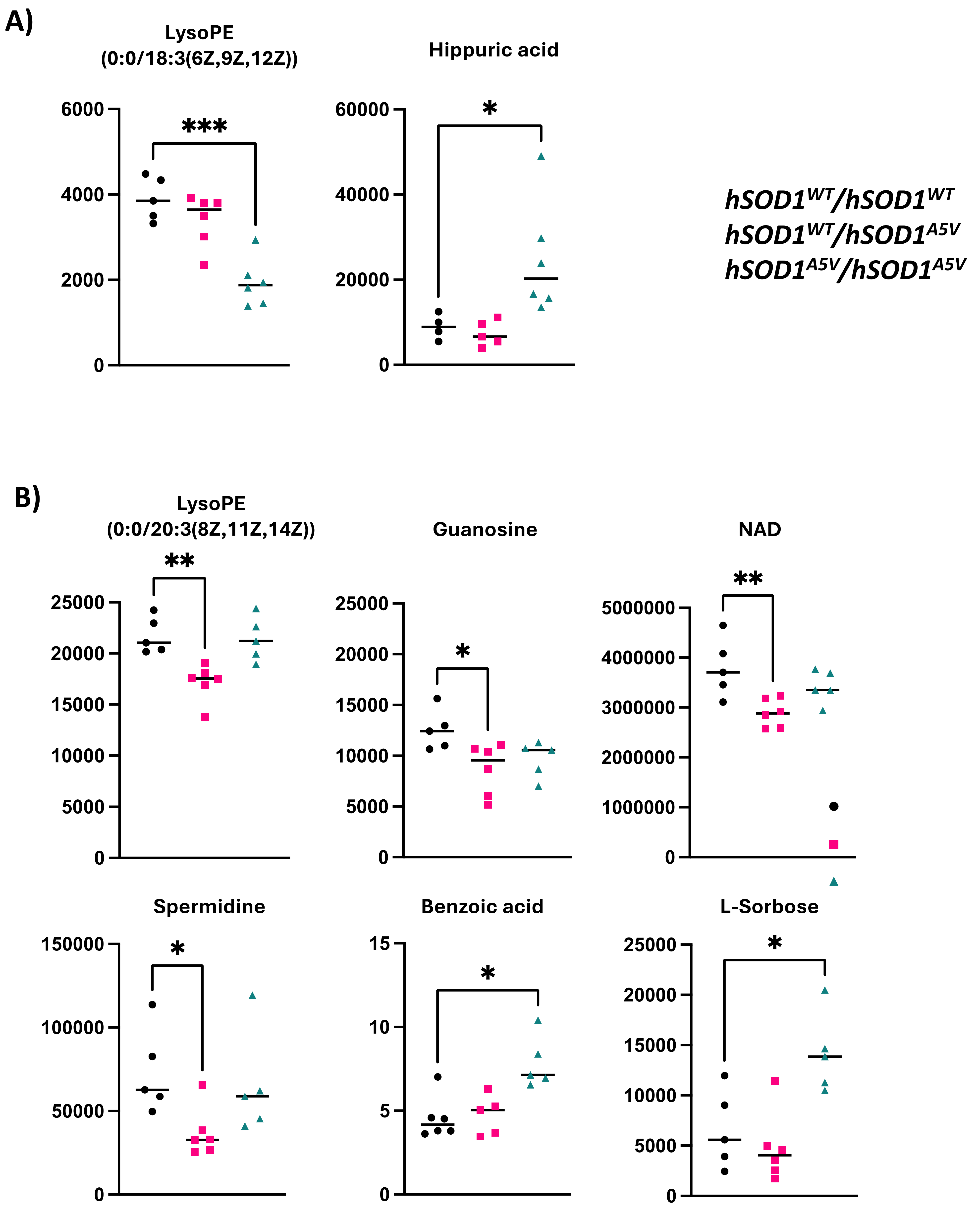

Supplementary Figure 22. Metabolic changes in the spinal cord and tibialis anterior of 20-month-old *hSOD1*^{A5V} mice. Significantly altered metabolites in the A) spinal cord and B) tibialis anterior of male 20-month-old *hSOD1*$^{WT/A5V}$ and *hSOD1*$^{A5V/A5V}$ animals compared to littermate controls. n = 5-6 animals per genotype per tissue. Each data point represents an individual animal. One-way ANOVA with Tukey's post hoc test. Main effects: A) lysoPE(0:0/18:3(6Z,9Z,12Z)) $F(2, 14) = 18.25$, $P = 0.0001$; hippuric acid $F(2, 12) = 11.58$, $P = 0.0016$; B) lysoPE(0:0/20:3(8Z,11Z,14Z)) $F(2, 13) = 10.14$, $P = 0.0022$; guanosine $F(2, 13) = 3.710$, $P = 0.0531$; NAD $F(2, 13) = 7.231$, $P = 0.0077$; spermidine $F(2, 13) = 5.762$, $P = 0.0162$; benzoic acid $F(2, 13) = 9.504$, $P = 0.0029$; L-sorbose $F(2, 13) = 7.090$, $P = 0.0083$. *$P < 0.05$, **$P < 0.01$, ***$P < 0.001$.

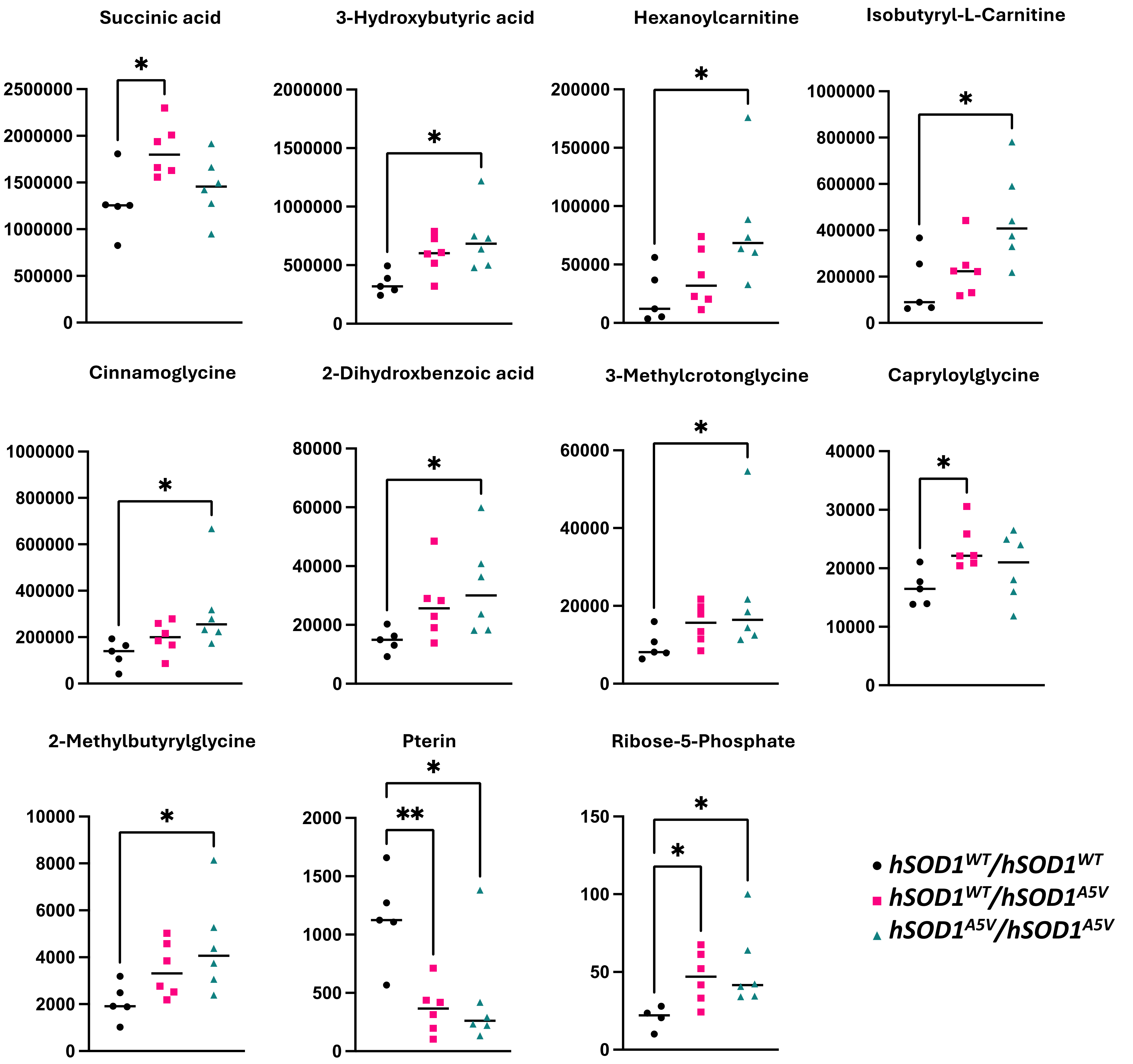

Supplementary Figure 23. Metabolic changes in the liver of 20-month-old *hSOD1^A5V^* mice. Significantly altered metabolites in the liver of male 20-month-old *hSOD1^WT/A5V^* and *hSOD1^A5V/A5V^* animals compared to littermate controls. *n* = 5-6 animals per genotype. Each data point represents an individual animal. One-way ANOVA with Tukey's post hoc test. Main effects: succinic acid $F(2, 14) = 4.291$, $P = 0.0352$; 3-hydroxybutyric acid $F(2, 14) = 7.320$, $P = 0.0067$; hexanoylcarnitine $F(2, 14) = 5.443$, $P = 0.0178$, isobutyryl-L-carnitine $F(2, 14) = 5.719$, $P = 0.0153$; cinnamoglycine $F(2, 14) = 4.593$, $P = 0.0293$; 2-dihydroxbenzoic acid $F(2, 14) = 4.561$, $P = 0.0298$; 3-methylcrotonglycine $F(2, 14) = 3.414$, $P = 0.0620$; capryloylglycine $F(2, 14) = 3.328$, $P = 0.0657$; 2-methylbutyrylglycine $F(2, 14) = 5.009$, $P = 0.0229$; pterin $F(2, 14) = 6.110$, $P = 0.0124$; ribose-5-phosphate $F(2, 13) = 6.702$, $P = 0.01$. $^{*}P < 0.05$, $^{**}P < 0.01$.

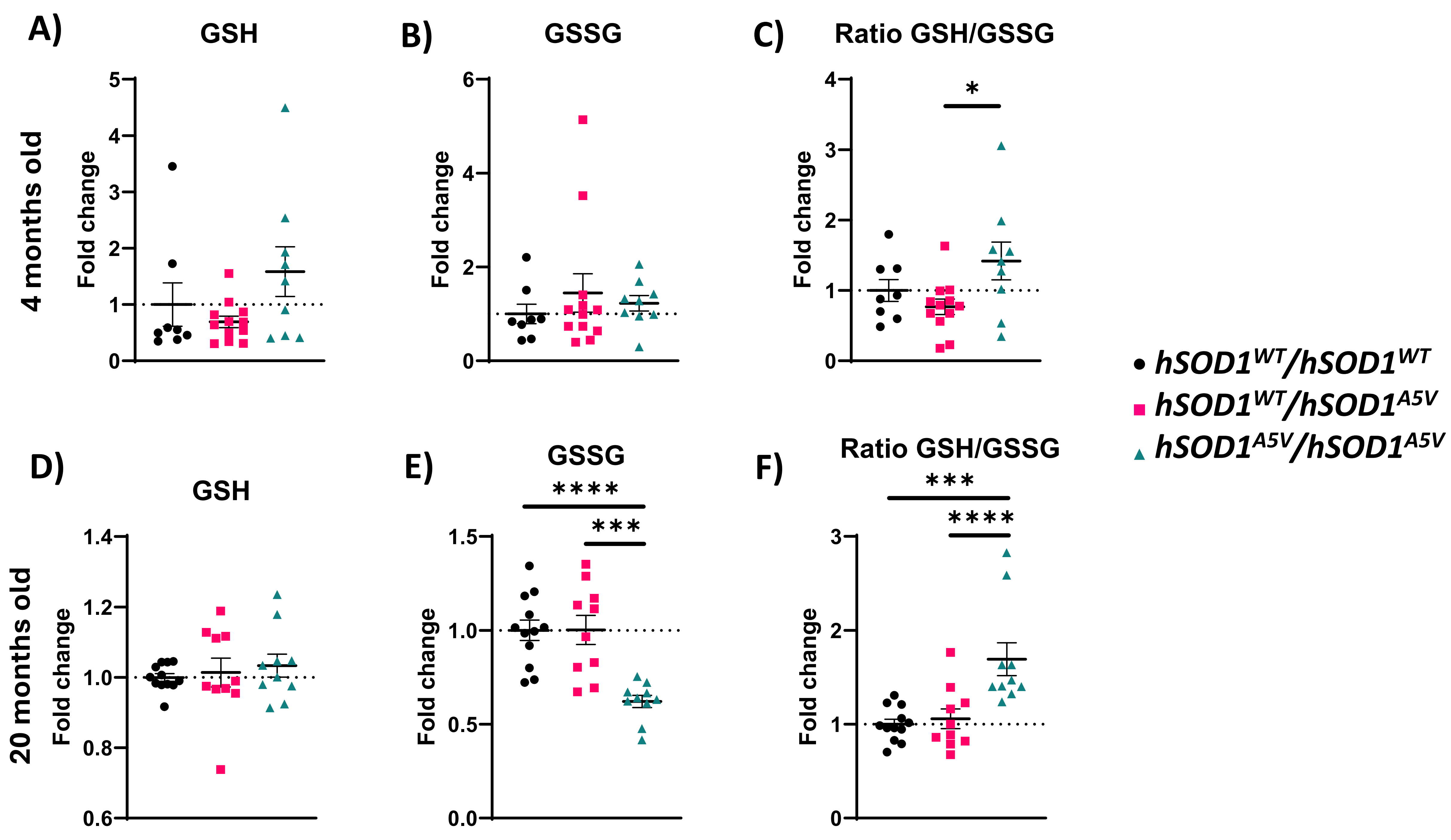

Supplementary Figure 24. Blood glutathione measurements in *hSOD1*^{A5V} mice. There was no significant difference in GSH/GSSG levels between the sexes, hence sexes were pooled for analysis. (A-C) Blood glutathione measurements in 4-month-old *hSOD1*$^{A5V/A5V}$ animals. Data are displayed as fold change relative to *hSOD1*$^{WT/WT}$ animals. n = 3-6 male and n = 4-6 female animals per genotype. One-way ANOVA with Tukey's post hoc test. Main effects: A) $F(2,26) = 2.26$, $P = 0.125$; B) $F(2,26) = 0.475$, $P = 0.627$; C) $F(2,26) = 3.52$, $P = 0.0444$. (D-F) Blood glutathione measurements in 20-month-old *hSOD1*$^{A5V/A5V}$ animals. Data are displayed as fold change relative to *hSOD1*$^{WT/WT}$ animals. 20-month-old data was pooled from two cohorts where each animal is displayed as fold change relative to *hSOD1*$^{WT/WT}$ animals from the same cohort. n = 5-6 male and n = 4-6 female animals per genotype. Two-way ANOVA with cohort and genotype as factors followed by Tukey's post hoc test. Genotype effects: D) $F(2,26) = 1.66$, $P = 0.209$; E) $F(2,26) = 15.1$, $P = 4.37 \times 10^{-5}$; F) $F(2,26) = 21.0$, $P = 3.69 \times 10^{-6}$. Mean ± SEM. *$P < 0.05$, **$P < 0.01$, ****$P < 0.0001$.

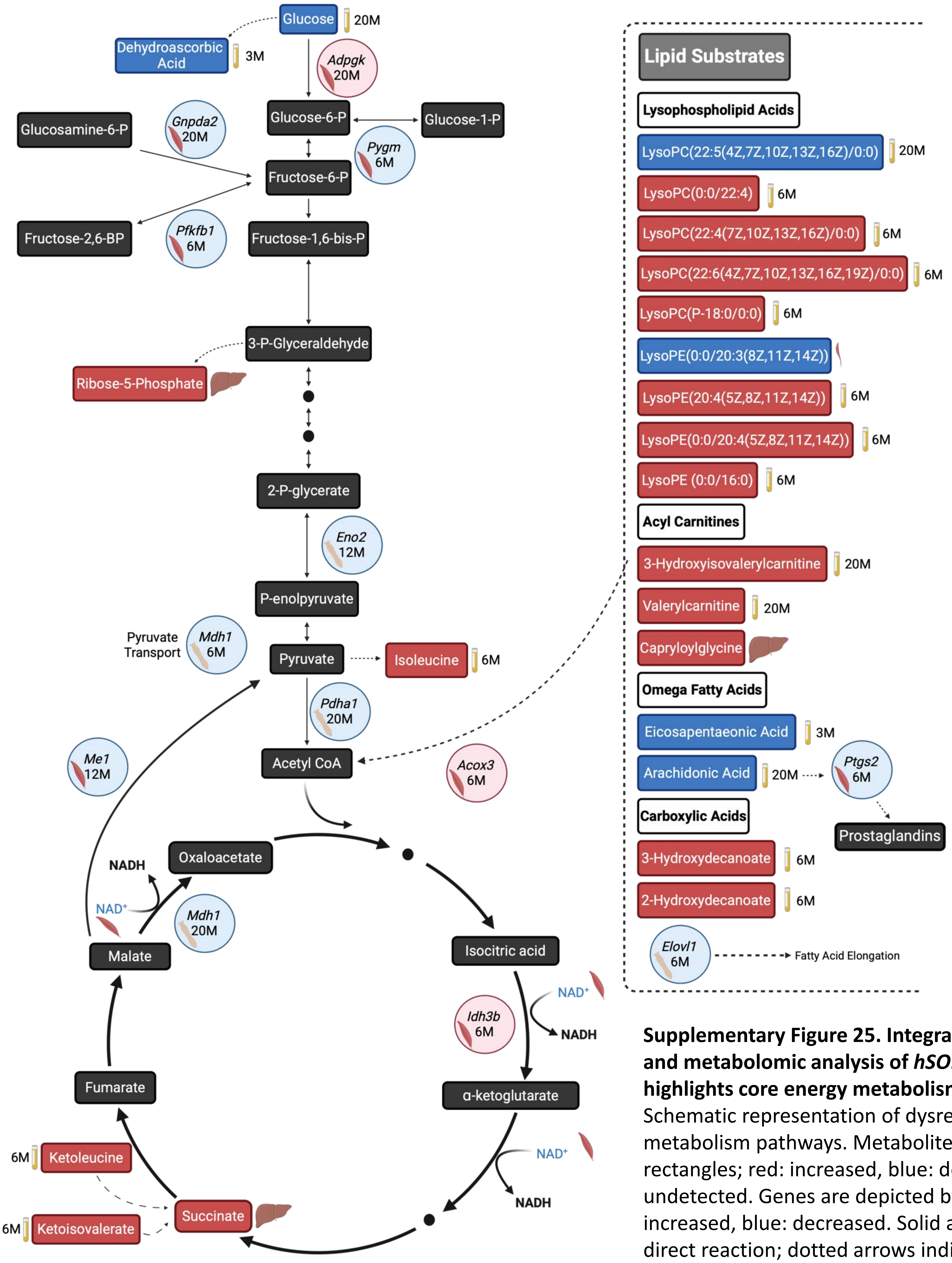

Supplementary Figure 25. Integrated transcriptomic and metabolomic analysis of *hSOD1*$^{WT/A5V}$ mice highlights core energy metabolism pathways. Schematic representation of dysregulated energy metabolism pathways. Metabolites are depicted by rectangles; red: increased, blue: decreased and black: undetected. Genes are depicted by circles; red: increased, blue: decreased. Solid arrows indicate a direct reaction; dotted arrows indicate the reaction is broken by 1 or more sub-reactions. Tissues in which genes and metabolites were found to be significantly altered are indicated by the presence of tibialis anterior, spinal cord, or liver images. The timepoint at which the gene or metabolite was detected as significantly changed is indicated beside the corresponding rectangle or circle. For transcriptomic data *n* = 4 male animals per genotype, per timepoint, per tissue. For metabolomics data *n* = 5-15 animals per genotype, per timepoint per tissue (see Supp. Figs S19-24 for a breakdown of sexes used). Created in BioRender. Price, G. (2026) https://BioRender.com/z2we1h7. Relates to Fig. 5.

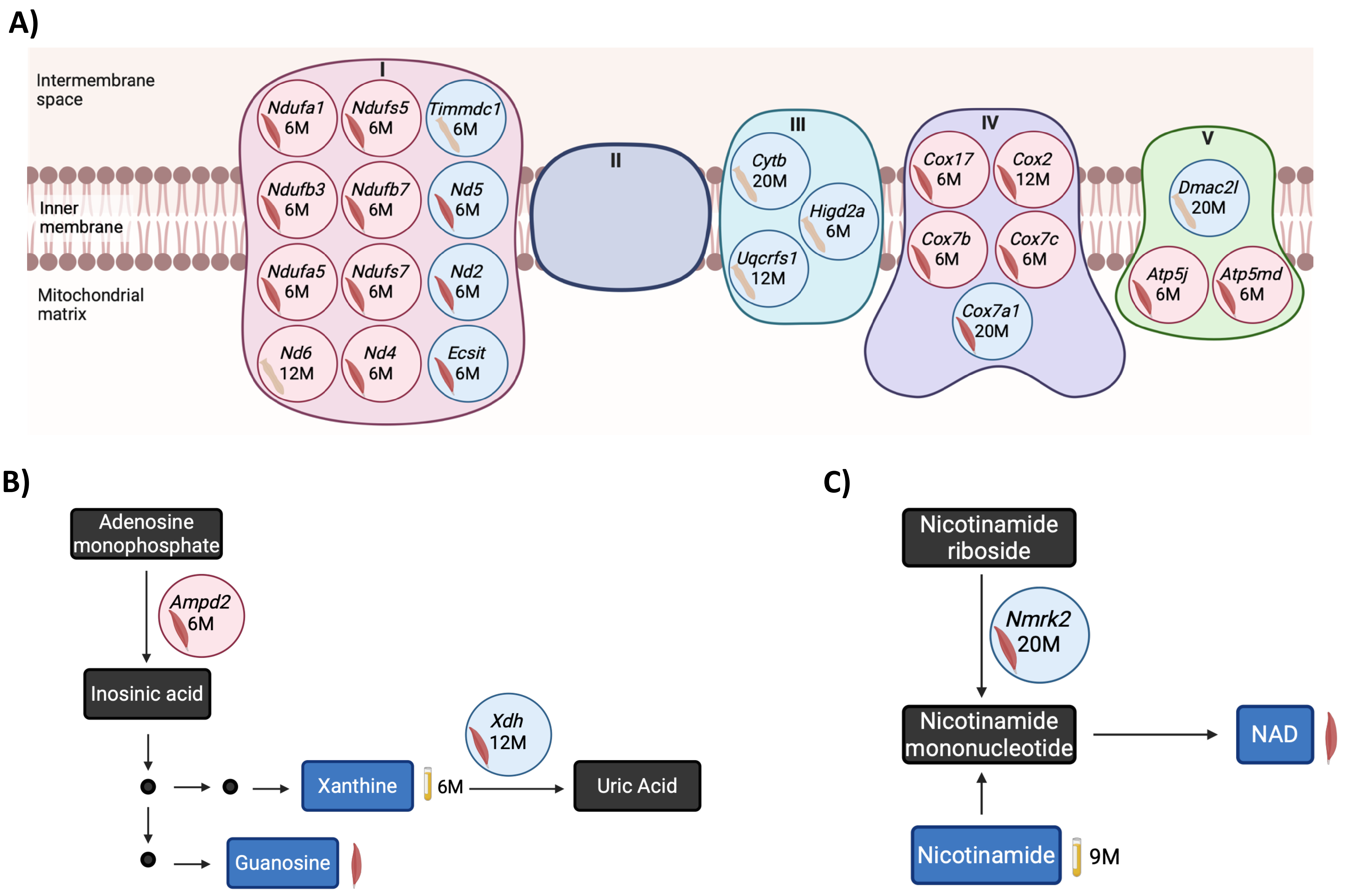

Supplementary Figure 26. Integrated transcriptomic and metabolomic analysis of *hSOD1*$^{WT/A5V}$ mice highlights mitochondrial, xanthine and nicotinamide metabolic dysregulation. Schematic representation of dysregulated pathways, A) mitochondrial function, B) xanthine and C) NAD metabolism, in *hSOD1*$^{WT/A5V}$ mice. Metabolites are depicted in rectangles, red: increased, blue: decreased and black: undetected. Genes are depicted in circle, red: increased, blue: decreased. Solid arrows indicate a direct reaction; dotted arrows indicate the reaction is broken by 1 or more sub-reactions. The tissues in which genes and metabolites were detected to be significantly altered are indicated by the presence of tibialis anterior, spinal cord, or liver images. The timepoint at which the gene or metabolite was detected as significantly changed is indicated beside the corresponding rectangle or circle. For transcriptomic data *n* = 4 male animals per genotype, per timepoint, per tissue. For metabolomics data *n* = 5-15 animals per genotype, per timepoint per tissue (see Supp. Figs S19-24 for a breakdown of sexes used). Image created in Biorender. Price, G. (2026) https://BioRender.com/nulnj8k. Relates to Fig. 5.

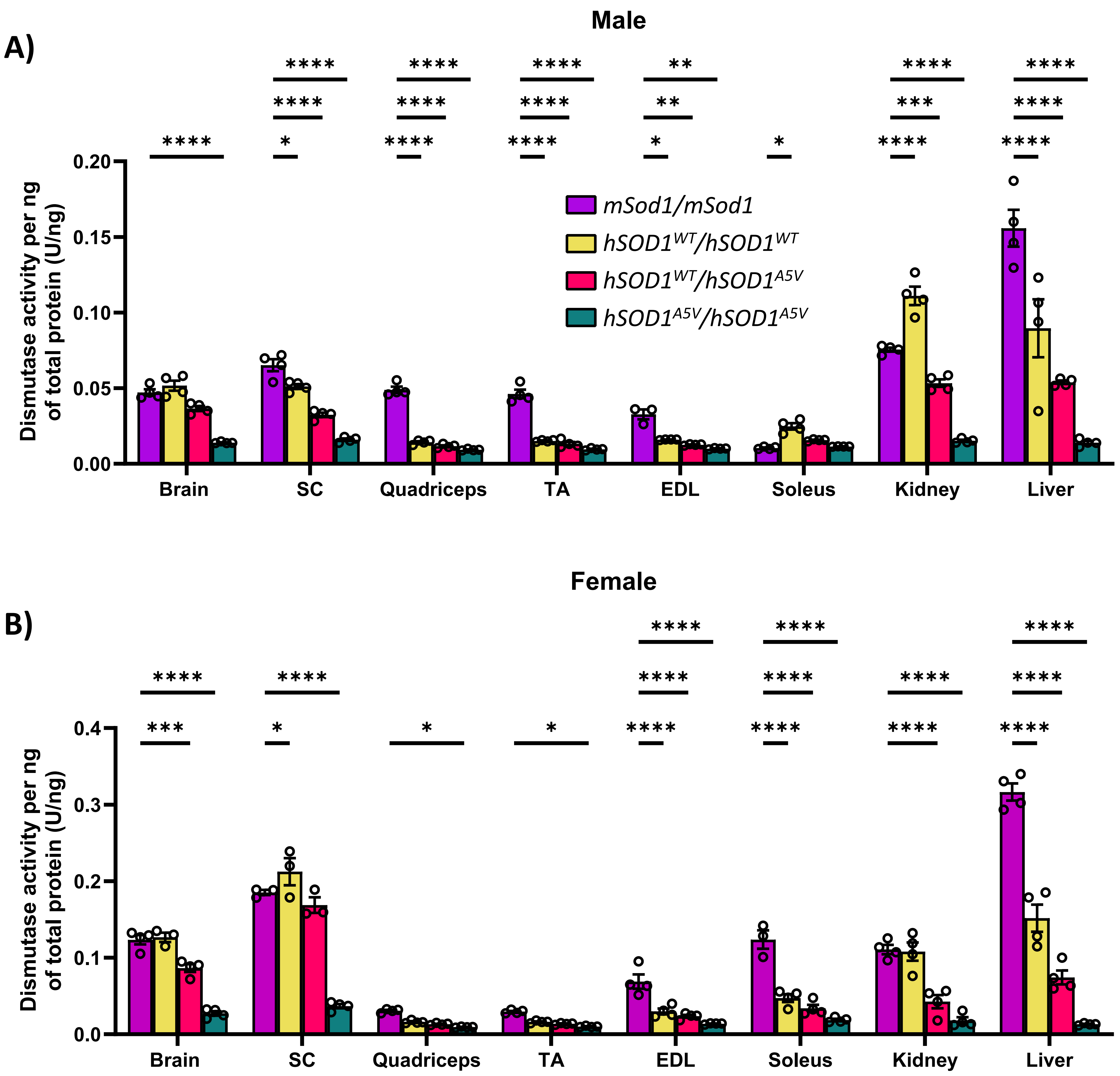

Supplementary Figure 27. Effect of SOD1 humanisation on SOD1 activity. Spectrophotometric dismutase activity assays in tissues from A) male (3-4 animals per genotype) and B) female (3-4 animals per genotype) *hSOD1*^{A5V} mice and 4-month-old *mSod1/mSod1* controls. Mean ± SEM. One-way ANOVA with Tukey's post hoc test. Main effects: A) brain $F(3,12) = 59.0$, $P = 1.86 \times 10^{-7}$; SC $F(3,12) = 86.4$, $P = 2.16 \times 10^{-8}$; Quadriceps $F(3,12) = 258$, $P = 3.71 \times 10^{-11}$; TA $F(3,12) = 116$, $P = 4.00 \times 10^{-9}$; EDL $F(3,11) = 57.1$, $P = 5.42 \times 10^{-7}$; soleus $F(3,12) = 36.8$, $P = 2.49 \times 10^{-6}$; kidney $F(3,12) = 139$, $P = 1.39 \times 10^{-9}$; liver $F(3,12) = 27.9$, $P = 1.08 \times 10^{-5}$; B) brain $F(3,11) = 82.5$, $P = 7.96 \times 10^{-8}$; SC $F(3,9) = 75.2$, $P = 1.08 \times 10^{-6}$; Quadriceps $F(3,12) = 124$, $P = 2.73 \times 10^{-9}$; TA $F(3,12) = 132$, $P = 1.85 \times 10^{-9}$; EDL $F(3,12) = 22.0$, $P = 3.64 \times 10^{-5}$; soleus $F(3,11) = 52.8$, $P = 8.11 \times 10^{-7}$; kidney $F(3,12) = 31.7$, $P = 5.51 \times 10^{-6}$; liver $F(3,12) = 132$, $P = 1.90 \times 10^{-9}$. For clarity, only significant comparisons to *mSod1* controls are shown. $^{*}P < 0.05$, $^{**}P < 0.01$, $^{***}P < 0.001$, $^{****}P < 0.0001$. ns, not significant; SC, spinal cord; TA, tibialis anterior; EDL, extensor digitorum longus.

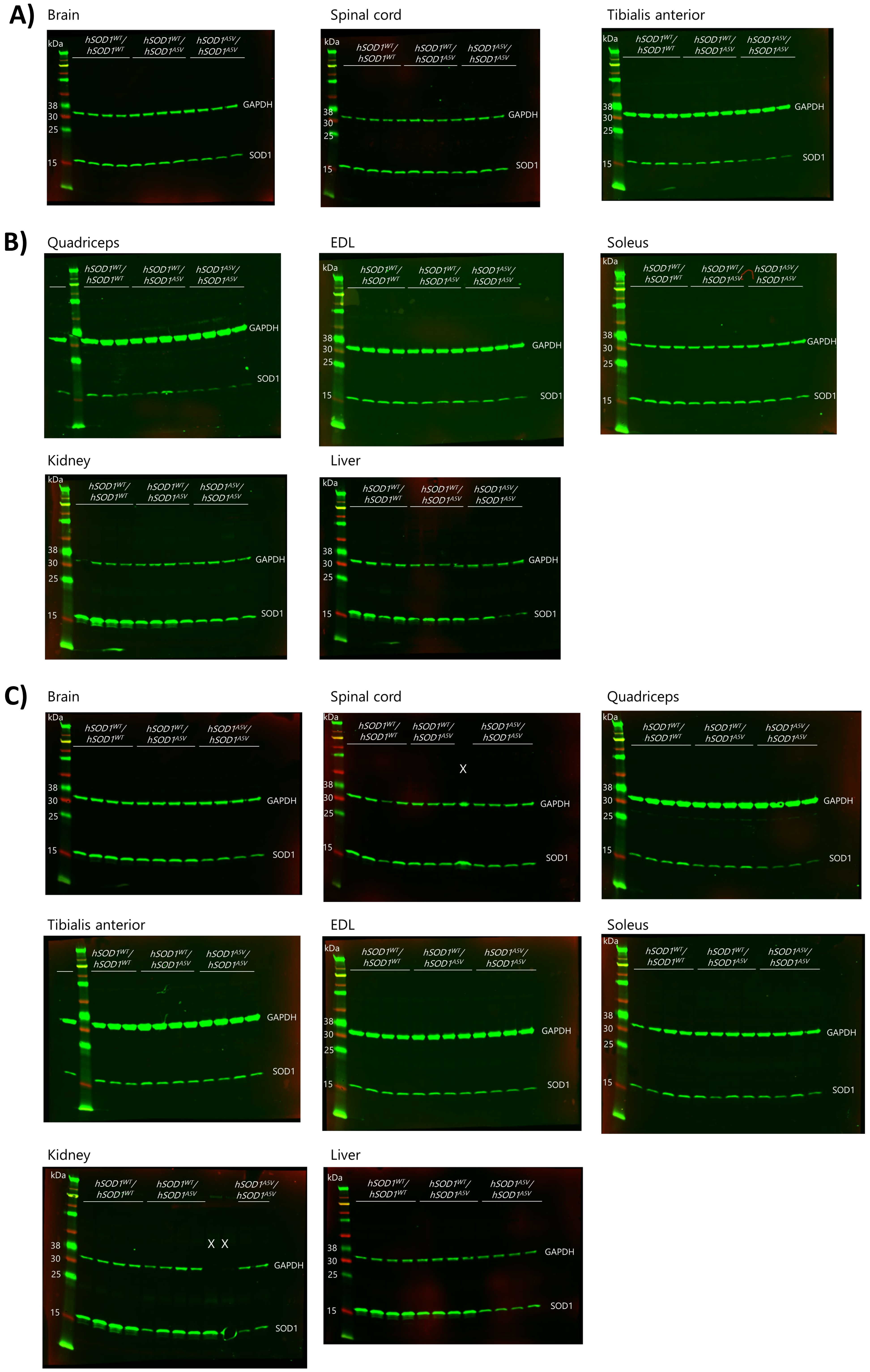

Supplementary Figure 28. Full Western blot images. A) Western blots relating to Figure 1. B) Western blots relating to Supplementary Figure 4. C) Western blots relating to Supplementary Figure 5. EDL, extensor digitorum longus.

SUPPLEMENTARY MATERIAL AND METHODS

Motor Phenotyping

All phenotyping was performed in the light phase (7am-7pm). For all tests, mice were acclimatised to the testing room for at least 30 minutes prior to starting the procedure.

At each time point (6-, 12-, 18- and 24-months-old) motor and behavioural phenotyping tests were performed in the following order: open field (data not shown), combined SHIRPA[1] and dysmorphology (data not shown), gait analysis, Locotronic (data not shown), Rotarod, grip strength, automated wheel running (data not shown).

Accelerating Rotarod test

The accelerating Rotarod test (4–40 r.p.m. over 5 min; Ugo Basile) consisted of 6 trials performed on three days per week over two consecutive weeks. Mice were given one trial per day. The first 4 trials were considered training and no results were recorded. From the final two trials, the longest trial time was recorded. The end of the trial was classified as the time when the mouse fell from the rotarod or made three consecutive passive rotations. The maximum trial duration was 5 minutes.

Grip strength

Grip strength was measured using a grip strength meter (Bioseb). To measure forelimb grip, mice were lifted gently by the base of the tail and held with their forelimbs in contact with the grid of the grip strength meter. Mice were then pulled horizontally away from the mesh in one smooth motion without excessive force and the resulting grip strength value was recorded in grams. To measure forelimb plus hindlimb grip a similar procedure was applied whereby mice were placed to grip the grid with all four limbs. Three measurements of forelimb grip and three measurements of forelimb plus hindlimb grip were collected from each mouse at each time point.

To minimise interoperator variability, at each phenotyping time point the same researcher performed all grip strength measurements. For forelimb, and forelimb plus hindlimb grip strength the median measurement from each animal was divided by the weight of the animal and the resulting value was used for analysis.

Gait analysis

The MouseWalker system is used to examine gait in freely walking rodents, similar to the footprint test. The MouseWalker apparatus is described in Mendes et al.[2] and consists of an illuminated acrylic glass catwalk with a high-speed camera to record mouse foot placement.

The MouseWalker procedure was performed in the dark. Mice were placed at the end of the catwalk and allowed to walk freely along it for 1 minute. Videos captured by the camera were analysed frame by frame to quantify gait parameters.

Metabolic Phenotyping

EchoMRI

EchoMRI measures body composition in conscious animals; providing measurements of whole-body fat mass, lean mass and water content. EchoMRI was performed using a mouse EchoMRI machine (Echo Medical Systems).

Intra-peritoneal glucose tolerance testing (IPGTT)
Mice were fasted overnight prior to the test. EMLA local anaesthetic cream was applied to the tail 30 minutes prior to the test. Blood was sampled from the lateral tail vein and glucose measurements performed using an AlphaTrak meter and AlphaTrak test strips. For the T0 baseline measurement, blood glucose was measured prior to glucose administration. Glucose solution was then administered via IP injection at a dose of 2 g/kg. Blood glucose was measured 15, 30, 60 and 120 minutes after glucose administration. Statistical analysis was performed on data from the individual sampling time points and on the area under the curve (AUC) across all time points.

Monthly weights
Mice were weighed monthly at approximately the same time of day (between 8am and 12pm). Mice that reached 10% weight loss compared to their individual maximum body weight were weighed more frequently (weekly or daily).

Survival
Animal health and wellbeing was assessed daily. The humane end points for this study included: weight loss exceeding 20% of maximum bodyweight, piloerection or other loss of condition, postural abnormalities (e.g. hunched appearance, tremors or ataxia impinging on the ability to feed), abnormal behaviour impinging on the welfare of the animal (e.g. lethargy, extreme hyperactivity), seizures, and age-related loss of condition. Mice culled for common intercurrent illness (e.g. tumours, dermatitis) or as a result of fighting were censored in the survival analysis. The study end point was approximately 650 days old for male animals and 600 days old for female animals. Survival data were analysed by Kaplan-Meier. Statistical significance was determined using a log-rank (Mantel-Cox) test where $P < 0.05$ was considered significant.

Blood sampling for glutathione measurement and metabolomics
Mice were fasted for approximately 15 hours before blood sampling from the lateral tail vein. EMLA cream was applied to the tail 30 minutes prior to blood sampling, then thoroughly removed with an ethanol wipe before a small incision was made at the base of the tail. Between 100 µl and 200 µl blood was collected into heparin-coated tubes. The volume of extracted blood did not exceed 15% total blood volume. 50 µl whole blood was immediately mixed with 50 µl meta-phosphoric acid (MPA) buffer (5% w/v meta-phosphoric acid, 2mM diethylenetriaminepentaacetic acid) and deprotonated on ice for 10 minutes. This whole blood/MPA buffer mix was used for measurement of glutathione. The remaining whole blood was left to coagulate on ice for 40 minutes, then centrifuged at 2000 x g for 10 minutes at 4°C. The plasma and cell pellet were separated, and both were flash frozen in liquid nitrogen and stored at -80°C before analysis.

Prior to metabolomics analysis, frozen plasma from *hSOD1*^{A5V} animals were thawed at room temperature and diluted 1/10 with 90% methanol. The samples were then precipitated at -20°C for 2 hours and centrifuged for 10 minutes at 17,000 x g at 4°C. The supernatants were transferred to glass vials and dried in a SpeedVac concentrator. Quality control samples were generated by pooling a small amount of all experimental samples. Internal standards of known concentration were added to each sample.

Tissue sampling for metabolomics
Flash-frozen tissues, stored at -80°C, were thawed on ice and homogenised in 25 volumes of ice-cold lysis buffer (Phosphate buffered saline (PBS), Complete Protease Inhibitor Cocktail (Roche), 0.5% NP-40). Lysates were centrifuged at 18,000 x g for 30 min at 4°C and the supernatants were

collected and stored at −80°C until analysis. Protein concentration was measured using a DC Protein Assay (BioRad).

Measurement of GSSG/GSH

Reduced glutathione (GSH) and oxidised glutathione (GSSG) levels were measured in whole blood from *hSOD1*^{A5V} animals using LC-MS. Before GSH/GSSG analysis, 25 µl of each sample was mixed with 75 µl extraction solution (1.67% MPA, 3.33 µM GSH as internal standard). GSH and GSSG were annotated in the MS data using a targeted processing approach.

LC-MS

Liquid chromatography–mass spectrometry (LC-MS) experiments were performed by the Swedish Metabolomics Centre. Samples were run on an ultra-high performance liquid chromatography-quadrupole time-of-flight mass spectrometry (UHPLC/Q-TOF-MS) instrument in both positive and negative mode. To minimise the effect of instrumental drift, samples from each time point were run in a random order. Using a targeted data processing approach, 212 metabolites were annotated in samples from 4-13-month-old animals, and 195 metabolites were annotated in samples from 20-month-old animals.

GC-MS

Gas chromatography–mass spectrometry (GC-MS) experiments were performed by the Swedish Metabolomics Centre. Samples were run on a gas chromatography time-of-flight mass spectrometry (GC-TOF-MS) instrument. Samples from each time point were run in a random order. Using a targeted data processing approach, 94 metabolites were annotated in samples from 4-13-month-old animals, and 82 metabolites were annotated in samples from 20-month-old animals.

Histology, immunohistochemistry and immunofluorescence

MN counts

Our protocol for motor neuron counting has been published.[3] Lumbar spinal cords were embedded in OCT and frozen in isopentane over dry ice and stored at -80°C until use. Embedded lumbar spinal cords were sectioned using a cryostat. 20 µm thick transverse sections were cut spanning the L2-L6 region of the lumbar spinal cord and mounted on glass slides. The sections were divided into 3 series such that the distance between sections within each series was 60µm. Sectioning was performed by histology staff at the MRC Harwell institute.

To visualise motor neurons, sections from *hSOD1*^{WT} mice were stained with gallocyanin and sections from *hSOD1*^{A5V} mice were stained with cresyl violet. Stained sections were mounted and imaged on a Nanozoomer slide scanner (Hamamatsu). For each animal, spinal motor neurons in the sciatic pool of the lumbar spinal cord were counted in a minimum of 40 sections spanning the L2-L6 region of the spinal cord. Spinal cord regions L2-L6 were identified from the spinal cord morphology. Motor neurons were differentiated from other cell types by their large size, dense staining pattern and the presence of multiple dendrites. Motor neuron counting was performed in the NDP.view 2 software (Hamamatsu). Analyses are presented as the average number of sciatic motor neurons per section in the L3-L5 region of the spinal cord.

NMJ analysis on lumbrical muscles

The protocol for staining and analysis of NMJs in lumbrical muscles was adapted from Sleigh et al.[4] Hindlimb lumbrical muscles were extracted, fixed for 10 minutes in 4% PFA and stored in PBS until staining. Fixed hindlimb lumbrical muscles in 24 well plates were washed once in PBS for 30 minutes. Muscles were then permeabilised in 2% triton X-100 (v/v) for 30 minutes, followed by a

30-minute incubation in blocking solution (4% bovine serum albumin (BSA) (w/v), 1% triton X-100 (v/v), PBS), and incubation in primary antibodies against neurofilament (2H3; 1:50) and synaptophysin (SV2; 1:100) in blocking solution at 4°C overnight. Muscles underwent 3 x 30-minute washes in PBS, followed by incubation with anti-mouse IgG (1:500, Alexa Fluor 488, ThermoFisher) and α-Bungarotoxin (α-BTX; 1.5 µg/ml; tetramethylrhodamine; Biotium) in PBS for 2 hours at room temperature. Muscles were washed 3 x 30-minutes in PBS and mounted on glass slides in ProLong Gold mounting media (ThermoFisher).

Samples were imaged at 488 nm and 555 nm wavelengths on an LSM 700 confocal microscope at 20x magnification. NMJ analysis on Z-stack maximum intensity projections was performed in ImageJ. NMJs were recorded as innervated or denervated; partial denervation was not assessed due to high non-specific staining with antibodies against 2H3 and SV2 in older animals. At least 40, but usually 100-200 NMJs were counted from each animal.

NMJ analysis on TA and soleus muscles

The TA and soleus muscles from 6-month-old *hSOD1*^{A5V} animals were injected with tetanus toxin heavy chain (atoxic fragment of tetanus neurotoxin, H_CT) as part of axonal transport experiments; H_CT labels neuronal endosomes and hence stains the axon/presynapse without the need for antibodies. The TA and soleus muscles from 24-month-old mice were labelled using antibodies against SV2/2H3.

The protocol for processing of TA and soleus muscles for NMJ analysis was adapted from Bolatto et al.[5] TA and soleus muscles were fixed for 30-180 minutes in 4% PFA, teased apart into bundles of approximately 5-20 fibres and transferred to PBS in 24 well plates. Fibre bundles were washed once in PBS for 20 minutes. Fibre bundles were then stained with α-BTX (1:500, Alexa Fluor 488 or tetramethylrhodamine) for 1 hour at room temperature. Fibre bundles from 24-month-old animals were permeabilised in 2% Triton X-100 (v/v) for 90 minutes, blocked in 4% BSA (w/v) and 1% Triton X-100 (v/v) for 30 minutes and incubated in primary antibodies against 2H3 (1:50) and SV2 (1:100) in blocking solution at 4°C overnight. Fibre bundles from 24-month-old animals were washed 4 x 20 minutes in PBS before incubation in anti-mouse IgG (1:250, Alexa Fluor 488, ThermoFisher) for 1 hour at room temperature. All fibre bundles were then washed 4 x 20 minutes in PBS and mounted on glass slides in ProLong Gold mounting media (ThermoFisher).

Samples were imaged at 488 nm and 555 nm wavelengths on an LSM 700 confocal microscope at 20x magnification. NMJ analysis on Z-stack maximum intensity projections was performed in ImageJ. NMJs were recorded as innervated or denervated; partial denervation was not assessed due to poor quality of staining of the postsynaptic NMJ with α-BTX. At least 40 NMJs were counted from each muscle.

Muscle fibre typing

Muscle ATPase staining was performed to differentiate type I, II and IIC muscle fibres. Flash frozen TA muscles were cut into 12 µm thick transverse sections on a cryostat. Sections were preincubated in sodium barbital solution at pH 4.5 (35 mM sodium barbital, 60 mM sodium acetate, HCl) or pH 10.8 (20 mM sodium barbital, 36 mM $CaCl_2$, NaOH). Sections were rinsed once in distilled water followed by incubation in ATP solution (20 mM sodium barbital, 9 mM $CaCl_2$, 2.7 mM ATP; pH 9.5). Sections were then washed 3 x 3 minutes in 1% $CaCl_2$ (w/v) before incubation for 10 minutes in 2% $CoCl_2$ (w/v). Sections were washed in 5 changes of 5 mM sodium barbital. Sections were then incubated in 1% ammonium sulphide (v/v) for 30 seconds, washed in 6 changes of water and mounted using Aquamount (Life Technologies). Stained sections were digitalised on a

Nanozoomer slide scanner (Hamamatsu). Muscle fibre type was assessed in a 750 µm x 750 µm area positioned at approximately the same location within each section. For each animal, muscle fibre type was assessed at pH 4.5 and pH 10.8 on an average of 2 sections spaced 400 µm apart.

Determination of muscle fibre size and central nucleation

Fresh frozen TA muscles were cut into 8 µm thick transverse sections on a cryostat. Sections were transferred to glass slides, stained with Haemotoxylin and Eosin (H&E), and digitized using a slide scanner (Zeiss Axio Scan.Z1). For each muscle, three 500 µm x 500 µm regions were selected for determination of muscle fibre size. Segmentation of muscle fibres was performed in Ilastik[6] and ImageJ (National Institutes of Health) and checked manually. The minimum Feret diameter for each fibre was calculated in ImageJ. Central nucleation was assessed manually in 500 fibres per muscle.

***In vivo* axonal transport**

Intramuscular injections of H_CT

A fluorescently labelled atoxic fragment of tetanus neurotoxin (H_CT) was prepared (H_CT residues 875–1315 fused to a cysteine-rich region and a human influenza haemagglutinin epitope), and subsequently labelled with Alexa Fluor 555 C2 maleimide (Thermo Fisher Scientific, A-20346), as previously described.[7,8] Mice were anesthetized using isoflurane, fur on the lower leg was shaved, and mice were placed on a heat-pad ready for intramuscular injections. Upon the absence of reflexes, a small incision was made above the upper tibialis anterior muscle (TA) of both hindlimbs. Guided by a previously established motor end plate map,[9] injections were made into both the left and right TA with 7.5–10 mg of H_CT in PBS in a volume of ~3.5 µL using pulled graduated, glass micropipettes (Drummond Scientific, 5-000-1001-X10), as previously described.[10] When complete, the skin was sutured, and mice were monitored for up to 1 h before returning to the home cage.

In vivo imaging of signalling endosomes

To visualise signalling endosome transport *in vivo,* time-lapse confocal microscopy of motile H_CT-containing signalling endosomes in TA-innervating motor axons was performed, as described previously.[7,8] At least 4 h after H_CT intramuscular injections, mice were re-anaesthetised with isoflurane, and the sciatic nerves were exposed with the removal of overlying skin, musculature and connective tissue, and a small piece of parafilm was inserted to separate the sciatic nerve from the underlying connective tissue/musculature. The mouse and anaesthetic nosepiece were then transferred to a customised stage on an inverted LSM780 confocal microscope (Zeiss) enclosed within an environmental chamber maintained at 37^0C. Time-lapse microscopy was performed using a 40x, 1.3 NA DIC Plan-Apochromat oil-immersion objective (Zeiss) focusing on thicker axons using an 80x digital zoom (1024 x 1024, <1% laser power). Only the axons that contained H_CT-containing signalling endosomes were selected for signalling endosome transport, with areas containing the node of Ranvier purposely avoided.[11] Frame intervals of 1.0 – 1.3 s were used when acquiring transport videos of motile H_CT-positive signalling endosomes. When the first leg was complete, the same process was repeated for the contralateral limb. All imaging was concluded within a maximum of 90min from initiating re-anaesthesia.

Tracking analysis

Confocal ''.czi'' images were opened in FIJI (http://rsb.info.nih.gov/ij/), and converted to ''.tiff''. Using the TrackMate plugin,[12] H_CT-positive signalling endosomes were tracked manually,[11,13] using the following criteria: 1) organelles moving for >10 consecutive frames were included, while terminal pauses, which we defined as the absence of movement in >10 consecutive frames, were excluded. In addition, tracking never commenced on an immotile organelle; 2) a minimum of 20

signalling endosomes were selected per axon; 3) at least 3 individual axons were assessed per limb. A pause was defined as a previously motile organelle with a velocity of <0.1 mm/s between consecutive frames (to account for a potential breathing/vasculature artifact), and the time paused (%) was defined by dividing the number of pauses by the total number of frame-to-frame movements assessed from an individual axon.

Biochemical and molecular analyses

RNA extraction for Reverse transcription quantitative PCR (RT-qPCR) and RNA sequencing (RNA-seq)

Following RNA extraction, RNA quantity was assessed using a NanoDrop Spectrophotometer (ThermoFisher). RNA quality was assessed using a 2100 Bioanalyzer (Agilent) where an RNA integrity number (RIN) of >7 was deemed acceptable for RT-qPCR and RNA-seq. Extracted RNA was stored at -80°C until use.

Reverse transcription quantitative PCR (RT-qPCR)

Complementary DNA (cDNA) was generated using a High-Capacity cDNA Reverse Transcription Kit (Applied Biosystems). 10 ng cDNA was used to perform RT-qPCR in triplicate, using 5 µl cDNA, 10 µl Fast SYBR Green Master Mix (Thermo Fisher) and 0.2 µM each forward and reverse primer in a total reaction volume of 20 µl. RT-qPCR was performed on a 7500 Fast Real-Time PCR System (Applied Biosystems) using the following conditions: 95 °C for 10 minutes, 40 cycles of 95 °C for 15 seconds, 60 °C for 1 minute. The sequences of primers used in this study are listed in Table 3. Relative quantification of gene expression was performed using the $2^{-\Delta\Delta CT}$ method. *Gapdh* was used as a housekeeping gene for normalisation of *SOD1* gene expression.

Protein extraction from mouse tissues

Tissues were thawed on ice and homogenised in 25 volumes of ice-cold lysis buffer (PBS, Complete Protease Inhibitor Cocktail (Roche), 0.5% NP-40). Lysates were centrifuged at 18,000 x g for 30 min at 4°C and the supernatants were collected and stored at −80°C until analysis. Protein concentration was measured using a DC Protein Assay (BioRad).

Western blotting

Ten to fifteen micrograms of protein per sample was used for reducing SDS-PAGE on Novex 14% Tris-Glycine gels (ThermoFisher) followed by Western blotting. Blots were probed with a pan-SOD1 antibody raised against a peptide corresponding to amino acids 131-153 of the SOD1 protein, which are identical in human and mouse SOD1, followed by IRDye 680RD conjugated secondary antibody (LI-COR)[14]. SOD1 immunofluorescence was normalised to the total protein content of each sample, measured using a Revert 700 Total Protein Stain (LI-COR). Images were taken using an Odyssey CLx imager (LI-COR).

In-gel dismutase activity assays

The protocol for in-gel dismutase activity assays was adapted from Weydert & Cullen.[3] 15-30 µg of protein per sample was separated using clear native PAGE (CN-PAGE) on 3-12% NativePAGE Bis-Tris gels (ThermoFisher). Following CN-PAGE, gels were soaked in SOD1 stain solution (50 mM potassium phosphate, 336 µM nitro blue tetrazolium, 170 µM riboflavin, 21 mM TEMED) in the dark for 45 minutes. Gels were then rinsed with ddH_2O twice and placed under a fluorescent light for approximately 1 hour, during which time clear bands corresponding to areas of SOD1 activity began to appear. Gels were then washed 3 times with ddH_2O and left under ambient light for 18-24 hours to allow for further band development. Gels were imaged using a ChemiDoc XRS+ imaging system (BioRad) with a white light conversion filter.

Spectrophotometric dismutase activity assays

Protein samples were thawed on ice and dispensed into 96-well assay plates. Depending on the tissue, 1-5 µg of protein per sample was used for dismutase activity assays. 200 µL of reagent mixture (100 mM pH 8.0 sodium phosphate, 100 µM DTPA, 10 mM hypoxanthine, 50 µM WST-1, 10 µg/mL catalase, 0.1 units/mL xanthine oxidase (Sigma)) was added to every well using a multichannel pipette. Plates were shaken for 30 seconds to mix then absorbance was read immediately at 438 nm wavelength every 40 seconds for 3 minutes at 25°C SOD1 activity was determined based on comparison to 12-point standard curve of bovine SOD1 ranging from 1-1000 ng/well. 1 unit of activity was defined as the amount of bovine SOD1 required for 50% inhibition of tetrazolium reduction (approximately 25 ng).

ELISA for soluble disordered SOD1 (misELISA)

ELISA (enzyme-linked immunosorbent assay) plates (Maxisorp, Nunc) were coated overnight at room temperature with 640 ng/mL of anti-SOD1 (human) monoclonal antibody (raised against amino acids 24-39, exposed only in disordered SOD1) in 100 mM Tris-HCl. After washing (PBS and 0.05% Tween-20) and blocking, 25 µg of protein samples and apo-SOD1 standards diluted in PBS, protease inhibitor and 40 mM iodoacetamide were incubated for 90 min at room temperature. Each well was washed thrice before addition of 1.5 µg/ml goat antibody, raised against mouse SOD1 for 1 h at room temperature. After washing, anti-goat HRP (Invitrogen, 1:5000) was added for 1 h at room temperature. Finally, after washing, wells were incubated in TMB substrate for 15 minutes. Sulphuric acid (2M) was added to stop the reaction and the absorbance read at 450 nm.

Data Analysis

Transcriptomics data analysis

Quality control and read trimming was performed with Trimmomatic version 0.36. Trimmed reads were then aligned to the mm39 mouse reference genome with HISAT2 (Version 2.1.0). FeatureCounts was used to count exon spanning reads. One *hSOD1*$^{WT/WT}$ sample from the 6-month-old TA muscle dataset clearly deviated from all other samples in PCA and was excluded from downstream analyses. Differential gene expression (DE) analysis was performed using DESeq2 (Version 1.40.1). Average gene expression was obtained for n=4 animals in each genotype/time point and the proportion of DEGs between groups was determined using the Benjamini and Hochberg procedure implemented in DESeq2 for correction of p-values with false discovery rate (FDR). To identify genes considered potentially biologically relevant, analysis was also conducted using a p-value of <0.01. DESeq2 output files were exported and volcano plots developed in GraphPad Prism 10. The UpsetR package in R Studio was used to design Venn plots. Enrichr[15,16] was used to identify significantly enriched GO terms and infer canonical pathways regulated by the identified DEGs. Chord plots were constructed according to Tang et al.[17]

Integrated transcriptomic and metabolomic analysis

Integrated network analysis and visualisation of transcriptomic and metabolomic data allowed for the exploration of relationships between DEGs and significantly differentially expressed metabolites (DEMs) and associated metabolic pathways, providing a comprehensive view of the biological processes affected. DEGs were uploaded to the STRING database within Cytoscape (STRING v.11, Cytoscape v.3.10.2). STRING was used to predict protein-protein interactions (PPIs) based on various evidence sources, including experimental data, computational predictions, and text mining. The confidence score for PPIs was set to 0.4 to retain only interactions with sufficient reliability. The STRING network was queried against Reactome pathways to identify over-represented pathways associated with the DEGs and DEMs (Reactome v.91).[18] Enriched pathways

were visualized using BioRender to create a schematic representation of the key pathways and interactions.

SUPPLEMENTARY REFERENCES

1. Rogers DC, Peters J, Martin JE, et al. SHIRPA, a protocol for behavioral assessment: validation for longitudinal study of neurological dysfunction in mice. *Neurosci Lett*. 2001;306(1-2):89-92. doi:10.1016/S0304-3940(01)01885-7

2. Mendes CS, Bartos I, Márka Z, Akay T, Márka S, Mann RS. Quantification of gait parameters in freely walking rodents. *BMC Biol*. 2015;13(1):50. doi:10.1186/s12915-015-0154-0

3. Weydert CJ, Cullen JJ. Measurement of superoxide dismutase, catalase and glutathione peroxidase in cultured cells and tissue. *Nat Protoc*. 2010;5(1):51-66. doi:10.1038/nprot.2009.197

4. Sleigh JN, Burgess RW, Gillingwater TH, Cader MZ. Morphological analysis of neuromuscular junction development and degeneration in rodent lumbrical muscles. *J Neurosci Methods*. 2014;227:159-165. doi:10.1016/j.jneumeth.2014.02.005

5. Bolatto C, Olivera-Bravo S, Cerri S. Dissection of Single Skeletal Muscle Fibers for Immunofluorescent and Morphometric Analyses of Whole-Mount Neuromuscular Junctions. *J Vis Exp*. 2021;(174):62620. doi:10.3791/62620

6. Berg S, Kutra D, Kroeger T, et al. ilastik: interactive machine learning for (bio)image analysis. *Nat Methods*. 2019;16(12):1226-1232. doi:10.1038/s41592-019-0582-9

7. Sleigh JN, Tosolini AP, Gordon D, et al. Mice Carrying ALS Mutant TDP-43, but Not Mutant FUS, Display In Vivo Defects in Axonal Transport of Signaling Endosomes. *Cell Rep*. 2020;30(11):3655-3662.e2. doi:10.1016/j.celrep.2020.02.078

8. Tosolini AP, Villarroel-Campos D, Schiavo G, Sleigh JN. Expanding the Toolkit for In Vivo Imaging of Axonal Transport. *J Vis Exp*. 2021;(178):63471. doi:10.3791/63471

9. Mohan R, Tosolini AP, Morris R. Targeting the motor end plates in the mouse hindlimb gives access to a greater number of spinal cord motor neurons: An approach to maximize retrograde transport. *Neuroscience*. 2014;274:318-330. doi:10.1016/j.neuroscience.2014.05.045

10. Mohan R, Tosolini AP, Morris R. Segmental distribution of the motor neuron columns that supply the rat hindlimb: A muscle/motor neuron tract-tracing analysis targeting the motor end plates. *Neuroscience*. 2015;307:98-108. doi:10.1016/j.neuroscience.2015.08.030

11. Tosolini AP, Abatecola F, Negro S, Sleigh JN, Schiavo G. The node of Ranvier influences the in vivo axonal transport of mitochondria and signaling endosomes. *iScience*. 2024;27(11):111158. doi:10.1016/j.isci.2024.111158

12. Ershov D, Phan MS, Pylvänäinen JW, et al. TrackMate 7: integrating state-of-the-art segmentation algorithms into tracking pipelines. *Nat Methods*. 2022;19(7):829-832. doi:10.1038/s41592-022-01507-1

13. Tosolini AP, Sleigh JN, Surana S, Rhymes ER, Cahalan SD, Schiavo G. BDNF-dependent modulation of axonal transport is selectively impaired in ALS. *Acta Neuropathol Commun*. 2022;10(1):121. doi:10.1186/s40478-022-01418-4

14. Jonsson PA, Ernhill K, Andersen PM, et al. Minute quantities of misfolded mutant superoxide dismutase-1 cause amyotrophic lateral sclerosis. *Brain J Neurol*. 2004;127(Pt 1):73-88. doi:10.1093/brain/awh005

15. Kuleshov MV, Jones MR, Rouillard AD, et al. Enrichr: a comprehensive gene set enrichment analysis web server 2016 update. *Nucleic Acids Res*. 2016;44(W1):W90-W97. doi:10.1093/nar/gkw377

16. Chen EY, Tan CM, Kou Y, et al. Enrichr: interactive and collaborative HTML5 gene list enrichment analysis tool. *BMC Bioinformatics*. 2013;14(1):128. doi:10.1186/1471-2105-14-128

17. Tang D, Chen M, Huang X, et al. SRplot: A free online platform for data visualization and graphing. Yin Y, ed. *PLOS ONE*. 2023;18(11):e0294236. doi:10.1371/journal.pone.0294236

18. Milacic M, Beavers D, Conley P, et al. The Reactome Pathway Knowledgebase 2024. *Nucleic Acids Res*. 2024;52(D1):D672-D678. doi:10.1093/nar/gkad1025
